# Synthesis and Stereodynamic and Emission Properties of Dissymmetric Bis-Aryl Carbazole Boranes and Identification of a CPL-Active B–C Atropisomeric Compound

**DOI:** 10.1021/acs.joc.2c02209

**Published:** 2023-01-04

**Authors:** Daniel Pecorari, Emanuele Giuliani, Andrea Mazzanti, Stefano Stagni, Valentina Fiorini, Giulia Vigarani, Francesco Zinna, Gennaro Pescitelli, Michele Mancinelli

**Affiliations:** † Department of Industrial Chemistry “Toso Montanari”, University of Bologna, Viale del Risorgimento 4, 40136 Bologna, Italy; ‡ Department of Chemistry and Industrial Chemistry, University of Pisa, Via Moruzzi 13, 56124 Pisa, Italy

## Abstract

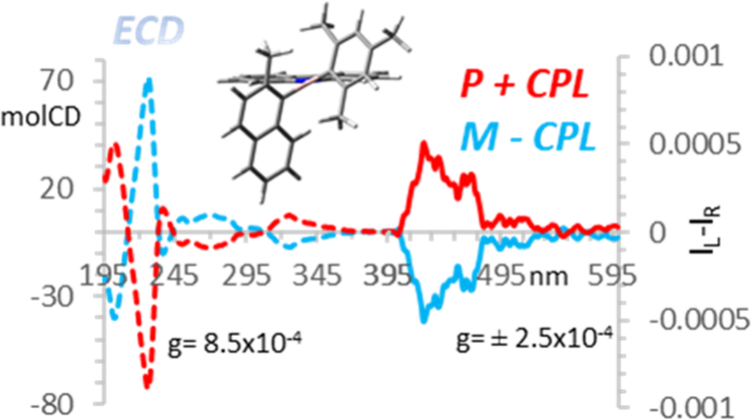

We synthesized bis-aryl carbazole borane derivatives having emissive properties and axial chirality. The resolution of a thermally stable atropisomeric pair (compound **1b**), due to a B–C chiral axis, was achieved by chiral stationary-phase high-performance liquid chromatography (CSP-HPLC). Complete photophysical properties of all compounds were measured and simulated by time-dependent density functional theory (TD-DFT) calculations of the complete fluorescence cycle, and circularly polarized luminescence spectra were obtained for the atropisomers of compound **1b**, whose absolute configuration was derived using a TD-DFT simulation of the electronic circular dichroism (ECD) spectra.

## Introduction

Bis-Mesityl carbazole boranes (or aminoboranes) have been widely studied recently because of their twisted intramolecular charge transfer (TICT) emissive properties.^1^ Many applications of aminoboranes in materials science have been reported, such as OLED preparation,^2^ solid-state mechanofluorochromism,^3^ thermally activated delayed fluorescence emitters,^4^ organoboron-based materials in nonlinear optics,^5^ and boron-based stimuli-responsive materials.^6^ They have also been proposed as fluorescent probes for CO_2_ monitoring and detection.^7^ In our recent work, a series of highly twisted benzocarbazole boranes were prepared to investigate the strength of the π-contribution to the boron–nitrogen bond, which was found to peak at 24 kcal/mol when the heteroaromatic ring was a carbazole.^1a^ The twist angle observed in the ground state (GS) between the heteroaromatic ring and the borane branch was then related to the emissive properties of these derivatives. In general, strong TICT phenomena and noticeable solvatochromic effects were correlated to the large geometric differences between the GS and the relaxed excited state, where the B–N twist angle between the donor and acceptor is afforded by a less efficient π-contribution. The highest occupied molecular orbitals (HOMOs) calculated for the emission step were typically found on the carbazole ring, while the lowest unoccupied molecular orbitals (LUMOs) were primarily found on the B(Mes)_2_ moiety.^1a^

Therefore, we envisaged that substituting one mesityl ring of the borane branch with a more extended aromatic system could modify the TICT rearrangement properties and the nature of the HOMO and LUMO, thus changing the photophysical properties of these compounds. Additionally, an asymmetric aromatic ring at the boron center paves the way for the preparation of atropisomeric aminoboranes with a C–B chiral axis^8^ and hence to the preparation of circularly polarized luminescence (CPL)-active compounds.^9^

**Scheme 1:**
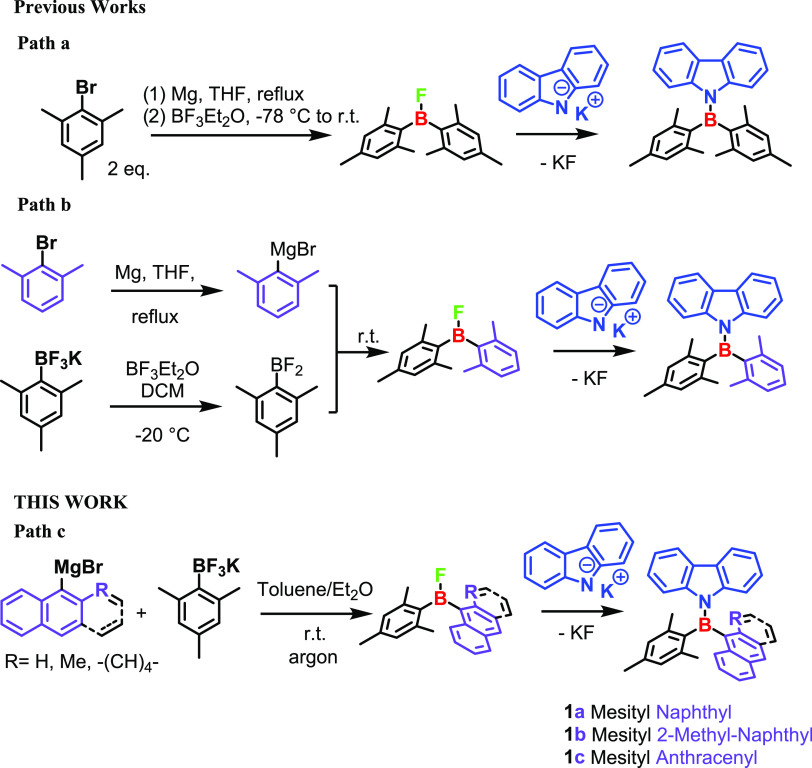
Some Reaction Pathways for Obtaining Bis-Aryl Aminoboranes

Many synthetic pathways have been proposed for preparing bis-mesityl aminoboranes.^1a,3,4,10^ One of the most efficient methods generated a bis-mesityl halogen borane in situ using a Grignard reagent and BF_3_OEt_2_ (path a, Scheme 1).^1a^ The reaction of this intermediate with potassium carbazol-9-ide eventually affords the product. The two *ortho*-methyls of the mesityl rings are known to be crucial for chemical stability because they sterically shield the boron site from nucleophilic attack. Although we previously reported an example of a dissymmetric compound (Path b, Scheme 1),^1a^ both aromatic rings still contained two *ortho*-methyls. Our challenge is to prepare chiral bis-aryl carbazole boranes bearing a more sterically demanding aromatic system, such as naphthyl (**1a**), 2-methyl-naphthyl (**1b**), or anthracene (**1c**), together with a mesityl group to preserve the chemical stability.

## Results and Discussion

The desired compounds (**1a–c**, path c, Scheme 1) were prepared according to the procedure reported in our previous work (path b),^1a^ where potassium mesityl trifluoroborate (MesBF_3_K)^11^ was selected as the boron source. The activation of mesityl trifluoroborate to afford mesityl difluoroborate, which is the electrophile in the Grignard reaction, is a crucial step in the synthesis of compounds **1a–c**. Many fluorophore sources, such as BF_3_OEt_2_,^12^ TMSCl,^13^ and AsF_5_,^14^ have been studied for the activation of mesityl trifluoroborate. In 1995, Vedejs et al. reported that Mg^2+^ and Li^+^ cations could be used as fluorophores to activate mesityl trifluoroborate.^4^

To avoid using the corrosive BF_3_OEt_2_ and to reduce the reaction steps, we tried a reaction by activating the mesityl trifluoroborate with a Grignard reagent under an argon flow.^15^ This allowed us to use very cheap reagents such as aryl bromines and a stable and easy-to-handle boron source such as MesBF_3_K.

**Scheme 2:**
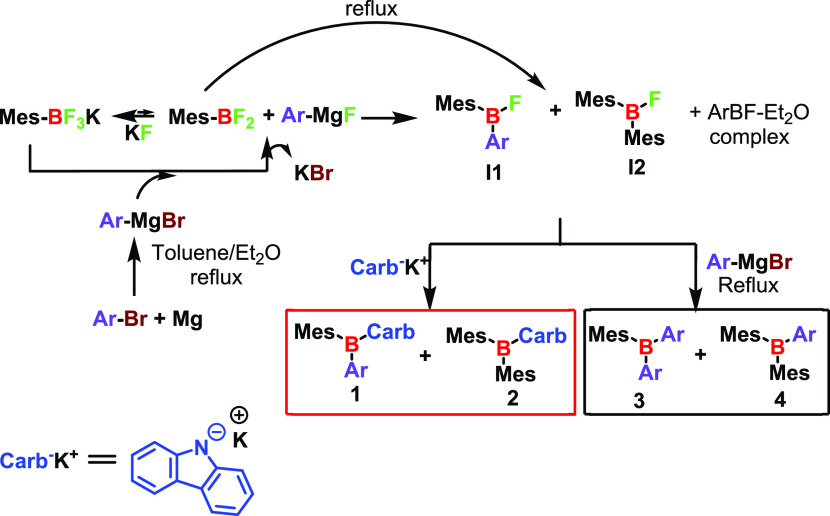
Mechanism Hypothesis for Synthesizing mes-Aryl Fluoroborate, Compound **1**, and Byproducts

The mes-aryl fluoroborate (**I1** in Scheme 2) was synthesized by adding MesBF_3_K to a freshly prepared Grignard reagent at ambient temperature. After 1 h, ^19^F NMR confirmed the complete formation of mes-aryl fluoroborate (Figure S1, bottom). Moreover, a one-step synthesis was attempted (see details in the Supporting Information) by keeping aryl bromine, MesBF_3_K, and magnesium in the same flask and refluxing the mixture to afford an in situ Grignard reagent (method 1 in the Supporting Information). However, following this methodology, the desired products were synthesized with low yields, and many byproducts were formed (Figure S1, top, and Table S1).

The hypothesized mechanism for the reaction is shown in Scheme 2.

Under dry conditions, MesBF_3_K was in equilibrium with MesBF_2_ + KF, and the equilibrium shifted toward the salt.^10,16^ When the Grignard was prepared, Mg^2+^ could act as a fluorophore by exchanging the halogens (Br vs F) in the Grignard reagent and precipitating KBr from the solution. In this way, the equilibrium shifted to the more reactive MesBF_2_, which readily reacts with the aryl nucleophile, yielding the desired aryl-mesityl fluoroborate **I1**. To avoid the dismutation of mesityl difluoroborate into bis-mesityl fluoroborate **I2** at the high temperatures required for the preparation of the Grignard reagent (one-step synthesis in the Supporting Information), MesBF_3_K was added at room temperature.^17^ This also reduced the possibility of intermediates **I1** and **I2** reacting with another molecule of the Grignard reagent at high temperatures to yield undesired compounds **3** and **4**, respectively. In the last step of the desired pathway, intermediate **I1** reacted with potassium carbazol-9-ide, yielding compound **1**, while the intermediate **I2** afforded the byproduct bis-mesityl carbazole **2**.

The carbazole was deprotonated using potassium bis(trimethylsilyl)amide (KHMDS) and various aryl bromides (1-bromo-naphthalene **a**, 1-bromo-2-methyl-naphthalene **b**, and 9-bromo-anthracene **c**) were reacted with it. The reaction afforded the desired compounds **1a–c** in acceptable yields, but the lack of steric hindrance on one side of the 1-naphthyl ring makes compound **1a** more unstable than others (Table S1). For the same reason, during the synthesis of compound **1a**, we observed the formation of tris-aryl borane **3** (Scheme 2) owing to the more reactive 1-naphthyl-magnesium bromide, which pushed the reaction with **I1**. To reduce this reaction, we reversed the roles of the reagents and used mesityl bromide as the precursor for the Grignard reagent and 1-naphthyl-BF_3_K to prepare 1-naphthyl-BF_2_. By this route, the steric hindrance of the mesityl moiety made the Grignard reagent less reactive toward the **I1** intermediate, increasing the final yields and affording a cleaner reaction. This useful interchanging of reagents allowed the preparation of different products based on the reactivity of the aryl Grignard reagents.

## Conformational Analysis

In a previous work,^1a^ the geared structure of bis-mesityl carbazole and benzocarbazole boranes was analyzed in detail from a dynamic point of view using the “ring flip” nomenclature proposed by Mislow for tris-aryl boranes.^18^ The carbazole ring has been bound to boron in a planar arrangement to gain a better disposition of the ^–^B = N^+^ π-system, whereas the two mesityl groups were twisted to yield two enantiomeric conformations in fast exchange at ambient temperature. Rationalization of the dynamic stereochemistry resulted in the identification of a pair of “two-ring flip” (2-RF) transition states (**TS**) related to the B–N and B–C bond rotations, which depended on the disposition of the rings with respect to a reference plane containing nitrogen, boron, and the two quaternary carbons of the two aromatic rings.^1a^

**Figure 1:**
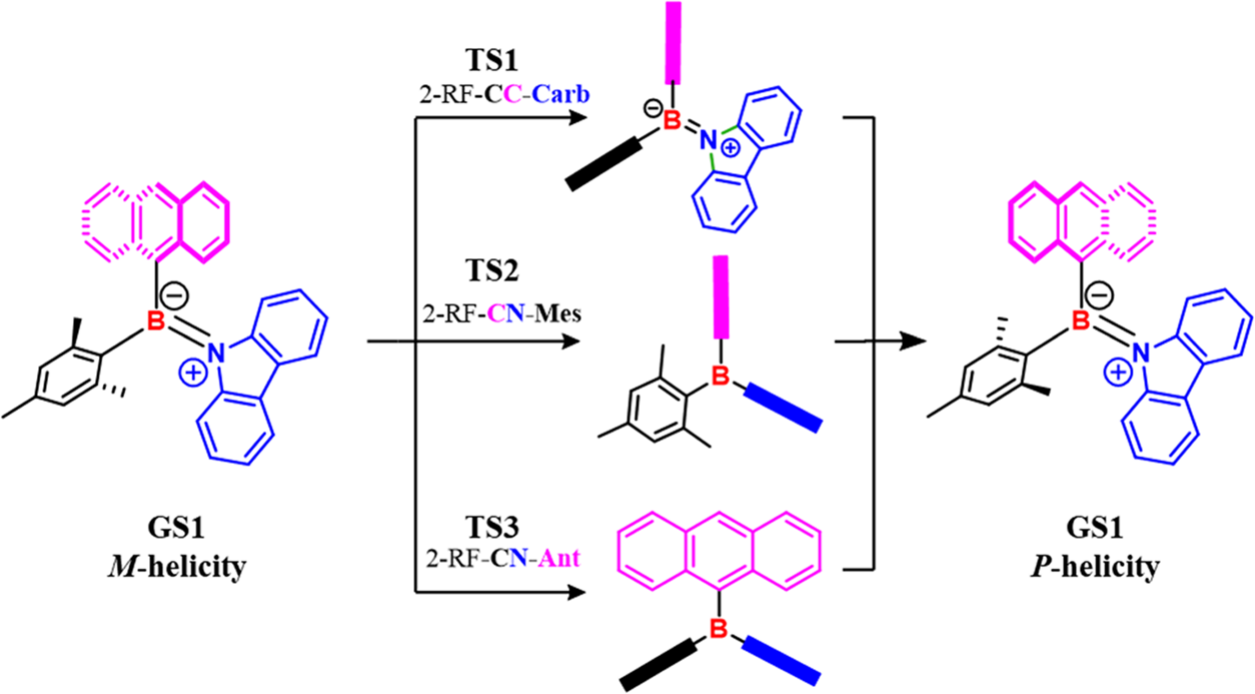
Available ground states (**GSs**) and transition states (**TSs**) for compound **1c**.

The presence of two different aromatic rings on boron did not change either the gear system or the representation of the two-ring flip torsional motion, but it increased the number of available **TSs** for the conjugative and steric barriers. Three 2-RF **TSs** were feasible when the second aromatic ring (the first one is mesityl) was still *C*_2_-symmetric, such as anthracen-9-yl (compound **1c**). The steric barrier had planar carbazole, and the two aromatic rings were perpendicular (**TS1** 2-RF-**CC-Carb**, in Figure 1). Two different geometries of the **TS** were instead available for the conjugative barrier, depending on which of the two aromatic rings on boron lay on the reference plane (**TS2** 2-RF-**CN-Mes** and **TS3** 2-RF-**CN-Ant** in Figure 1), and carbazole was perpendicular to the reference plane in both cases.

**Figure 2:**
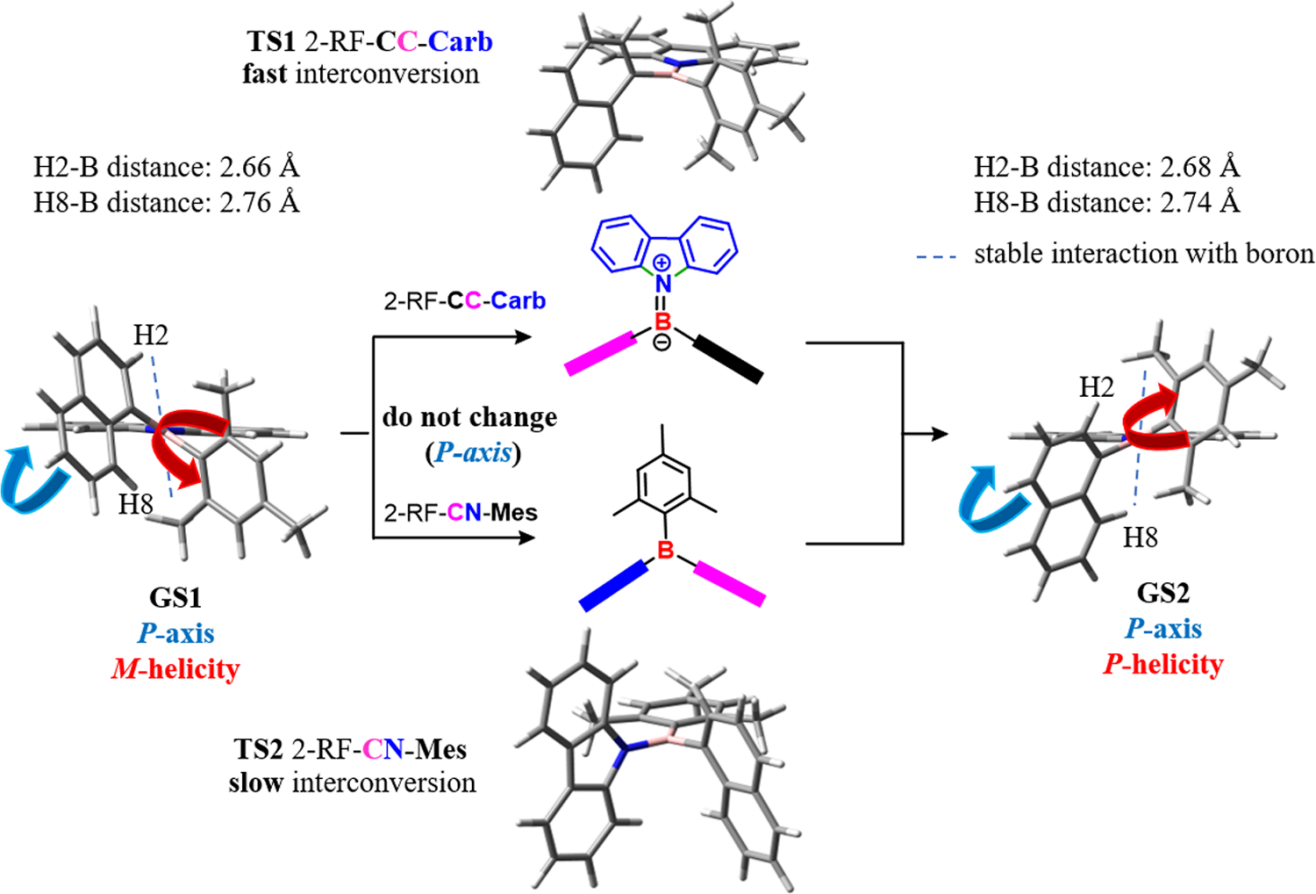
Available **GSs** and **TSs** for the helicity exchange in compounds **1a–b**. The DFT-optimized structures are those of **1a**. See the Supporting Information for the three-dimensional structures of **1b**.

On the contrary, compounds **1a** and **1b** generated a more complex dynamic stereochemistry due to the rotational asymmetry of the naphthyl ring with an increase in the number of available **GSs** and **TSs**. Additionally, it has to be considered that the hindered rotation about the naphthyl-boron bond yielded a chiral axis as an additional stereogenic element in the system. Therefore, in the **GS**, two diastereomeric helical conformations of the same *P* or *M* atropisomer were generated by the different disposition of the naphthyl ring (Figure 2), which could have the boron atom close to the H2 hydrogen (**GS1**, CH_3_ for the 2-methylnaphthyl) or close to H8 (**GS2**).

The interconversion between these two diastereomeric conformations (the axial chirality of the B–C bond was kept constant) could happen due to the two diastereomeric 2-RF **TSs**, where the two aromatic rings were perpendicular to carbazole (**TS1,** 2-RF-**CC-Carb** in Figure 2), or by **TS2** (2-RF-**CN-Mes**), where mesityl is on the reference plane and carbazole is perpendicular.

**Figure 3:**
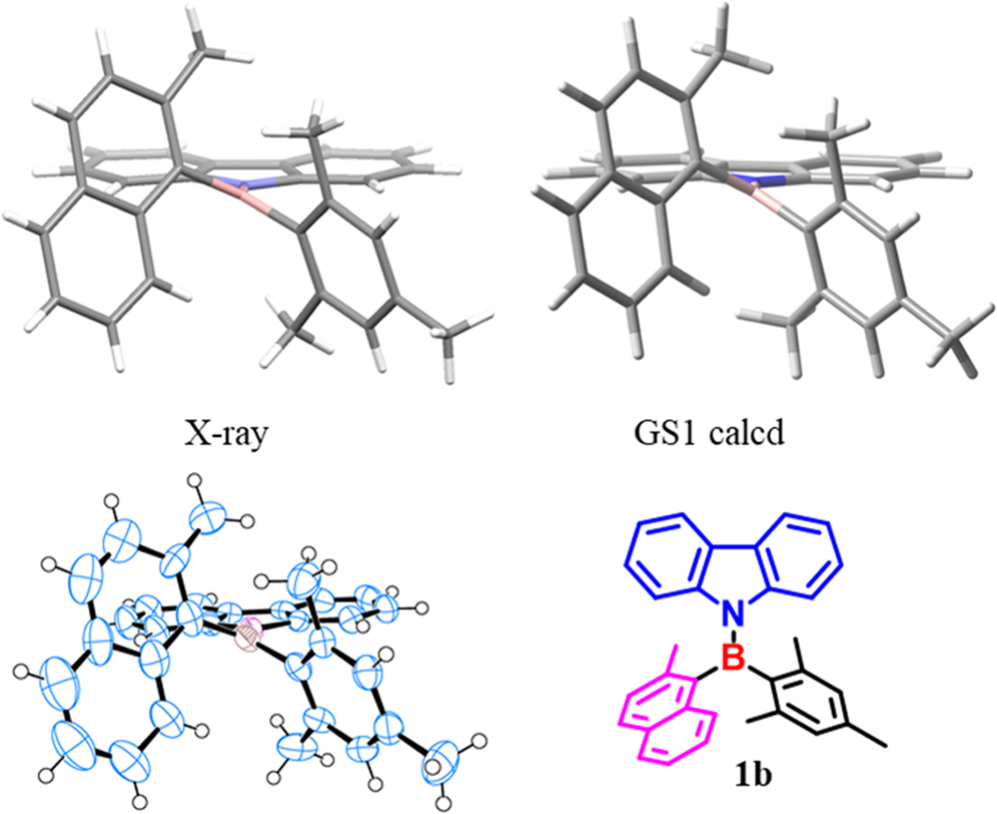
Perspective view derived from single-crystal X-ray analysis with a 50% ellipsoid probability and the best calculated structure for compound **1b**. The experimental B–N skew angle is 24.0°, and the calculated value for **GS1** was 28.2°.

Density functional theory (DFT) calculations were used to model and optimize the **GS** and **TS** structures^19^ using the B3LYP functional and the 6-311G(d,p) basis set. All of the optimized structures were validated by frequency analysis (see the Experimental Section for details). It was found that the two diastereomeric **GS1** and **GS2** conformations were similar in energy (1.12 vs 0.00 kcal/mol for **GS1** and **GS2** of **1a** and 0.43 vs 0.00 kcal/mol for **GS1** and **GS2** of **1b**, respectively). In both cases, carbazole was not perfectly coplanar with the C_q_-B-C_q_ plane to better arrange the substituents on boron (calculated skew angles: 33.2 and −24.2° for **GS1** and **GS2** of **1a**, 28.2 and −26.4° for **GS1** and **GS2** of **1b**). As in the case of the classical bis-mesityl aminoboranes, the 2-RF **TS** corresponding to the rotation of carbazole (conjugative barrier) is much higher than the 2-RF **TS** involving the rotation of the two aromatic rings on boron (21.5 vs 7.6 kcal/mol). Good-quality single crystals of **1b** were obtained by slow evaporation of a hexane solution, and the structure was solved in the monoclinic *P*21/*c* centrosymmetric space group (see Figures 3 and S13 in the Supporting Information for details). The agreement between the calculated and the experimental structures was very good. This is proof of the reliability of the employed DFT level.

**Figure 4:**
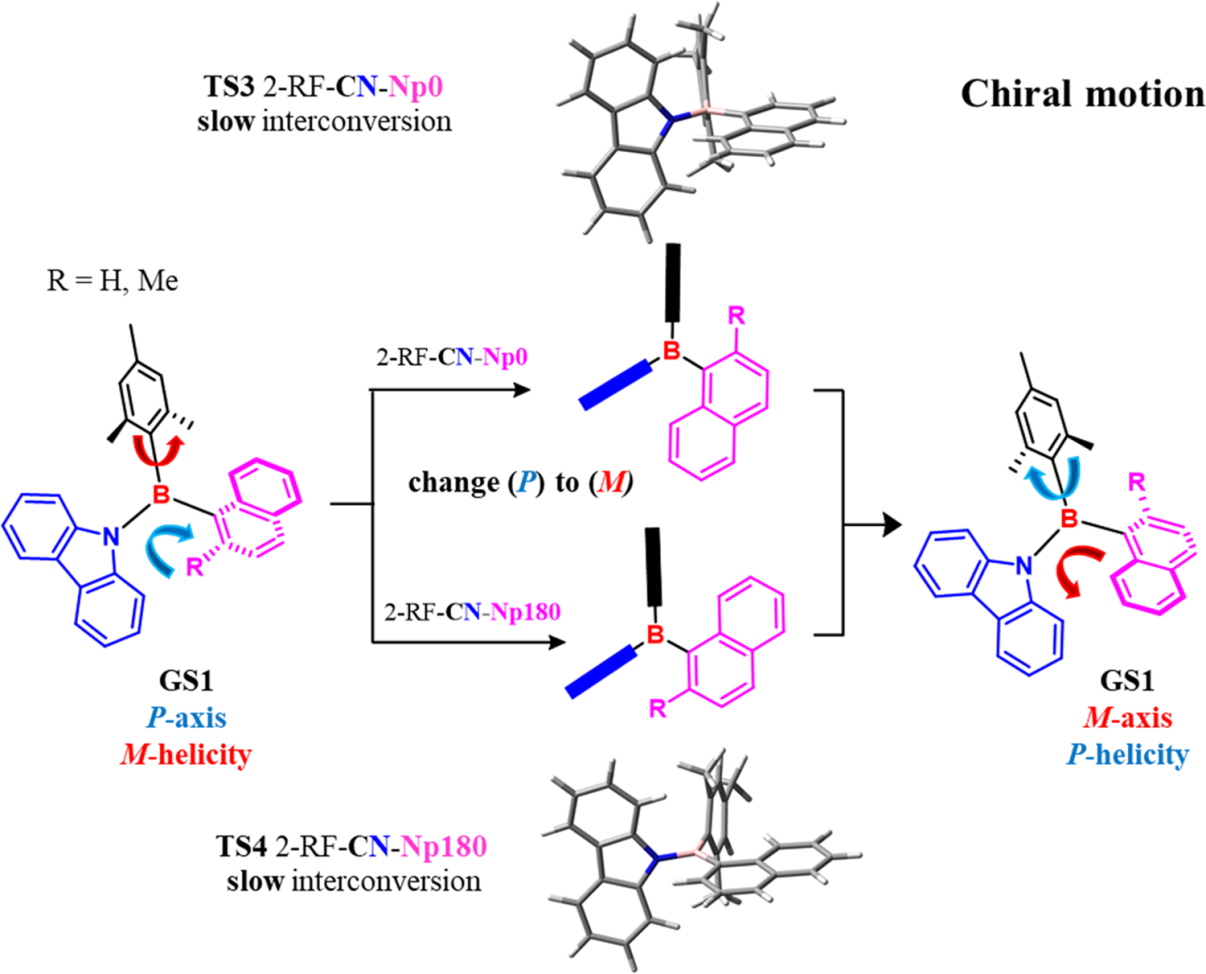
Scheme of available **GSs** and **TSs** for racemization of compounds **1a–b**.

However, the most interesting rearrangement for this class of compounds was the conformational pathways that linked the two atropisomers generated by the naphthyl-boron chiral axis. The corresponding **TS** had to consider naphthyl to be coplanar with the reference plane to change the axial chirality from *P* to *M* and *vice versa*. Two possible 2-RF **TSs** could be invoked, where the 2-substituent (H in **1a** or methyl in **1b**) of the naphthyl ring crossed the reference plane over mesityl (**TS3**, 2-RF-**CN-Np0** in Figure 4) or over carbazole (**TS4**, 2-RF-**CN-Np180** in Figure 4).

In the case of **1a**, the two rotational barriers were estimated to be 20.7 and 19.9 kcal/mol (**TS3** and **TS4**, respectively) by DFT, so the two atropisomers were not stable at ambient temperature. On the contrary, the higher steric hindrance of 2-methyl in **1b** increased the calculated values to 26.4 and 25.3 kcal/mol, respectively. Table 1 summarizes all of the results of DFT calculations, and the corresponding structures are reported in Figures S7–S9 in the Supporting Information.

**Table 1:** Calculated Gibbs Free Energy Barriers for the 2-RF Motion (Relative Energies in kcal/mol as Zero Point Energy-Corrected Enthalpies)

						TS3 2RF-CN-Ar0	
			TS1 2RF-CC-carb	TS2 2RF-CN-mes		TS4 2RF-CN-Ar180	
compd	GS1	GS2	calc.	calc.	exp.	calc.	exp.
**1a**	1.12	0.00	7.6	21.5		20.7	**20.3**^a^
						**19.9**	
**1b**	0.43	0.00	11.4	23.5	**23.9**^a^	26.4	**26.1**^b^
						**25.3**	
**1c**	0.00		9.7	**22.5**	**23.3**^a^	25.6^c^	

a Determined using 1D-EXSY NMR.

b Determined using standard kinetic analysis and CSP-HPLC.

c **TS3** and **TS4** are identical due to the *C*_2_ rotational symmetry of anthracene.

The experimental measurements of the predicted barriers were performed by NMR and chiral stationary-phase high-performance liquid chromatography (CSP-HPLC).

For compound **1c**, bearing symmetric anthracen-9-yl, the threshold conjugative energy barrier that exchanged the two sides of carbazole corresponded to **TS2**. The barrier was experimentally determined by one-dimensional NMR exchange spectroscopy (1D-EXSY) at 117.5 and 122.5 °C by irradiating the H-1 signal of carbazole and monitoring the increase in the corresponding exchange signal (H-8) at different mixing times. The experimental value of 23.3 kcal/mol was in fair agreement with the calculated value (further details are reported in Figure S12 in the Supporting Information).

In the cases of **1a** and **1b**, two different dynamic processes resulted in the effective rotation of carbazole. One of them (**TS2**) did not exchange the chiral axis, whereas **TS3** and **TS4** did exchange the chiral axis. Thus, NMR experiments could not determine which **TS** was responsible for carbazole rotation, while CSP-HPLC could selectively monitor the racemization through the **TS3**/**TS4** pathway.

In the case of **1a**, the **TS3**/**TS4** pathway had a calculated energy lower than that of **TS2**, so the threshold mechanism for carbazole rotation also implied racemization. However, the 19.9 kcal/mol calculated value was too low to allow for a physical observation of the enantiomeric pair with CSP-HPLC at ambient temperature. The experimental barrier for carbazole rotation was indeed monitored by 1D-EXSY NMR experiments in the 81.6–86.7 °C range, yielding an experimental value of 20.3 kcal/mol (Figure S10 in the Supporting Information).

Instead, in compound **1b**, **TS2** has a calculated energy lower than that of **TS3**/**TS4**, and the rotation of carbazole could be monitored by 1D-EXSY NMR experiments in the 122.5–127.6 °C range, which provided an experimental **TS2** value of 23.9 kcal/mol (Figure S11 in the Supporting Information).

**Figure 5:**
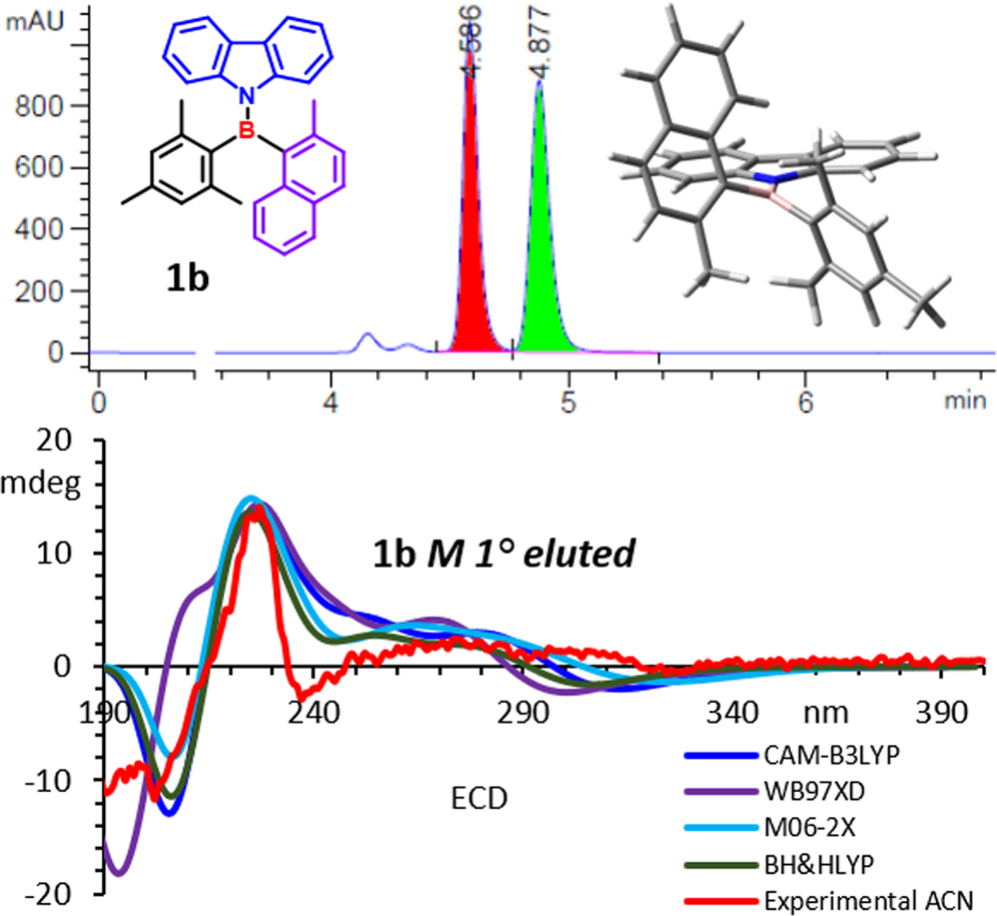
(Top) CSP-HPLC chromatogram of compound **1b** (CHIRALPAK AD–H 250 × 4.6 mm, *n*-HEX/*i*-PrOH 97:3; flow: 0.7 mL/min). (Bottom) Experimental and calculated ECD spectra of the first-eluted atropisomer using the reported functionals and the 6-311++G(2d,p) basis set.

Besides, compound **1b**, bearing the more hindered 2-methylnaphthyl, had a calculated energy barrier of 25.3 kcal/mol for TS4, which was high enough to allow the resolution of the atropisomers by CSP-HPLC. Resolution was effectively achieved on a CHIRALPAK AD-H column (Figure 5, top), and the electronic circular dichroism (ECD) spectra were recorded in acetonitrile (ACN; Figure 5, bottom) as well as other solvents (see the Supporting Information). The atropisomerization barrier of 26.1 kcal/mol was measured using an enantiopure sample in tetrachloroethane warmed at 50 °C, and the racemization was monitored with CSP-HPLC (see the Experimental Section and Figure S14 in the Supporting Information).

The absolute configuration of the atropisomers was determined by time-dependent DFT (TD-DFT) simulations of the ECD spectra.^20^ Being similar in energy, **GS1** (0.43 kcal/mol, 32.6%) and **GS2** (0.00 kcal/mol, 67.4%) conformations had to be considered, and the weighted sum of their simulated ECD spectra was applied (Figures 5 and S15 in the Supporting Information). All of the simulations assigned the *M* absolute configuration to the first-eluted atropisomer of **1b**.

## Absorption and Emission Properties

The absorption and emission properties of **1a–c** were studied at 25 °C in dilute solutions (10^–5^ M) using solvents with different polarities such as hexane (HEX), tetrahydrofuran (THF), dichloromethane (DCM), and ACN. The relevant data are listed in Table 2 and in the Supporting Information (Figures S16–S21).

**Table 2:** Relevant Absorption and Emission Data for Compounds **1a**, **1b**, and **1c**

	absorption	emission			
	λ (nm), 10^–4^ ε (cm^–1^ M^–1^)	λ_em_ (nm)	τ_ox_ (ns)	ϕ_ox_^a^ (%)	Stokes shift (cm^–1^)
**1a** HEX	218 (9.94), 282 (1.81), and 315 (1.18)	465	4	17	10,241
**1a** THF	221 (7.45), 284 (1.27), and 318 (0.79)	505	7	19	11,645
**1a** DCM	220 (8.12), 283 (1.38), and 316 (0.86)	509	8	29	11,999
**1a** ACN	222 (13.8), 256 (3.05), and 291 (3.83)	340, 354, and 520	3	26	
**1b** HEX	224 (9.20), 282 (1.40), 294 (0.99), and 320 (0.67)	448	2	6	8929
**1b** THF	223 (6.93), 281 (1.05), 290 (0.79), and 321 (0.51)	480	3	8	10,319
**1b** DCM	225 (7.45), 280 (1.16), 294 (0.74), and 321 (0.57)	485	3	9	10,534
**1b** ACN	225 (11.06), 282 (1.63), 292 (1.14), and 320 (0.74)	356 and 495	5	12	
**1c** HEX	263 (7.28), 287 (1.24), 377 (0.80), and 398 (0.82)	501	7	45	5166
**1c** THF	262 (5.60), 285 (1.05), 378 (0.73), and 398 (0.75)	525	4	22	6078
**1c** DCM	264 (6.92), 281 (1.03), 379 (0.59), and 400 (0.69)	535	4	57	6308
**1c** ACN	259 (7.46), 282 (1.11), 376 (0.67), and 397 (0.78)	340 and 564	2	15	

a vs quinine sulfate/0.05 M H_2_SO_4_, Φ = 0.5324.

The absorption profiles of **1a–c** exhibited intense π–π* transitions below ca. 300 nm, followed by weaker bands peaking at a lower energy that were assigned to charge transfer (CT) processes. The absorption spectra of the three different boranes were compared in the same solvents, showing how the absorption maxima shifted to a lower energy from the naphthyl-appended borane **1a** to the anthracenyl-substituted derivative **1c** (Table 2 and Figures S16–S21 in the Supporting Information). In this latter case, i.e., compound **1c**, the absorption profiles displayed well-resolved features peaking above λ = 350 nm, whose occurrence might be ascribed to CT processes involving the anthryl moiety. No appreciable solvatochromism was observed for compounds **1b** and **1c**, as the absorption profiles and the molar absorptivity of any transition did not vary in the presence of increasingly polar solvents. On the contrary, the absorption profile of compound **1a** in ACN solution displayed CT features that appeared less intense and blueshifted with respect to those recorded in HEX, THF, and DCM solutions.

Upon excitation (λ_exc_ = 310 nm) of the corresponding dilute solutions (10^–5^ M), the chiral bis-aryls carbazole boranes **1a–c** displayed luminescence, with emission maxima spanning from 448 nm (for **1b** dissolved in HEX) to 535 nm (for **1c** dissolved in DCM). In all cases, the time-resolved analyses of the radiative processes suggested lifetime values (τ) congruent with the fluorescent nature of the emissions. Although the emission intensities of **1a–c** were found to be quite weak, compounds **1b** (Ar = 2-methyl-naphthyl) and **1c** (Ar = anthracen-9-yl) displayed broad and structureless emission profiles that gradually shifted to a lower energy with increasing solvent polarity, i.e., from HEX to THF to DCM. The occurrence of a similar solvatochromic effect suggested that CT transitions make a significant contribution to the nature of the emissive excited states. In this regard, a deeper investigation was required for the discussion of the emissive behavior of the boranes **1a–c** when dissolved in ACN, i.e., the most polar medium in the series of solvents employed. In fact, under these conditions (10^–5^ M ACN solutions at r.t.; Table 2), along with the expected broad, structureless, and red-shifted process peaking in between λ_max_ of ca. 495 nm (**1b**) and ca. 564 nm (as for compound **1c**), one additional feature occurring at a higher energy (Table 2 and Figures S17–S21 in the Supporting Information) was clearly detected for compounds **1a** (λ_max_ ca. 350 nm) and **1b** (λ_max_ ca. 355 nm) while being barely observable for **1c** (λ_max_ ca. 350 nm). In its entirety, the occurrence of this feature was rationalized using DFT and TD-DFT calculations (discussed later), whose output suggested the existence of two plausible **GS** conformations (**GS1** and **GS2**; Table S2 in the Supporting Information) for compounds **1a** and **1b**, while a single and prevalent **GS** conformation (**GS1**; Table S2 in the Supporting Information) was calculated for compound **1c**. Furthermore, in agreement with a previous report by Kang and co-workers on similar D–A systems,^21^ the occurrence of the higher-energy features in the luminescence output of the ACN solutions of compounds **1a–c** was most likely due to carbazole-based processes. In particular, the selective observation of both higher- and lower-energy features only in spectra of compounds **1a–c** recorded in ACN solutions could be explained by the efficient stabilization of the CT states by a highly polar solvent such as ACN, highlighting an effect that we described in our recent report on similar systems.^1a^

The highest values of fluorescence quantum yields (Φ) (Table 2) were measured for compound **1c** (15–57% vs quinine sulfate in 0.05 M H_2_SO_4_), while the lowest Φ was measured for **1b**. In contrast, the smallest Stokes shift was measured for **1c** (from 5166 cm^–1^ in HEX to 6300 cm^–1^ DCM), while much higher values were measured for **1a** and **1b**, both exceeding 10,000 cm^–1^ in THF and DCM.

Overall, the coexistence of a large Stokes shift and low quantum yield confirmed that a rearrangement of the N–B bond takes place upon excitation (TICT behavior), while high Φ values and smaller Stokes shifts generally account for less-twisted molecules and poor TICT contributions.^22^

The whole fluorescence cycle was simulated by TD-DFT calculations using the CAM-B3LYP functional,^23^ and the solvent effects were considered using the IEF-PCM model.^1a,24^

In Table S2 in the Supporting Information, the calculated values of emission wavelengths, absorption wavelengths, and Stokes shift are reported for compounds **1a–c** in four different solvents (HEX, DCM, THF, and ACN). For all of the compounds, a positive solvatochromic effect in emission was confirmed by calculations, which is well explained by the TICT character of the excited states (Figure S22 in the Supporting Information). The more polar the solvent, the more effective the nonradiative stabilization by B–N rotation in the excited state. Analysis of the shapes of the HOMO and LUMO, involved in the vertical transition emission, highlighted that they were localized in two different regions of the molecules: the carbazole side (donor group) and bis-aryl-boron side (acceptor group), respectively. In compound **1b** (Figure S22), the naphthyl moiety was the preferred acceptor with respect to the mesityl moiety, both in the absorption and emission transitions. The Δϕ angle around B–N due to the TICT rearrangement was 29° (as the average of **GS1** and **GS2** optimized in the excited state). Compound **1b** in ACN had a calculated emission band at 457 nm, in accordance with the experimental band observed at 495 nm (see experimental part).

Therefore, we tested the stability of compound **1b** by monitoring the ^1^H NMR spectrum in deuterated ACN. ^1^H NMR spectra were collected every 4 h (Figure S23). As shown in the aliphatic region, a slow degradation of compound **1b** into byproducts was observed, which had a very similar absorption spectrum but a different emission behavior.

## CPL Properties

The chirality and the high luminescence of compound **1b** prompted us to record its CPL spectra. The spectra were run in three different solvents with increasing polarity (HEX, DCM, and ACN) in ∼1 × 10^–4^ M solutions. As a comparison, the ECD spectra of the same solutions were also recorded (see details in the Supporting Information).

**Figure 6:**
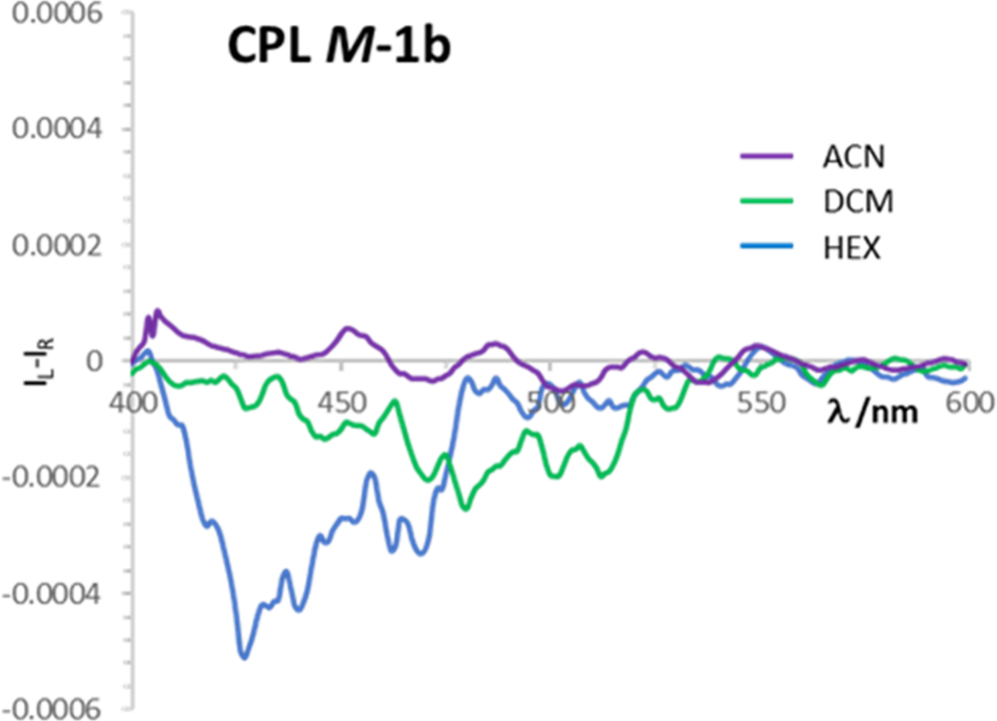
Comparison of CPL spectra of ***M*****-1b** in HEX, DCM, and ACN.

A weak CPL band was obtained in HEX, showing a positive sign for ***P*****-1b** and a negative one for ***M*****-1b** (Figure S24, top). The dissymmetry factors (*g*_lum_), estimated as the integral of each CPL spectrum divided by the integral of the respective total fluorescence, were approximately ±3 × 10^–4^. The ECD spectrum in this solvent was more intense than that in the other two solvents (Figure S24). The samples measured in DCM solution displayed weaker CPL signals (*g*_lum_ around ±2 × 10^–4^), but with the same signs observed in HEX (Figure S25, top). Regarding the more polar ACN, no significant CPL was recorded, thus confirming the trend. The major role of the nature and polarity of the solvent in chiroptical activity, and CPL in particular, was expected especially in the case of relatively flexible molecules.^25^ The comparison of the CPL spectra of **M-1b** in the three solvents is shown in Figure 6.

The *g*_lum_ factors were approximately 3/4 times lower than the g_abs_ values observed for the most red-shifted Cotton effect in the CD spectrum at 325 nm, ranging from 6.0 × 10^–4^ in ACN to 8.5 × 10^–4^ in HEX (Figure S26, bottom). The difference between *g*_lum_ and *g*_abs_ confirmed some significant modifications in the geometry of the ground state and the emitting excited states. This is in contrast to what was observed for most rigid organic compounds, where a stronger correspondence was found.^26^ In contrast, the relation between *g*_lum_ and *g*_abs_ in the three different solvents was roughly linear (Figure S27). This suggested a similar polarity effect on the ground- and excited-state geometry of compound **1b**. The observation of CPL activity from carbazole boranes reported herein complements prior studies reporting CPL activity from other B-based organic compounds, such as BODIPYs,^27^ B-containing helicenes,^28^ diboryl-binaphthyls,^29^ and organoboranes integrated in pillar[5]arenes frameworks.^25b^

## Conclusions

We have shown that a simple modification to a known reaction pathway for the preparation of bis-aryl aminoboranes can effectively yield aminoboranes with different aryl rings on the boron branch. The chemical approach for the preparation of the key intermediate relies on the activation of mesityl trifluoroborate with a Grignard reagent of the second aryl ring. This allowed the use of a stable and easy-to-handle boron source and allowed for the preparation of a variety of dissymmetric bis-aryl aminoboranes.

The stereodynamic behavior was analyzed via NMR spectroscopy, CSP-HLPC, and DFT calculations to investigate the threshold **TS** geometries responsible for the observed energy barriers. The use of aryl rings, such as 1-naphthyl and 2-methylnaphthyl, allowed the preparation of bis-aryl aminoboranes bearing a B–C chiral axis, whose enantiomers could be separated by CSP-HPLC in the case of compound **1b**. The emissive properties were investigated in different solvents, showing solvatochromism in the emission spectra owing to the TICT process, with very large Stokes shifts (>10,000 cm^–1^ for **1a** and **1b**). CPL spectra were acquired in the case of the stable atropisomers of **1b**, showing the maximum intensity in apolar solvents. Compound **1b** can be considered a new example of a CPL-active bis-aryl aminoborane with an exocyclic B–N bond.

## Experimental Section

### NMR

NMR spectra were recorded using a spectrometer operating in a field of 14.4 T (600 MHz for ^1^H, 151 MHz for ^13^C, 192 MHz for ^11^B, and 376 MHz for ^19^F). Chemical shifts are given in parts per million relative to the internal standard tetramethylsilane (^1^H and ^13^C) or relative to the residual peak of the solvents. The 151 MHz ^13^C spectra were acquired under proton decoupling conditions with a 36,000 Hz spectral width, 5.5 μs (60° tip angle) pulse width, 1 s acquisition time, and a 5 s delay time. The ^13^C signals were assigned by distortionless enhancement by polarization transfer spectra.

### X-ray Crystallographic Study

Crystal data and collection details for **1b** are reported in Figure 3 and in the Supporting Information. The diffraction experiments were carried out using a Bruker Apex II diffractometer equipped with a PHOTON2 detector using Mo Kα radiation. The data were corrected for Lorentz polarization and absorption effects (empirical absorption correction SADABS).^30^ Structures were solved by direct methods and refined by full-matrix least-squares based on all data using F^2^.^31^ Hydrogen atoms were fixed at calculated positions and refined with a riding model. All nonhydrogen atoms were refined with anisotropic displacement parameters unless otherwise stated.

### High-Resolution Mass Spectra

High-resolution mass spectra (HRMS) were recorded using a Waters TOF Premier spectrometer.

### Photophysical Measurements, Calculation, ECD Spectra, and CPL Spectra

Photophysical measurements, calculations, ECD spectra, and CPL spectra are described in detail in the Supporting Information.

### Racemization Rate Measurements

An aliquot of a pure atropisomer of **1b** was dissolved in 1 mL of C_2_D_2_Cl_4_ in a two-neck balloon: one neck with a septum for sampling and one neck for inserting a thermometer. The balloon was kept at 50 °C. C_2_D_2_Cl_4_ was chosen because of its high boiling point and good vapor pressure, which allow it to easily evaporate. Small aliquots were taken at different times, the solvent was evaporated, and the sample was analyzed by enantioselective HPLC, which allowed the determination of the enantiomeric ratio at different reaction times. A first-order kinetic equation was then used to derive the rate constant for racemization and, hence, the activation barrier using the Eyring equation.

## Materials

Analytical-grade solvents and commercially available reagents were used as received. THF and Et_2_O were dried by distillation over Na/benzophenone before use. Toluene was distilled under argon before use. The following stationary phases were employed for the chromatography: silica gel 60 Å F254 (Merck) for TLC and silica gel 60 Å (230–400 mesh, Sigma-Aldrich) for atmospheric pressure chromatography. Reactions were performed under a dry argon flow. The glassware used in these reactions was placed in an oven at 70 °C for at least 3 h immediately before use. Naphthyl-BF_3_K and mesityl-BF_3_K were synthesized using a previously reported procedure^8^ starting with commercially available aryl boronic acids.

### Naphthyl-BF_3_K (166328-07-0)

^1^H NMR (400 MHz, acetonitrile-d_3_) δ 8.51–8.43 (m, 1H), 7.81–7.73 (m, 1H), 7.70–7.62 (m, 3H), and 7.44–7.32 (m, 5H).

^19^F NMR (376 MHz, acetonitrile-d_3_) δ −137.91, −138.04, −138.19, and −138.32 (B–F quartet).

### Mesityl-BF_3_K (244301-57-3)

^1^H NMR (400 MHz, acetonitrile-d3) δ 6.63 (bs, 2H), 2.35 (q, JH-F = 1.9 Hz, 6H), 2.18 (s, 3H).

^19^F NMR (376 MHz, acetonitrile-d3) δ −131.36, −131.48, −131.64, and −131.78 (B–F quartet).

### General Procedure for the Synthesis of Compounds **1a–c**

In a 25 mL oven-dried reaction flask, magnesium turnings (10 equiv), toluene (10 mL), Et_2_O (10 mL), and a spatula tip of iodine were added. Aryl bromine (2.4 mmol) was dropped at 25 °C, and the resulting solution was stirred under argon at reflux using a heating mantle. The reaction time was 1 h when the iodine discoloration was visible. After cooling to room temperature, MesBF_3_K (2.4 mmol = 0.543 g) was added, and the reaction was stirred for another 1 h. The formation of the mes-aryl fluoroborate was checked by ^19^F NMR. In the meantime, in another 25 mL oven-dried reaction flask, carbazole (2.4 mmol = 0.401 g) in THF (10 mL) was reacted with KHMDS (2.4 mmol, 0.5 M in toluene) at room temperature. After 1 h, when MesBF_3_K disappeared, mes-aryl fluoroborate solution was dropped to the second flask at room temperature. The residue was diluted in DCM and filtered on Celite, and then, the solvent was evaporated. The products were purified by chromatographic separation on a silica gel with a 9:1 *n*-hexane/DCM eluent.

#### 9-(Mesityl(naphthalen-1-yl)boraneyl)-9H-carbazole

Compound **1a** was synthesized starting with naphthyl-BF_3_K (2.4 mmol = 0.562 g) and mesityl bromine (2.4 mmol = 0.478 g). The products were purified by chromatography separation on silica gel with a 9:1 *n*-hexane/DCM eluent. Compound **1a** (amorphous white solid, 0.61 g = 1.44 mmol) had a yield of 60%.

^1^H NMR (600 MHz, CDCl_3_) δ 8.00 (dd, *J* = 7.7, 1.4 Hz, 1H), 7.98–7.92 (m, 2H), 7.87 (d, *J* = 8.4 Hz, 1H), 7.78 (d, *J* = 8.5 Hz, 1H), 7.63 (dd, *J* = 6.9, 1.4 Hz, 1H), 7.51 (dd, *J* = 8.2, 6.8 Hz, 1H), 7.39 (ddd, *J* = 8.2, 6.9, 1.3 Hz, 1H), 7.30 (t, *J* = 7.5 Hz, 1H), 7.19–7.11 (m, 3H), 6.99 (d, *J* = 8.4 Hz, 1H), 6.89 (s, 1H), 6.88 (s, 1H), 6.77 (ddd, *J* = 8.5, 7.2, 1.4 Hz, 1H), 6.46 (d, *J* = 8.5 Hz, 1H), 2.36 (s, 3H), 2.08 (s, 3H), 2.07 (s, 3H).

^13^C{^1^H} NMR (151 MHz, CDCl_3_, 77 ppm) δ 144.1 (Cq), 142.7 (Cq), 140.6 (Cq-B broaden), 140.1 (Cq), 138.7 (Cq), 138.3 (Cq), 134.9 (Cq), 133.2 (CH), 133.0 (Cq), 130.6 (CH), 128.6 (CH), 128.4 (Cq), 128.4 (CH), 128.2 (CH), 128.0 (CH), 126.6 (CH), 126.4 (CH), 125.8 (CH), 125.7 (CH), 125.6 (CH), 122.9 (CH), 122.7 (CH), 119.5 (CH), 119.3 (CH), 117.0 (CH), 115.7 (CH), 22.6 (CH_3_), 21.8 (CH_3_), 21.4 (CH_3_).

^11^B NMR (192 MHz, CDCl_3_) δ 51.62.

HRMS (ESI-QTOF). Calculated for C_31_H_26_BNNa^+^ [M + Na]^+^, 446.2056; found, 446.2065.

#### 9-(Mesityl(2-methylnaphthalen-1-yl)boraneyl)-9H-carbazole

Compound **1b** was synthesized using 1-bromo-2-methyl-naphthalene (2.4 mmol = 0.531 g) as the starting material. The products were purified by chromatographic separation on a silica gel with a 9:1 *n*-hexane/DCM eluent. Compound **1b** (crystalline white solid, 0.766 g = 1.75 mmol) was obtained with a yield of 73%.

Atropisomers were separated using a CHIRALPAK AD-H column (250 × 21.2 mm, 20 mL/min) with *n*-hexane:*i*-PrOH (97:3) as the eluent.

^1^H NMR (600 MHz, methylene chloride-d_2_, 5.32 ppm) δ 8.02 (dt, *J* = 7.8, 1.0 Hz, 1H), 7.97 (dt, *J* = 7.7, 1.1 Hz, 1H), 7.89 (d, *J* = 8.4 Hz, 1H), 7.86–7.81 (m, 1H), 7.71 (dd, *J* = 8.5, 1.1 Hz, 1H), 7.37–7.28 (m, 3H), 7.20–7.10 (m, 3H), 7.03 (dt, *J* = 8.5, 0.9 Hz, 1H), 6.89 (s, 1H), 6.88–6.82 (m, 2H), 6.65–6.60 (m, 1H), 2.34 (s, 3H), 2.27 (s, 3H), 2.04 (s, 3H), 2.00 (s, 3H).

^13^C{^1^H} NMR (151 MHz, methylene chloride-d_2_, 53.3 ppm) δ 143.2 (Cq), 142.8 (Cq), 140.9 (Cq-B broaden), 140.8 (Cq-B broad), 139.9 (Cq), 139.6 (Cq), 138.7 (Cq), 138.4 (Cq), 135.5 (Cq), 131.7 (Cq), 129.8 (CH), 129.3 (CH), 128.8 (CH), 128.7 (CH), 128.4 (CH), 128.2 (Cq), 128.1 (Cq), 127.2 (CH), 126.2 (CH), 125.9 (CH), 125.8 (CH), 124.6 (CH), 122.8 (CH), 122.6 (CH), 119.4 (CH), 119.3 (CH), 115.7 (CH), 115.5 (CH), 22.4 (CH_3_), 21.8 (CH_3_), 21.4 (CH_3_), 20.9 (CH_3_).

^11^B NMR (192 MHz, methylene chloride-d_2_) δ 53.77.

HRMS (ESI-QTOF). Calculated for C_32_H_28_BNNa^+^ [M + Na]^+^, 460.2212; found, 460.2201.

#### 9-(Anthracen-9-yl(mesityl)boraneyl)-9H-carbazole

Compound **1c** was synthesized using 9-bromo-anthracene (2.4 mmol = 0.617 g) as the starting material. The products were purified by chromatographic separation on a silica gel with a 9:1 *n*-hexane/DCM eluent. Compound **1c** (amorphous white solid, 0.42 g = 0.89 mmol) was obtained with a yield of 37%.

^1^H NMR (600 MHz, CDCl_3_) δ 8.56 (s, 1H), 8.01 (dt, *J* = 7.9, 1.9 Hz, 3H), 7.95 (dd, *J* = 8.8, 1.0 Hz, 2H), 7.89 (dt, *J* = 7.6, 0.9 Hz, 1H), 7.38–7.30 (m, 3H), 7.16 (dddd, *J* = 7.8, 6.4, 3.8, 1.3 Hz, 3H), 7.10–7.02 (m, 2H), 6.83 (s, 2H), 6.60 (ddd, J = 8.6, 7.2, 1.3 Hz, 1H), 6.19–6.15 (m, 1H), 2.31 (s, 3H), 2.00 (s, 6H).

^13^C{^1^H} NMR (151 MHz, CDCl_3_) δ 143.5 (Cq), 142.7 (Cq), 140.9 (Cq), 139.5 (Cq), 138.6 (Cq-B broaden), 134.6 (Cq), 131.3 (Cq), 129.8 (CH), 129.1 (CH), 129.1 (CH), 128.6 (Cq), 128.2 (Cq), 127.9 (CH), 126.4 (CH), 126.1 (CH), 125.9 (CH), 125.0 (CH), 123.1 (CH), 122.6 (CH), 119.6 (CH), 119.3 (CH), 116.4 (CH), 115.9 (CH), 22.6 (CH_3_), 21.3 (CH_3_).

^11^B NMR (192 MHz, CDCl_3_) δ 55.05.

HRMS (ESI-QTOF). Calcd for C_35_H_28_BNNa^+^ [M + Na]^+^, 496.2207; found, 496.2219.

## Supporting Information Available

Experimental details, characterization data, NMR spectra, X-ray, DFT calculations, photophysical spectra, ECD, and CPL spectra (PDF)
