# Supplementary material for: Synthesis and Stereodynamic and Emission Properties of Dissymmetric Bis-Aryl Carbazole Boranes and Identification of a CPL-Active B–C Atropisomeric Compound
Source: J Org Chem. 2023 Jan 4;88(2):871–81. doi: 10.1021/acs.joc.2c02209 (PMC9872089; doi:10.1021/acs.joc.2c02209)
Supplement: Supplementary file 1 — jo2c02209_si_001.pdf [file jo2c02209_si_001.pdf]

Synthesis, stereodynamic and emission properties of dissymmetric bis-aryl carbazole boranes. Identification of a CPL-active B-C Atropisomeric compound.

Daniel Pecorari,[a] Emanuele Giuliani,[a] Andrea Mazzanti,[a] Stefano Stagni,[a] Valentina Fiorini,[a] Giulia Vigarani,[a] Francesco Zinna,[b] Gennaro Pescitelli,[b] and Michele Mancinelli.[a,*]

a University of Bologna, Department of Industrial Chemistry "Toso Montanari" Viale del Risorgimento 4, 40136, Bologna, Italy.

b University of Pisa, Department of Chemistry and Industrial Chemistry, Via Moruzzi 13, 56124, Pisa, Italy.

e-mail: michele.mancinelli@unibo.it

Supporting information

Table of contents

One-Step Synthesis **S2**

Table S1: **S2**

Figure S1: **S3**

Figures S2-S6: NMR Spectra **S4**

Figures S7-S9: GSs and TSs 3D-structures **S8**

Figures S10-S12: EXSY experiment **S11**

Figures S13: X-Ray 1b structures **S14**

Figures S14: Kinetic of the racemization of 1b **S15**

ECD and Fig. S15: **S16**

Figures S16-S21: Photophysical Measurements **S17**

DFT and TD-DFT calculations of Photophysical properties **S21**

Table S2: calculated of Photophysical properties **S25**

Figure S22: HOMO-LUMO **S26**

Figure S23: degradation of 1b in ACN S27

Circularly polarized luminescence measurements: S28

Figures S24-S26: CPL S29

Crystal Data for compound 1b S33

DFT calculated geometries for all compounds: S42

One-Step synthesis for the synthesis of intermediate **I1b**

In a 25 mL oven-dried reaction flask, magnesium turnings (10 eq), a tip of iodine, $MesBF_3K$ (2.4 mmol) and the solvents Toluene (10 mL) and Et_2O (10 mL) were added. The arylbromine (2.4 mmol) was dropped at room temperature and the resulting solution was stirred under argon at reflux. When the discoloration of iodine was visible the reaction time was taken for 1 h. After cooling to room temperature, the formation of the Mes-Aryl fluoro borate was checked at ^{19}F NMR. Meanwhile, in another 25 mL oven-dried reaction flask, carbazole (2.4 mmol) in THF (10 mL) was reacted with KHMDS (2.4 mmol) at room temperature. After 1-hour, when $MesBF_3K$ salt has been disappeared, the Mes-Aryl fluoro borate solution was dropped to the second flask at room temperature. The residue was diluted in DCM and filtered on Celite®, then evaporated the solvent. The products were purified by chromatography separation on silica gel, with eluent *n*-hexane/DCM in 9:1 ratio.

Table S1. Optimization steps for the preparation of **1b** (Aryl = 2-methyl-naphthyl):

$MesBF_3K$ Activation Method	1° Step				2° Step			Product (% Yield)
	Solvent	Temp.	**I1b**	**By-products[a]**	Basis	Solv.	Temp.	**1b**
**BF_3Et_2O**[1a]	DCM	-40 °C to -78 °C	-	-	KHMDS	THF	r.t.	10
**ArylMgBr** (one step)	Et_2O/Toluene 1:1 v/v	reflux	-	-	NaH	THF	r.t.	20
	Et_2O/Toluene 1:1 v/v	reflux	-	-	BuLi	THF	r.t.	18
	Et_2O/Toluene 1:1 v/v	reflux	27	73	KHMDS	THF	r.t.	19

**ArylMgBr** (two step)	Et_2O/Toluene 1:1 v/v	r.t.	94	6	KHMDS	THF	r.t.	73

a. The sum by-products yields are derived from the ^{19}F NMR of Figure S1.

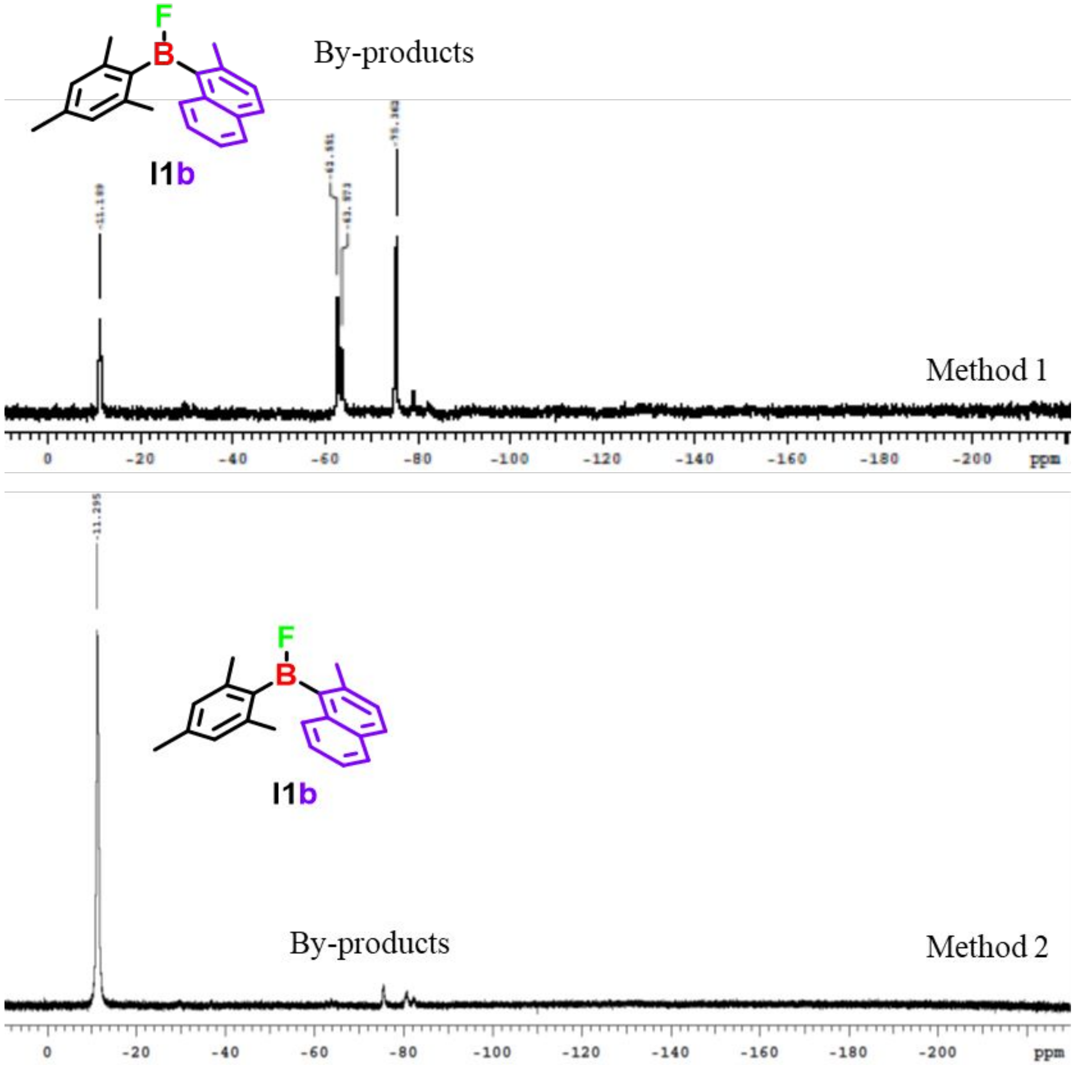

Figure S1. ^{19}F NMR (376.3 MHz) of Intermediate **I1b** prepared with **one-step (top)** and with **two-step (bottom)**.

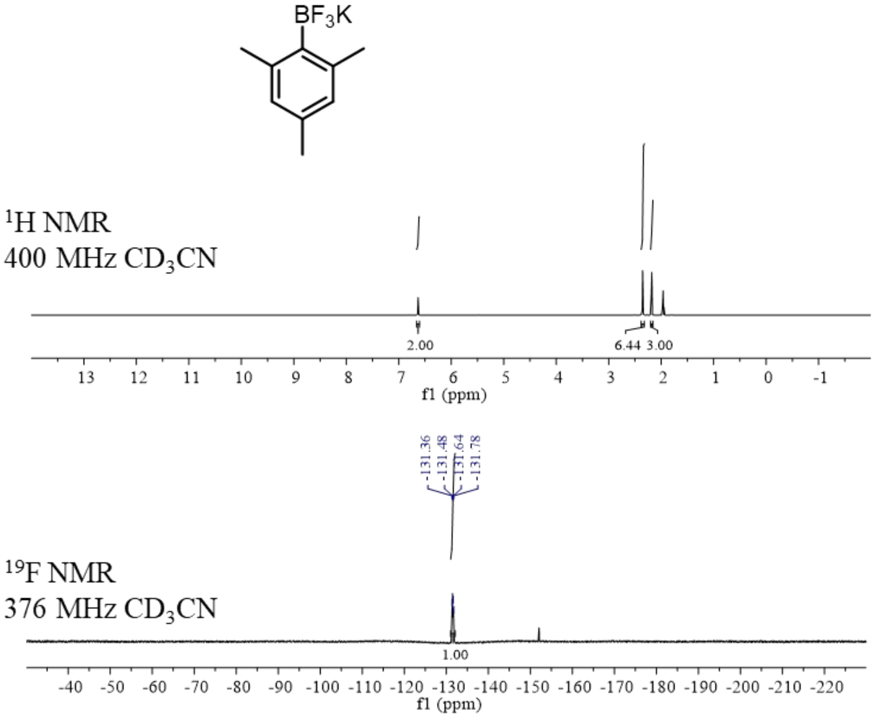

^{1}H NMR (400 MHz, Acetonitrile-d_3) δ 6.63 (bs, 2H), 2.35 (q, J_{H-B} = 1.9 Hz, 6H), 2.18 (s, 3H).

^{19}F NMR (376 MHz, Acetonitrile-d_3) δ -131.36, -131.48, -131.64, -131.78 (B-F quartet).

Figure S2. NMR spectra of mesityl-BF_3K

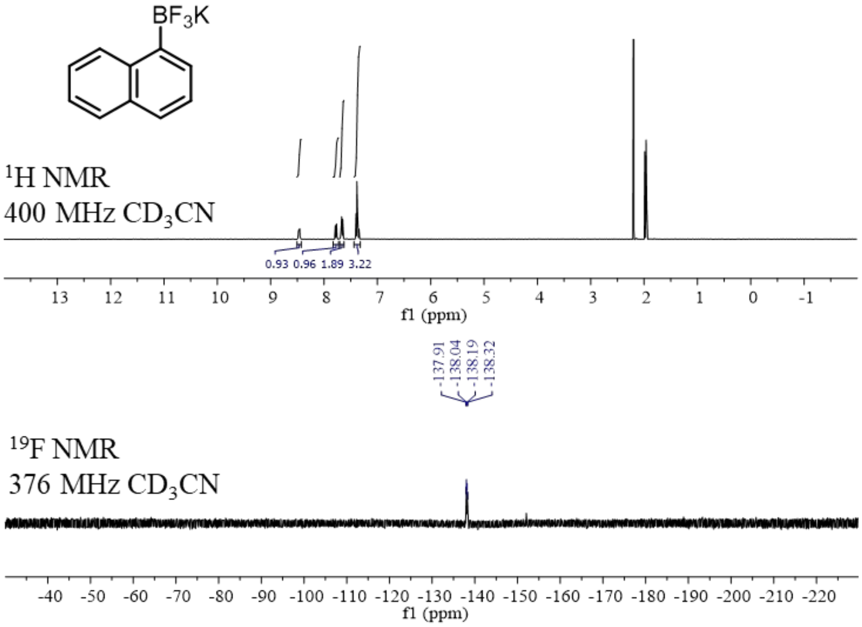

^{1}H NMR (400 MHz, Acetonitrile-d_3) δ 8.51 – 8.43 (m, 1H), 7.81 – 7.73 (m, 1H), 7.70 – 7.62 (m, 3H), 7.44 – 7.32 (m, 5H).

^{19}F NMR (376 MHz, Acetonitrile-d_3) δ -137.91, -138.04, -138.19, -138.32 (B-F quartet).

Figure S3. NMR spectra of naphthyl-BF3K

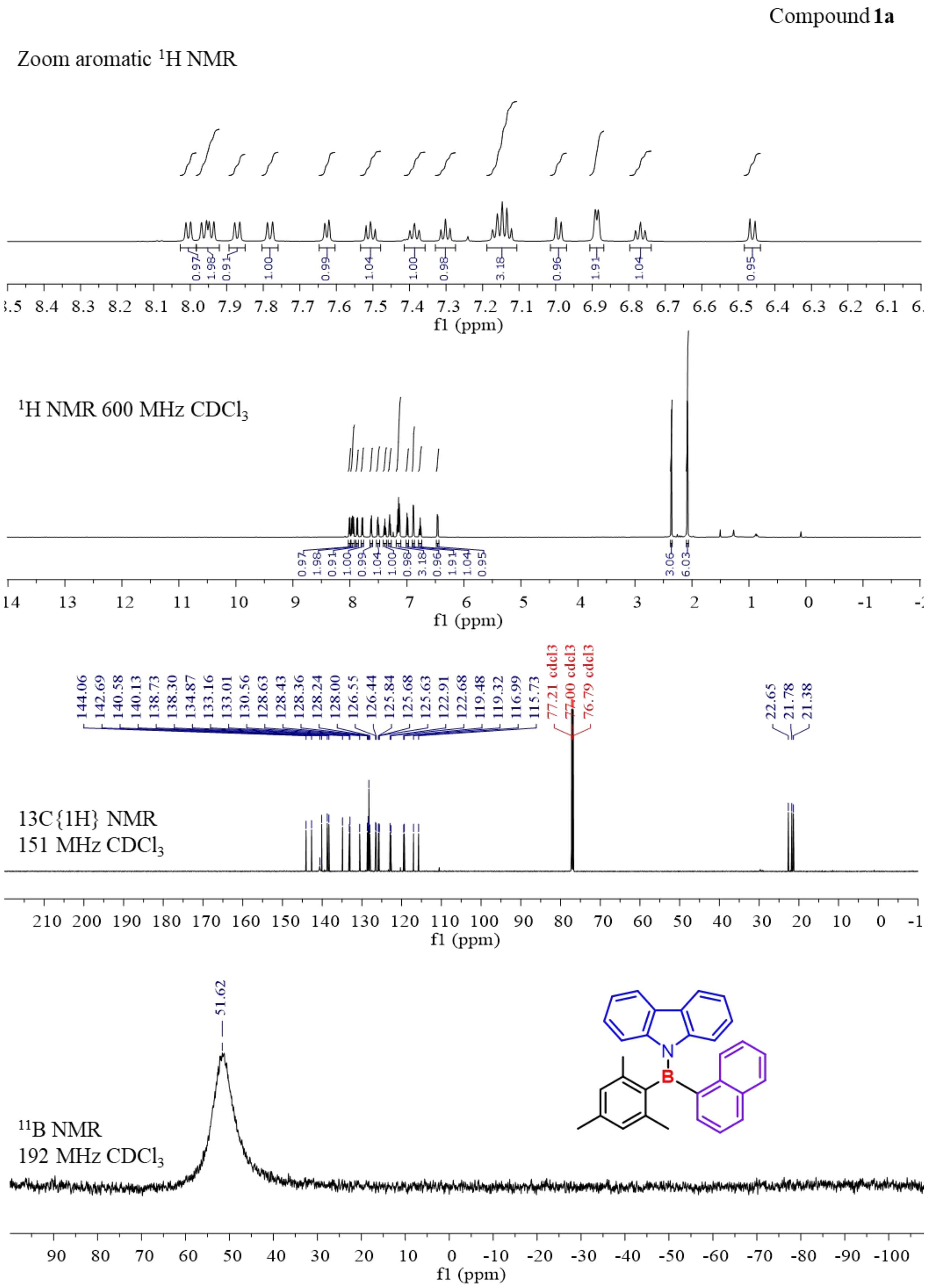

Figure S4. NMR spectra of compound **1a**.

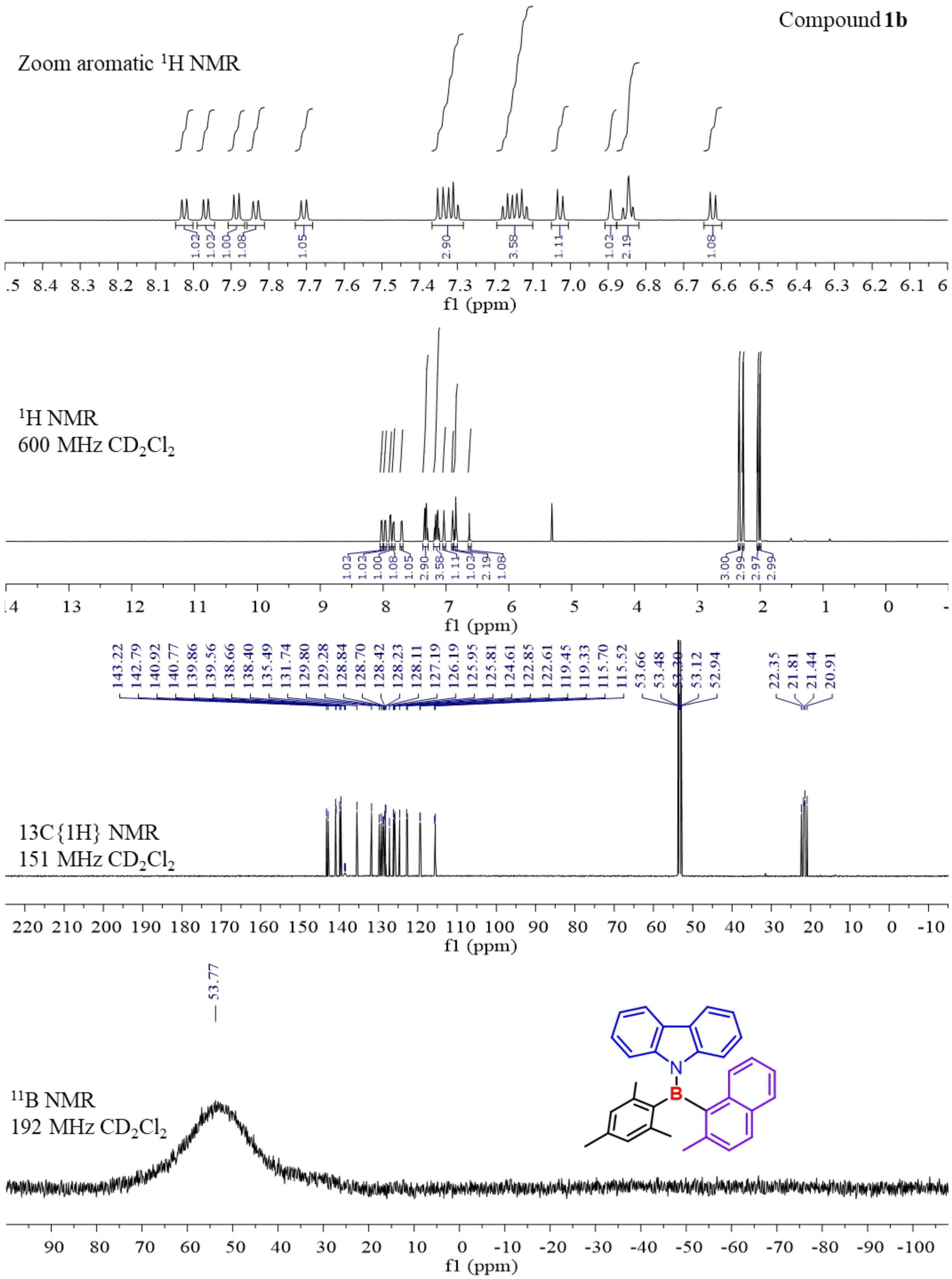

Figure S5. NMR spectra of compound **1b**.

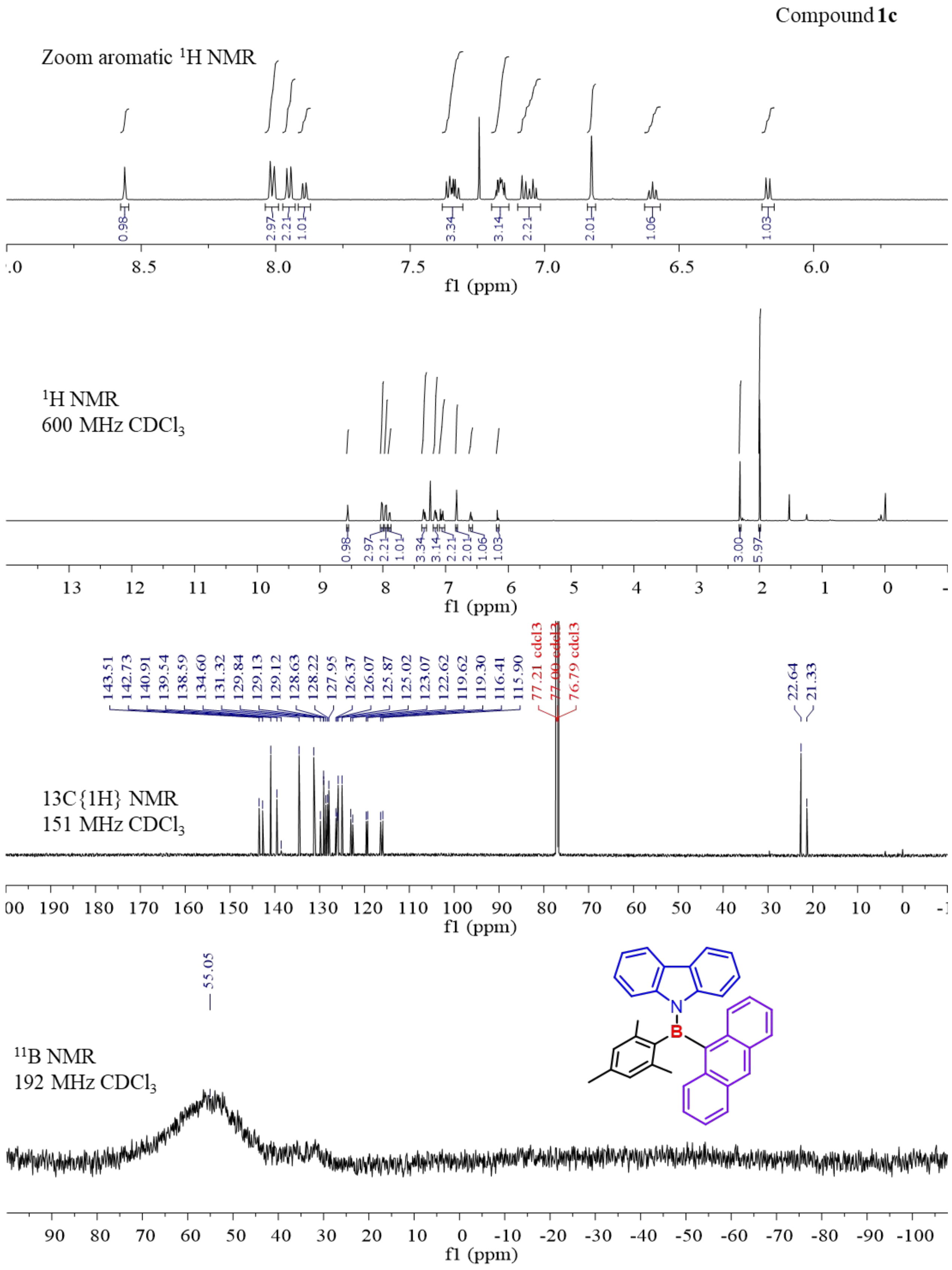

Figure S6. NMR spectra of compound **1c**.

Compound 1a

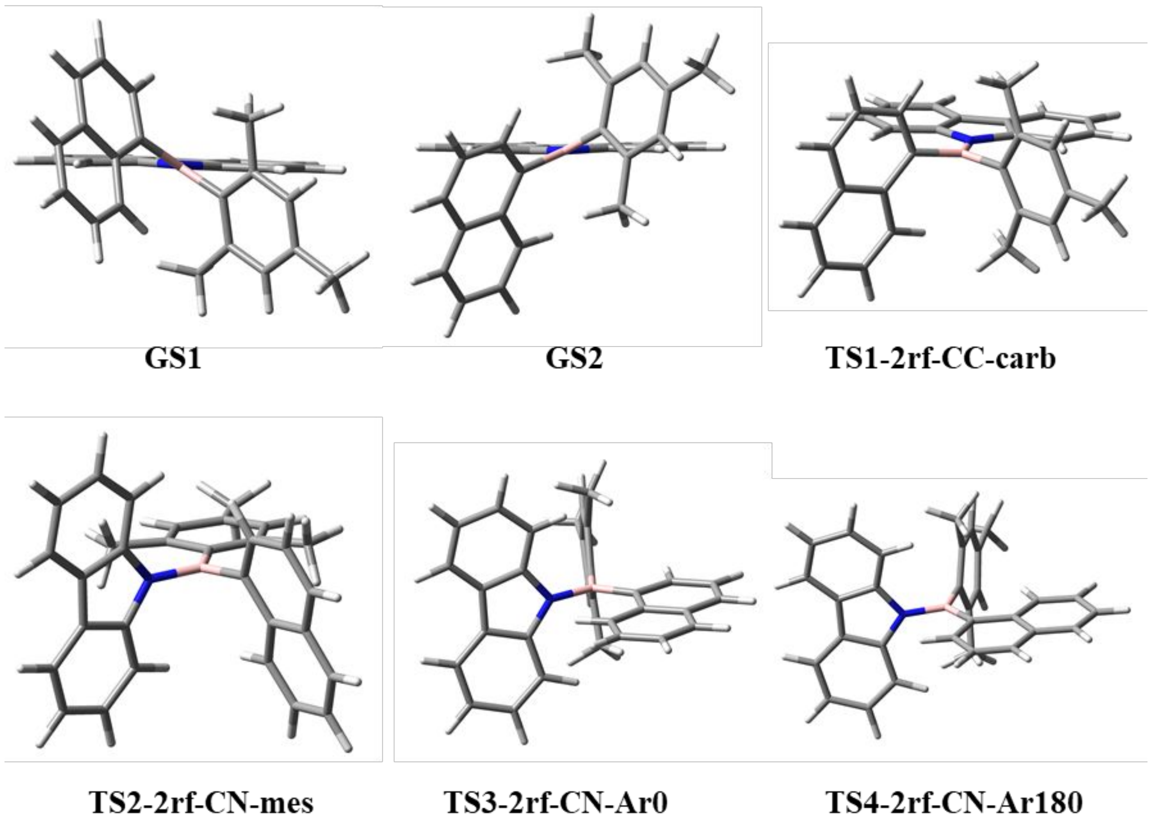

Figure S7. Calculated GSs and TSs for compound **1a**.

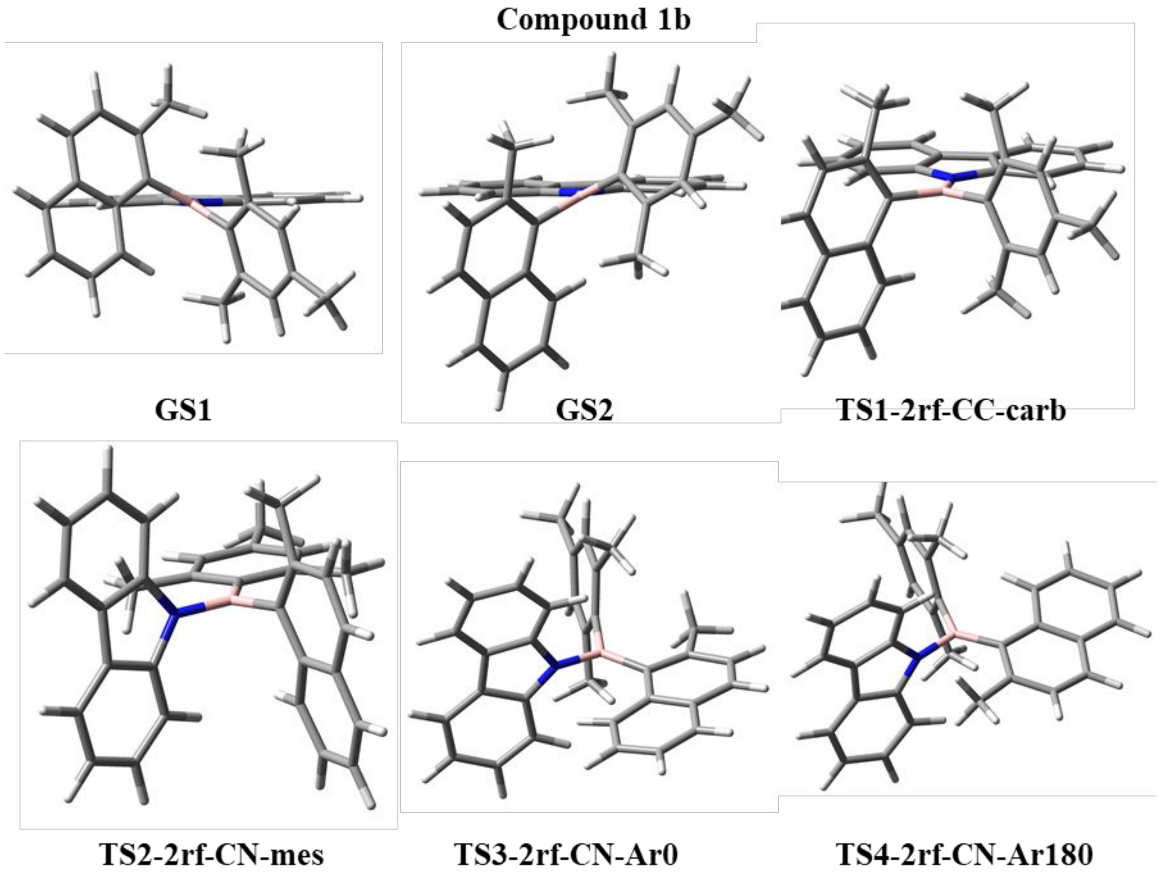

Figure S8. Calculated GSs and TSs for compound **1b**.

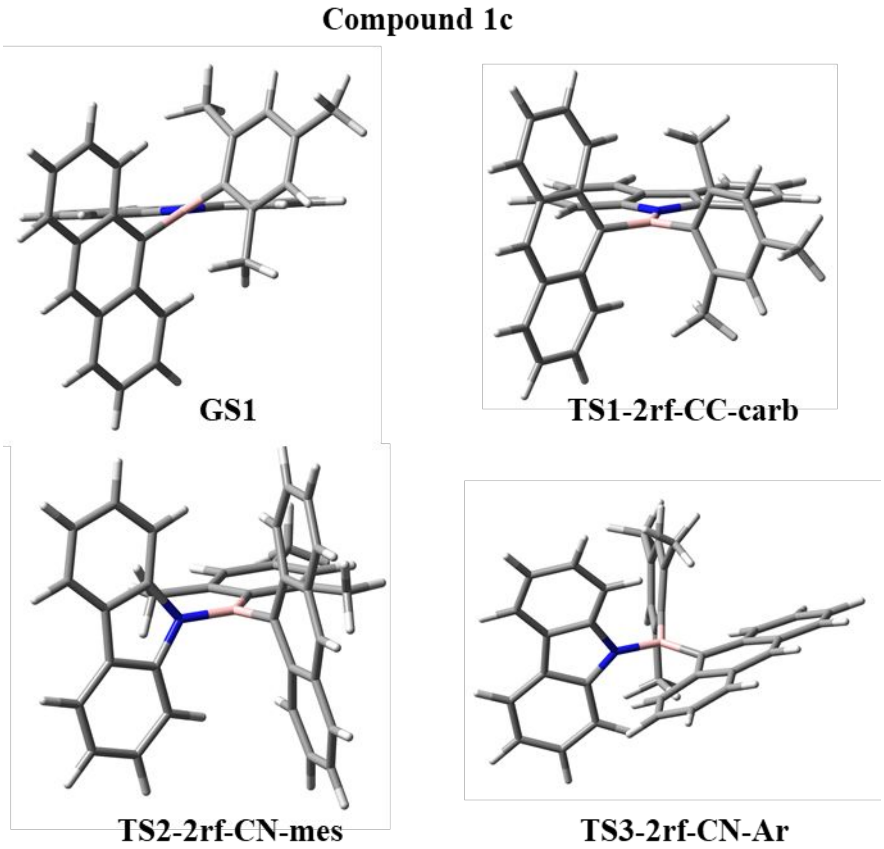

Figure S9. Calculated GSs and TSs for compound **1c**.

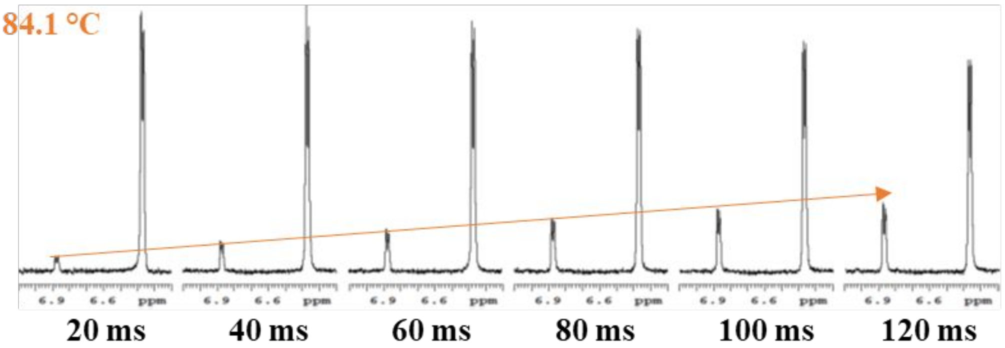

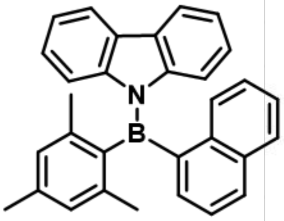

1a

	T (°C)		
	+81.6	+84.1	+86.7
t (s)	ln(X_{irr}-$X_{irr\ eq}$)	ln(X_{irr}-$X_{irr\ eq}$)	ln(X_{irr}-$X_{irr\ eq}$)
0.02	-0.793	-0.799	-0.810
0.04	-0.871	-0.925	-0.956
0.06	-0.973	-1.019	-1.083
0.08	-1.062	-1.121	-1.213
0.1	-1.126	-1.238	-1.339
0.12	-1.237	-1.334	-1.461
**$\Delta G^{\neq}$ (kcal/mol)**	20.32	20.34	20.35
		**20.3**	

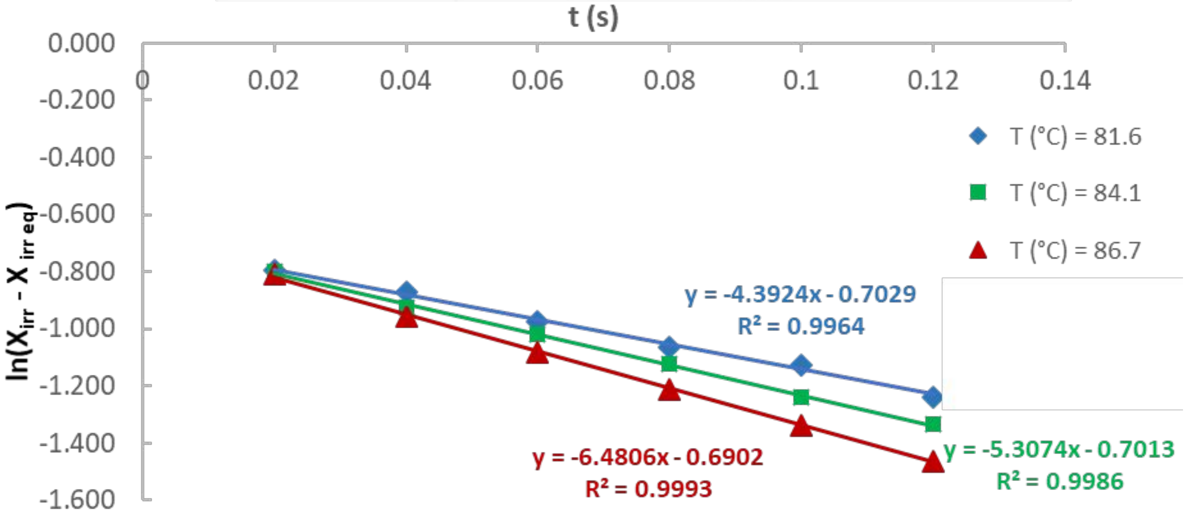

Figure S10. 1D-EXSY spectra were acquired at three different temperatures (+81.6, +84.1 and +86.7 °C) to derive three rate constants useful for the determination of the energy barrier of compound **1a**. The free energy barrier corresponds to the **TS4-2-rf-CN-Ar180**.

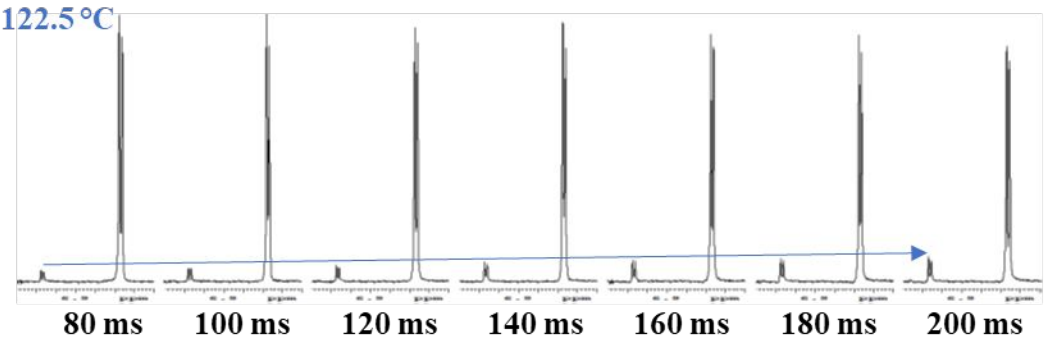

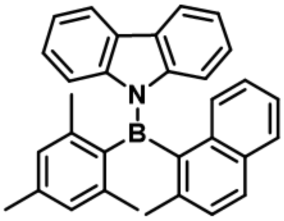

1b

	T (°C)	
	+122.5	+127.6
t (s)	$\ln(X_{irr}\text{-}X_{irr\,eq})$	$\ln(X_{irr}\text{-}X_{irr\,eq})$
0.08	-0.765	-0.813
0.1	-0.795	-0.836
0.12	-0.814	-0.868
0.14	-0.842	-0.900
0.16	-0.860	-0.930
0.18	-0.878	-0.952
0.2	-0.893	-0.983
**$\Delta G^{\neq}$ (kcal/mol)**	23.87	23.95
	**23.9**	

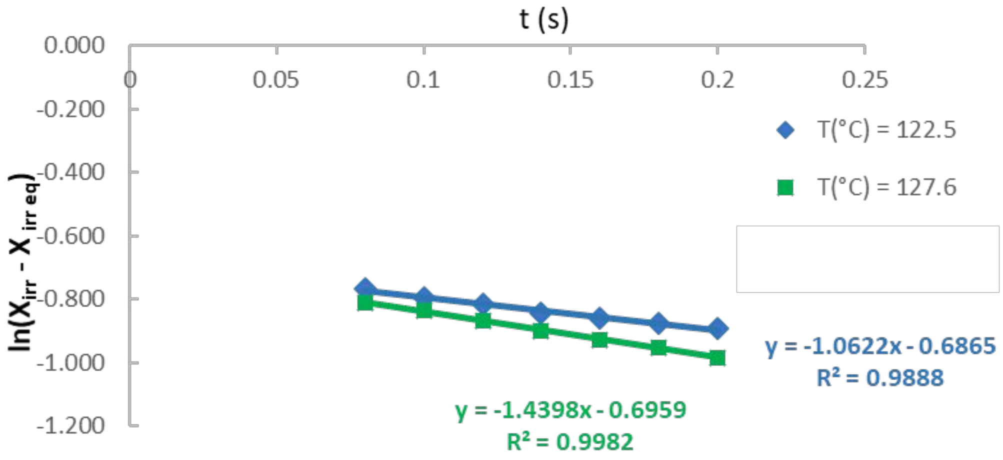

Figure S11. 1D-EXSY spectra were acquired at two different temperatures (+122.5 and +127.6 °C) to derive two rate constants useful for the determination of the energy barrier of compound **1b**. The free energy barrier corresponds to the **TS2-2-rf-CN-Mes**.

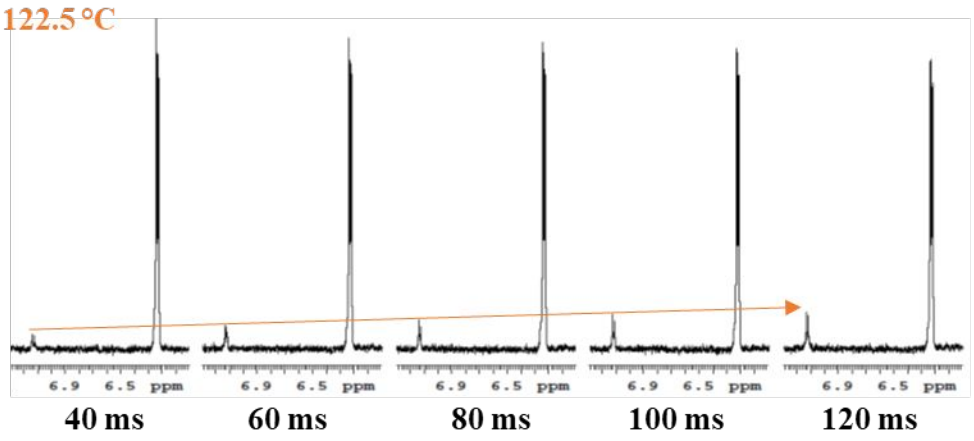

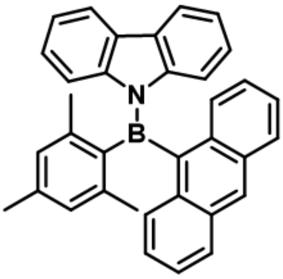

1c

	T (°C)	
	+117.4	+122.5
t (s)	$\ln(X_{irr}\text{-}X_{irr\ eq})$	$\ln(X_{irr}\text{-}X_{irr\ eq})$
0.04	-0.713	-0.770
0.06	-0.745	-0.826
0.08	-0.775	-0.858
0.1	-0.811	-0.904
0.12	-0.830	-0.948
**$\Delta G^{\neq}$ (kcal/mol)**	23.28	23.31
	**23.3**	

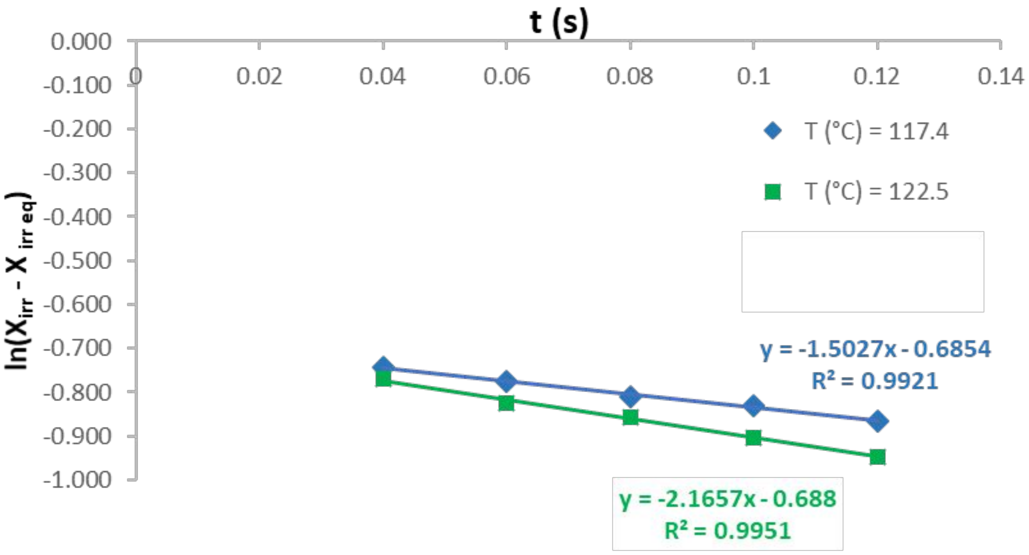

Figure S12. 1D-EXSY spectra were acquired at two different temperatures (+117.4 and +122.5 °C) to derive two rate constants useful for the determination of the energy barrier of compound **1c**. The free energy barrier corresponds to the **TS2-2-rf-CN-Mes**.

X-Ray **1b**

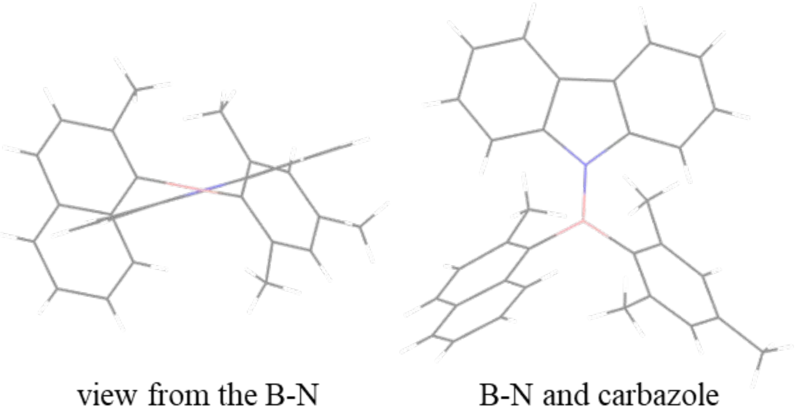

view from the B-N

B-N and carbazole on the sheet plane

Figure S13. Different views of X-ray structure of Compound **1b**.

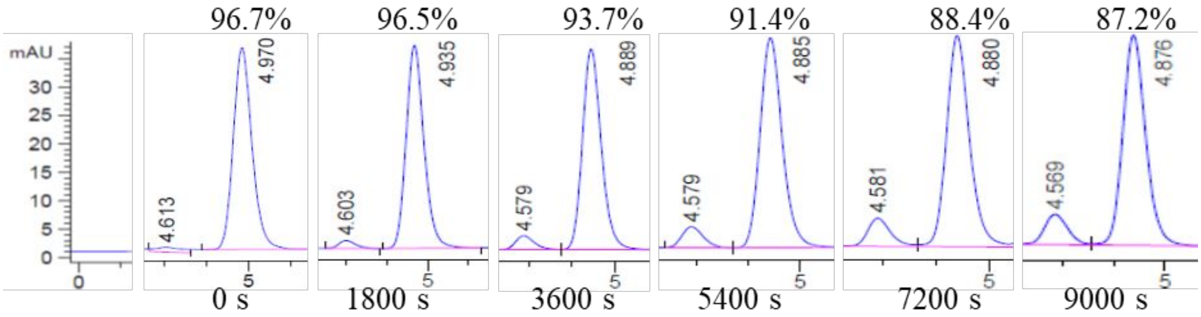

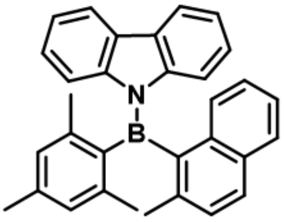

1b

t (s)	T +50 °C<br>$\ln(X_{en2}-X_{en2\ eq})$
0	-0.762
1800	-0.765
3600	-0.827
5400	-0.883
7200	-0.958
9000	-0.989
ΔG≠ (kcal/mol)	26.1

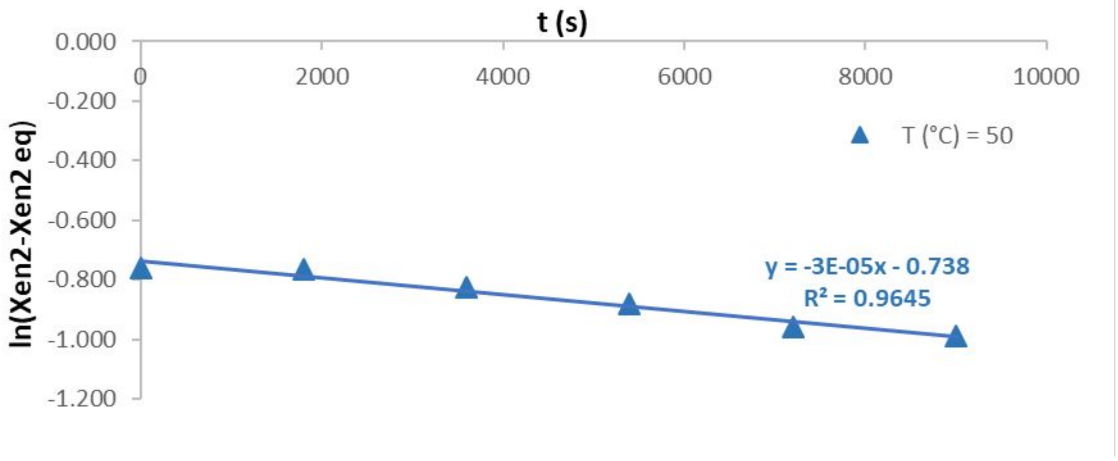

Figure S14. Kinetic analysis starting from the second eluted atropisomer of compound **1b**. Top: HPLC chromatograms. The free energy barrier corresponds to the **TS4-2-rf-CN-Ar180**. A mother solution of the second-eluted atropisomer was kept at +50 °C and aliquotes were taken at the indicated times and analyzed the interconversion ratio by CSP-HPLC at RT with CHIRALPAK AD-H column.

ECD of compound 1b.

The ECD spectra of compounds **1b** were acquired in the 190-400 nm region using a JASCO J-810 spectropolarimeter in Far-UV HPLC-grade acetonitrile solution. Concentration was about $1 \cdot 10^{-4}$ M, tuned by dilution in order to have a maximum absorbance between 0.8 and 1 with a cell path of 0.2 cm. The spectra were obtained by the average of 16 scans at 50 nm·min^{-1} scan rate.

The absolute configuration of the two atropisomers of **1b** was assigned by the simulation of ECD spectrum based on time-dependent density functional theory (TD-DFT). The conformational search suggested the existence of two conformations (GS1 32.6% and GS2 64.4%) due to the different dihedral angles of the two aryl rings with respect to the planar carbazole. The theoretical ECD spectra of the two ground state conformations (Figure S13) were obtained with four different functionals (CAM-B3LYP,[1] ωB97X-D,[2] BH&HLYP[3] and M06-2x[4]) with the same 6-311++G(2d,p) basis set, in order to have data redundancy, and to enhance reliability. The Boltzmann-averaged ECD spectrum is reported in the main article.

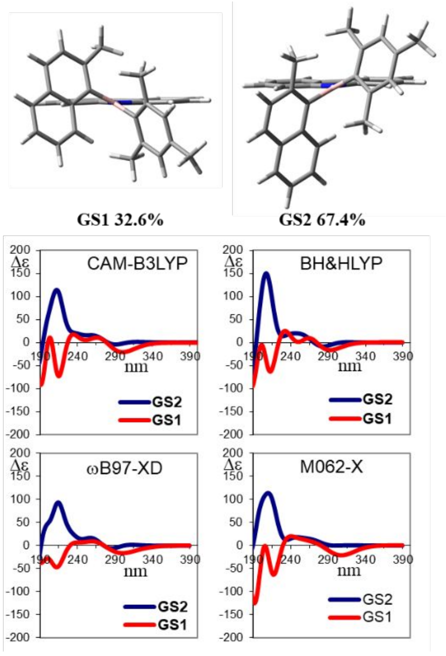

Figure S15. Computed spectra for the two conformations of the *M* atropisomer of compound **1b**. Calculations were obtained using the same 6-311++G(2d,p) basis set.

---

[1] Yanai, T.; Tew, D.; Handy, N. A new Hybrid Exchange−correlation Functional Using the Coulomb-attenuating Method (CAM-B3LYP). Chem. Phys. Lett. 2004, 393, 51−57.

[2] (a) Chai, J.-D.; Head-Gordon, M. Long-range Corrected Hybrid Density Functionals with Damped Atom−atom Dispersion Corrections. Phys. Chem. Chem. Phys. 2008, 10, 6615−6620.
(b) Iikura, H.; Tsuneda, T.; Yanai, T.; Hirao, K. A Long-range Correction Scheme for Generalized-gradient-approximation Exchange Functionals. J. Chem. Phys. 2001, 115, 3540−3544.

[3] In Gaussian 16 the BH&HLYP functional has the form: 0.5*EXHF + 0.5*EXLSDA + 0.5*ΔEXBecke88 + ECLYP.

[4] Zhao, Y.; Truhlar, D. G. The M06 Suite of Density Functionals for Main Group Thermochemistry, Thermochemical Kinetics, Noncovalent Interactions, Excited States, and Transition Elements: Two New Functionals and Systematic Testing of Four M06-class Functionals and 12 other Functionals. Theor. Chem. Acc. 2008, 120, 215−241.

Photophysical Measurements

Absorption spectra were recorded at room temperature using a Cary 100 UV/vis spectrometer, Agilent. Uncorrected steady-state emission and excitation spectra were recorded on an Edinburgh FLSP920 spectrometer equipped with a 450 W xenon arc lamp, double excitation and single emission monochromators, and a Peltier-cooled Hamamatsu R928P photomultiplier tube (185−850 nm). Emission and excitation spectra were acquired with a cut-off filter (340 nm) and corrected for source intensity (lamp and grating) and emission spectral response (detector and grating) by a calibration curve supplied with the instrument. Quantum yields (Φ) were determined using the optically dilute method by Crosby and Demas[5] at excitation wavelength obtained from absorption spectra on a wavelength scale [nm] and compared to the reference emitter by the following equation:[6]

$$\phi_s = \phi_r \left[\frac{A_r(\lambda_r)}{A_s(\lambda_s)}\right] \left[\frac{I_r(\lambda_r)}{I_s(\lambda_s)}\right] \left[\frac{n_s^2}{n_r^2}\right] \left[\frac{D_s}{D_r}\right]$$

where A is the absorbance at the excitation wavelength (λ), I is the intensity of the excitation light at the excitation wavelength (λ), n is the refractive index of the solvent, D is the integrated intensity of the luminescence, and Φ is the quantum yield. The subscripts r and s refer to the reference and the sample, respectively. A stock solution with an absorbance > 0.1 was prepared, then a 10 times diluted solution was obtained. The Lambert-Beer law was assumed to remain linear at the concentration of the solutions. The degassed measurements were obtained after the solutions were bubbled for 10 minutes under Ar atmosphere, using a septa-sealed quartz cell. Air-equilibrated Quinine Sulphate in 0.05 M H_2SO_4 (Φ = 0.52)[7] was used as reference. The quantum yield determinations were performed at identical excitation wavelengths for the sample and the reference, therefore deleting the I(λr)/I(λs) term in the equation. Emission lifetimes (τ) were determined with the single photon counting technique (TCSPC) with the same Edinburgh FLSP920 spectrometer using pulsed picosecond LED (EPLED 340, FWHM < 800ps) as the excitation source, with repetition rates between 1 kHz and 1 MHz, and the above-mentioned R928P PMT as detector. The goodness of fit was assessed by minimizing the reduced $\chi 2$ function and by visual inspection of the weighted residuals. To record the neat solid matrix luminescence spectra, the samples were placed on carbon tape adhered on a quartz cuvette for solid samples. The solvent used in the preparation of the solutions for the photophysical investigations was of spectrometric grade. Experimental uncertainties are estimated to be ±8% for lifetime determinations, ±20% for quantum yields, and ±2 nm and ±5 nm for absorption and emission peaks, respectively.

---

[5] *G. A. Crosby and J. N. Demas "Measurement of photoluminescence quantum yields. Review.", J. Phys. Chem., 1971,* ***75****, 991-1024.*

[6] *F. Eaton "Reference materials for luminescence measurements", Pure Appl. Chem., 1988,* ***60****, 1107-1114.*

[7] A. M. Brouwer, *Pure Appl. Chem.*, 83, 2001, 2213.

Photophysical Properties: Absorption and emission spectra

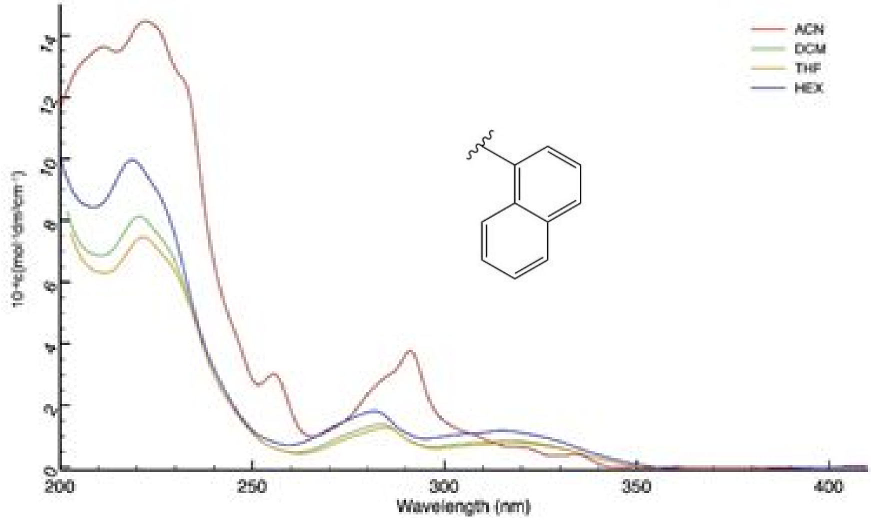

Figure S16. Compound **1a**: Absorption profiles obtained from dilute (10^{-5} M) solutions in hexane (HEX), tetrahydrofuran (THF), dichloromethane (DCM) and acetonitrile (ACN).

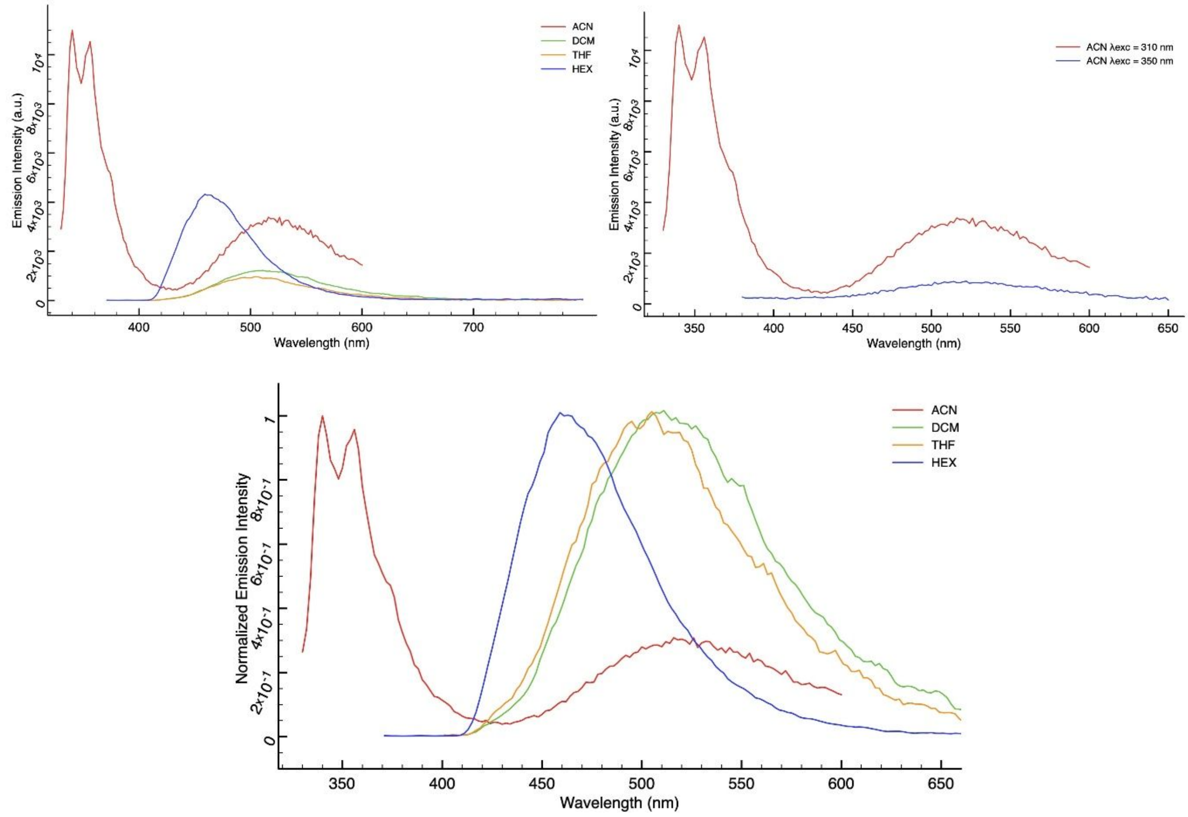

Figure S17. Compound **1a**: (top left) emission profiles obtained (λ_{exc} =310 nm) from dilute (10^{-5} M) solutions in hexane (HEX), tetrahydrofuran (THF), dichloromethane (DCM) and acetonitrile (ACN); (top right) emission profiles obtained from dilute (10^{-5} M) ACN solution at λ_{exc} =310 nm and λ_{exc} =350 nm; (bottom), normalized profiles.

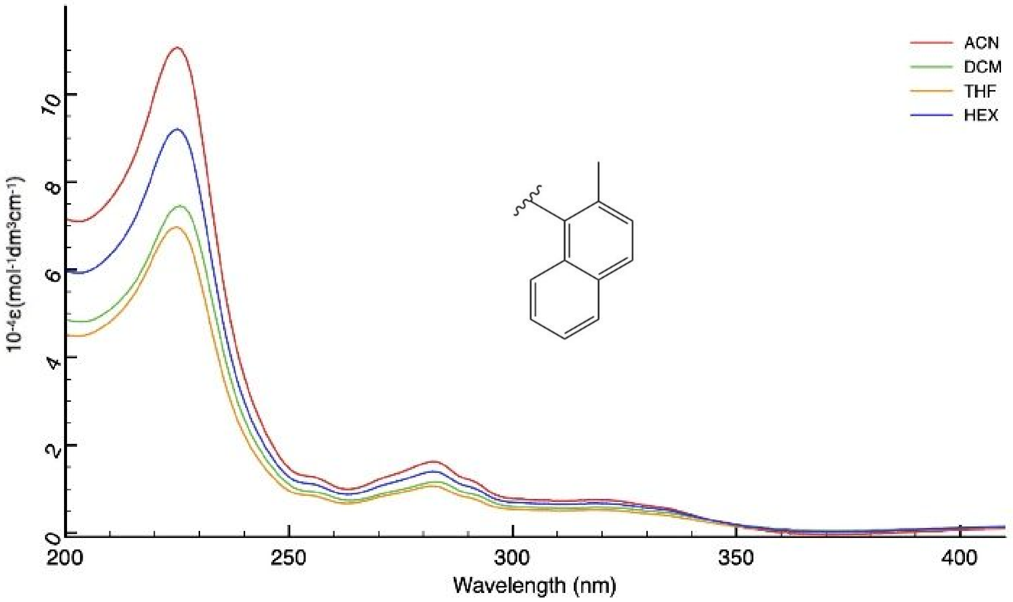

Figure S18. Compound **1b**: Absorption profiles obtained from dilute (10^{-5} M) solutions in hexane (HEX), tetrahydrofuran (THF), dichloromethane (DCM) and acetonitrile (ACN).

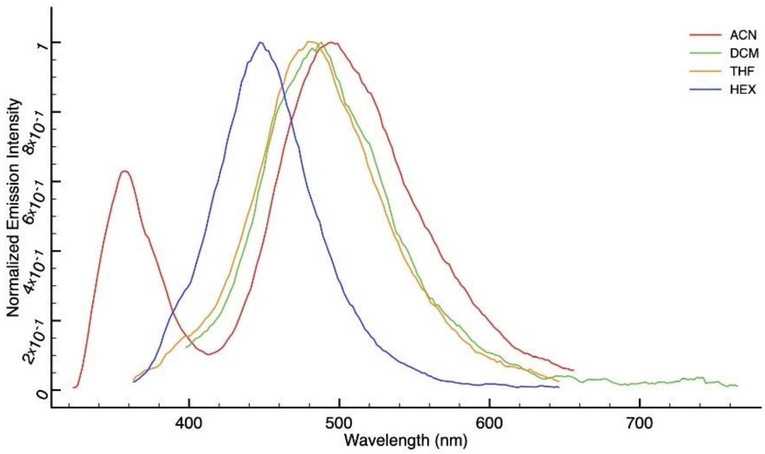

Figure S19. Compound **1b**: (top) emission profiles obtained (λ_{exc} =310 nm) from dilute (10^{-5} M) solutions in hexane (HEX), tetrahydrofuran (THF), dichloromethane (DCM) and acetonitrile (ACN); (bottom), normalized profiles.

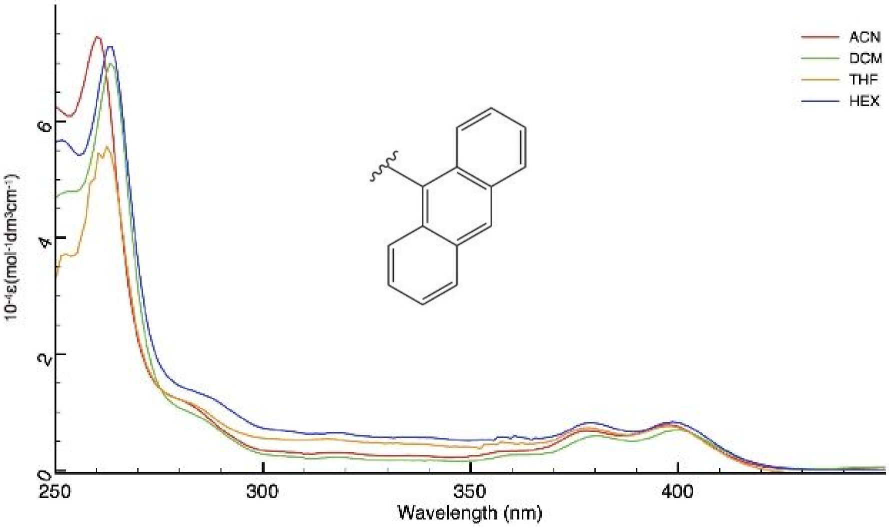

Figure S20. Compound **1c**: Absorption profiles obtained from dilute (10^{-5} M) solutions in hexane (HEX), tetrahydrofuran (THF), dichloromethane (DCM) and acetonitrile (ACN).

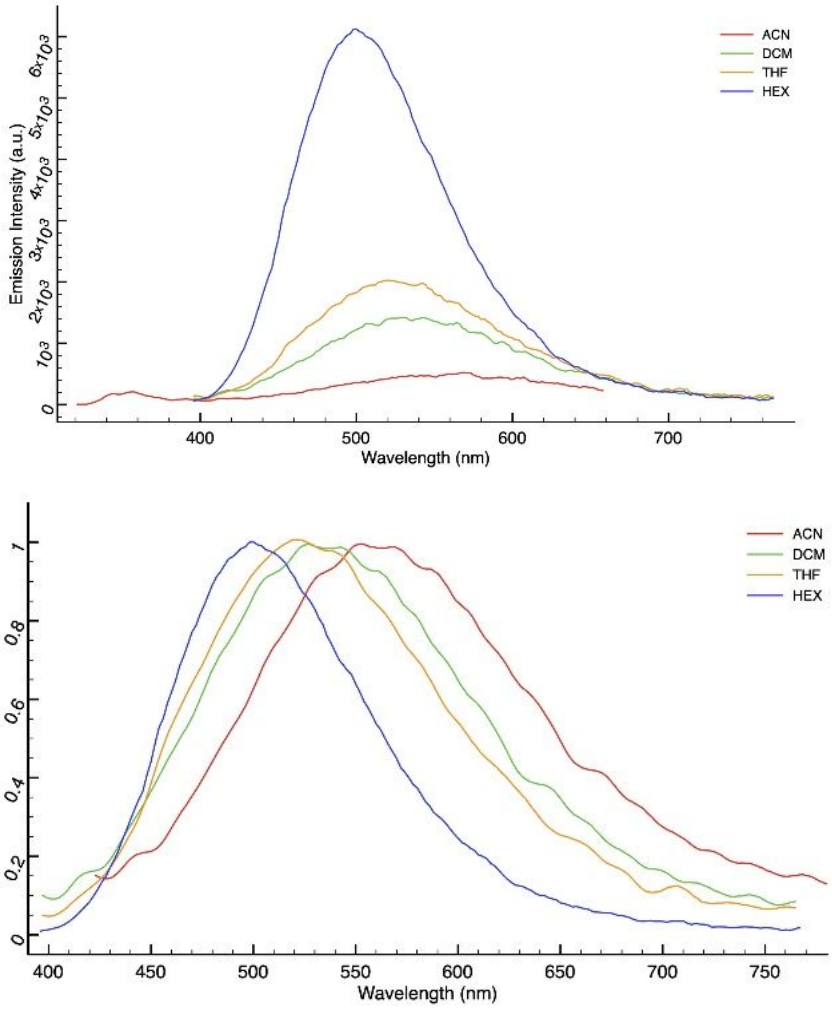

Figure S21. Compound **1c**: (top) emission profiles obtained (λ_{exc} =310 nm) from dilute (10^{-5} M) solutions in hexane (HEX), tetrahydrofuran (THF), dichloromethane (DCM) and acetonitrile (ACN); (bottom), normalized profiles.

DFT and TD-DFT calculations of Photophysical properties.

A 7-step sequence was employed for the calculation of the emission properties con compounds **1a-c** using DFT and TD-DFT, and following the methodology of a previous work [1a].

An example follows for the GS1 conformation of compound **1a**. For all compounds the results are summarized in table S10.

Step1

```
Method: cam-b3lyp/6-31G(d) opt freq
Solvent : Acetonitrile
SCF Done: E(RCAM-B3LYP) = -1275.91938953 a.u.
Lowest frequency =  22.9941
```

Standard orientation:

Center Number	Atomic Number	Atomic Type	Coordinates (Angstroms) X	Y	Z
1	5	0	0.095935	0.000590	0.215993
2	6	0	1.536460	1.578154	2.374849
3	1	0	0.654874	1.100217	2.813356
4	1	0	2.309764	0.807858	2.293703
5	1	0	1.890975	2.333080	3.081434
6	6	0	0.295478	2.151180	-1.202270
7	6	0	0.737357	3.457959	-1.398093
8	1	0	0.545227	3.941807	-2.352981
9	6	0	1.408621	4.163013	-0.403152
10	6	0	1.639750	3.518718	0.809135
11	1	0	2.152622	4.054535	1.604508
12	6	0	1.236685	2.203433	1.031900
13	6	0	0.545946	1.499549	0.024508
14	6	0	-0.447626	1.470843	-2.330767
15	1	0	-1.518736	1.397655	-2.115397
16	1	0	-0.335636	2.034960	-3.260193
17	1	0	-0.088400	0.453689	-2.517076
18	6	0	1.892201	5.569707	-0.639418
19	1	0	1.914072	6.145349	0.290134
20	1	0	2.909787	5.571914	-1.047388
21	1	0	1.254332	6.096320	-1.354757
22	7	0	-1.300330	-0.353695	0.108573
23	6	0	-2.413062	0.483529	0.380173
24	6	0	-1.836628	-1.590235	-0.340025
25	6	0	-3.608531	-0.211472	0.129896
26	6	0	-2.445318	1.781227	0.890425
27	6	0	-3.240216	-1.530678	-0.340964
28	6	0	-4.844337	0.391536	0.357686
29	6	0	-3.683959	2.370740	1.108290
30	1	0	-1.537370	2.323274	1.115723
31	6	0	-3.999780	-2.615844	-0.775940
32	6	0	-4.876715	1.689589	0.842767
33	1	0	-5.764580	-0.150667	0.162311
34	1	0	-3.721705	3.382676	1.499392
35	1	0	-5.084375	-2.564280	-0.770744
36	1	0	-5.828691	2.177289	1.026057
37	6	0	-1.948877	-3.794875	-1.254317
38	1	0	-1.448041	-4.683179	-1.626397

39	6	0	-1.180078	-2.721795	-0.823678
40	1	0	-0.101200	-2.774098	-0.869599
41	6	0	-3.347205	-3.752748	-1.225316
42	1	0	-3.919931	-4.608260	-1.568486
43	6	0	1.130071	-1.121923	0.595939
44	6	0	2.427419	-1.291987	0.004374
45	6	0	2.848262	-0.564982	-1.141651
46	6	0	3.332371	-2.246419	0.553569
47	6	0	1.703964	-2.839033	2.243197
48	6	0	4.085175	-0.762671	-1.697950
49	1	0	2.176467	0.163498	-1.579565
50	6	0	4.606504	-2.429870	-0.042954
51	6	0	2.942826	-3.004195	1.687577
52	1	0	1.406880	-3.421885	3.109325
53	6	0	4.978787	-1.705733	-1.143307
54	1	0	4.381571	-0.192514	-2.572930
55	1	0	5.281498	-3.162468	0.390771
56	1	0	3.645876	-3.721247	2.102380
57	1	0	5.955187	-1.854620	-1.593458
58	6	0	0.802602	-1.907692	1.685038
59	1	0	-0.176725	-1.800144	2.142779

Step2

Method: cam-b3lyp/6-31+G(d,p) TD(NStates=3) SCRF(solvent=acetonitrile)

Excited State 1: Singlet-A 4.0726 eV 304.43 nm f=0.3144 <S**2>=0.000
112 ->113 0.66488

Step3

Method: cam-b3lyp/6-31+G(d,p) SCRF(Solvent=acetonitrile,NonEquilibrium=Save)

SCF Done: E(RCAM-B3LYP) = -1275.99591420 a.u.

Step4

Method: CAM-B3LYP/6-31+G(d,p) TD(NStates=1,Root=1) SCRF(Solvent=acetonitrile,ExternalIteration,NonEquilibrium=Read)

After PCM corrections, the energy is -1275.84485991 a.u.

Excited State 1: Singlet-A 4.0881 eV 303.28 nm f=0.2467 <S**2>=0.000
112 ->113 0.66323

Step5

Method: cam-b3lyp/6-31G(d) opt freq(savenormalmodes) TD(NStates=1,Root=1)

Total Energy, E(TD-HF/TD-DFT) = -1275.79010600 a.u.
Lowest frequency = 24.1040

Standard orientation:

Center Atomic Atomic Coordinates (Angstroms)

Number	Number	Type	X	Y	Z
1	5	0	0.222068	-0.034174	0.038565
2	6	0	1.907854	1.621867	1.942515
3	1	0	1.159618	0.999276	2.442596
4	1	0	2.769100	0.974737	1.747013
5	1	0	2.229017	2.402238	2.638231
6	6	0	0.131860	2.145013	-1.408462
7	6	0	0.428937	3.490676	-1.619319
8	1	0	0.059120	3.975930	-2.520371
9	6	0	1.186651	4.227994	-0.715491
10	6	0	1.642740	3.572861	0.425570
11	1	0	2.222574	4.130958	1.158057
12	6	0	1.371956	2.227494	0.667655
13	6	0	0.603629	1.471652	-0.255851
14	6	0	-0.691311	1.430446	-2.451475
15	1	0	-1.727760	1.284348	-2.125493
16	1	0	-0.723076	2.000567	-3.384105
17	1	0	-0.283794	0.438426	-2.672224
18	6	0	1.521620	5.674652	-0.970367
19	1	0	1.595645	6.238146	-0.035411
20	1	0	2.484813	5.775814	-1.484978
21	1	0	0.766470	6.155134	-1.599143
22	7	0	-1.284406	-0.310587	0.038063
23	6	0	-2.253115	0.328716	0.810908
24	6	0	-1.927105	-1.259881	-0.725236
25	6	0	-3.530319	-0.215653	0.544585
26	6	0	-2.070414	1.353410	1.735036
27	6	0	-3.319501	-1.261247	-0.453460
28	6	0	-4.638584	0.268253	1.208223
29	6	0	-3.200724	1.828588	2.399844
30	1	0	-1.090555	1.774402	1.918013
31	6	0	-4.140949	-2.158045	-1.096931
32	6	0	-4.462176	1.299420	2.141987
33	1	0	-5.627368	-0.136149	1.018890
34	1	0	-3.092387	2.626160	3.126658
35	1	0	-5.208436	-2.182167	-0.905391
36	1	0	-5.324977	1.690397	2.670278
37	6	0	-2.200280	-3.052439	-2.276049
38	1	0	-1.788815	-3.757724	-2.989377
39	6	0	-1.350378	-2.154648	-1.632351
40	1	0	-0.286087	-2.136588	-1.825476
41	6	0	-3.567059	-3.056221	-2.014916
42	1	0	-4.206498	-3.766718	-2.527316
43	6	0	1.078131	-1.233214	0.441839
44	6	0	2.514703	-1.354216	0.198127
45	6	0	3.240807	-0.391524	-0.539069
46	6	0	3.232630	-2.499713	0.659057
47	6	0	1.188817	-3.460507	1.520904
48	6	0	4.594469	-0.513027	-0.774283
49	1	0	2.711440	0.466036	-0.934652
50	6	0	4.624016	-2.597800	0.412789
51	6	0	2.549136	-3.545261	1.334866
52	1	0	0.658657	-4.264140	2.025037
53	6	0	5.299399	-1.623882	-0.282717
54	1	0	5.113417	0.249228	-1.347549
55	1	0	5.150515	-3.476171	0.777219
56	1	0	3.111700	-4.407486	1.680536
57	1	0	6.365729	-1.719123	-0.464250

```
    58          6           0        0.473170   -2.343044    1.081819
    59          1           0       -0.589231   -2.314864    1.295466
 ---------------------------------------------------------------------
```

Step6

```
 Method: cam-b3lyp/6-31+G(d,p) TD(NStates=1, Root=1)
SCRF(Solvent=acetonitrile,ExternalIteration,NonEquilibrium=Save)
 After PCM corrections, the energy is -1275.89241268 a.u.

 Excited State   1:      Singlet-A      2.5045 eV  495.05 nm  f=0.0472
<S**2>=0.000
     112 ->113        -0.67924
     112 ->114         0.12873
```

Step7

```
  Method: cam-b3lyp/6-31+G(d,p) SCRF(Solvent=acetonitrile,
NonEquilibrium=Read)

 SCF Done: E(RCAM-B3LYP) = -1275.97212926 a.u.

                              Results
 -------------------------------------------------------------------
      Absorb Energy     =  | 0.151 a.u. | 4.110 eV | 301.636 nm
 --------------------------|------------|----------|----------------
      Emission Energy   =  | 0.080 a.u. | 2.169 eV | 571.567 nm
 --------------------------|------------|----------|----------------
      Stokes Shift      =  | 0.071 a.u. | 1.941 eV | 269.931 nm
 -------------------------------------------------------------------
```

Table *S2* Emission wavelengths, absorption wavelengths and Stokes shift calculated in four different solvents for compounds **1a-c.**

**Compound**	**Starting Geometry**	**solvent**	**absorption wavelength (nm) S0-S1***	**emission wavelength (nm) S1-S0***	**Stokes shift (nm)**	**Stokes shift (cm^{1-})**
**1a**	GS1	HEX	305	473	168	59524
		DCM	302	544	241	41494
		THF	303	540	237	42194
		ACN	302	572	270	37037
	GS2	HEX	295	441	147	68027
		DCM	294	496	202	49505
		THF	294	493	199	50251
		ACN	293	517	223	44843
**1b**	GS1	HEX	306	433	127	78740
		DCM	305	474	169	59172
		THF	305	472	167	59880
		ACN	304	489	185	54054
	GS2	HEX	299	427	128	78125
		DCM	298	450	152	65789
		THF	298	449	151	66225
		ACN	297	457	161	62112
**1c**	GS1	HEX	363	445	82	121951
		DCM	363	449	86	116279
		THF	363	449	86	116279
		ACN	363	450	87	114942

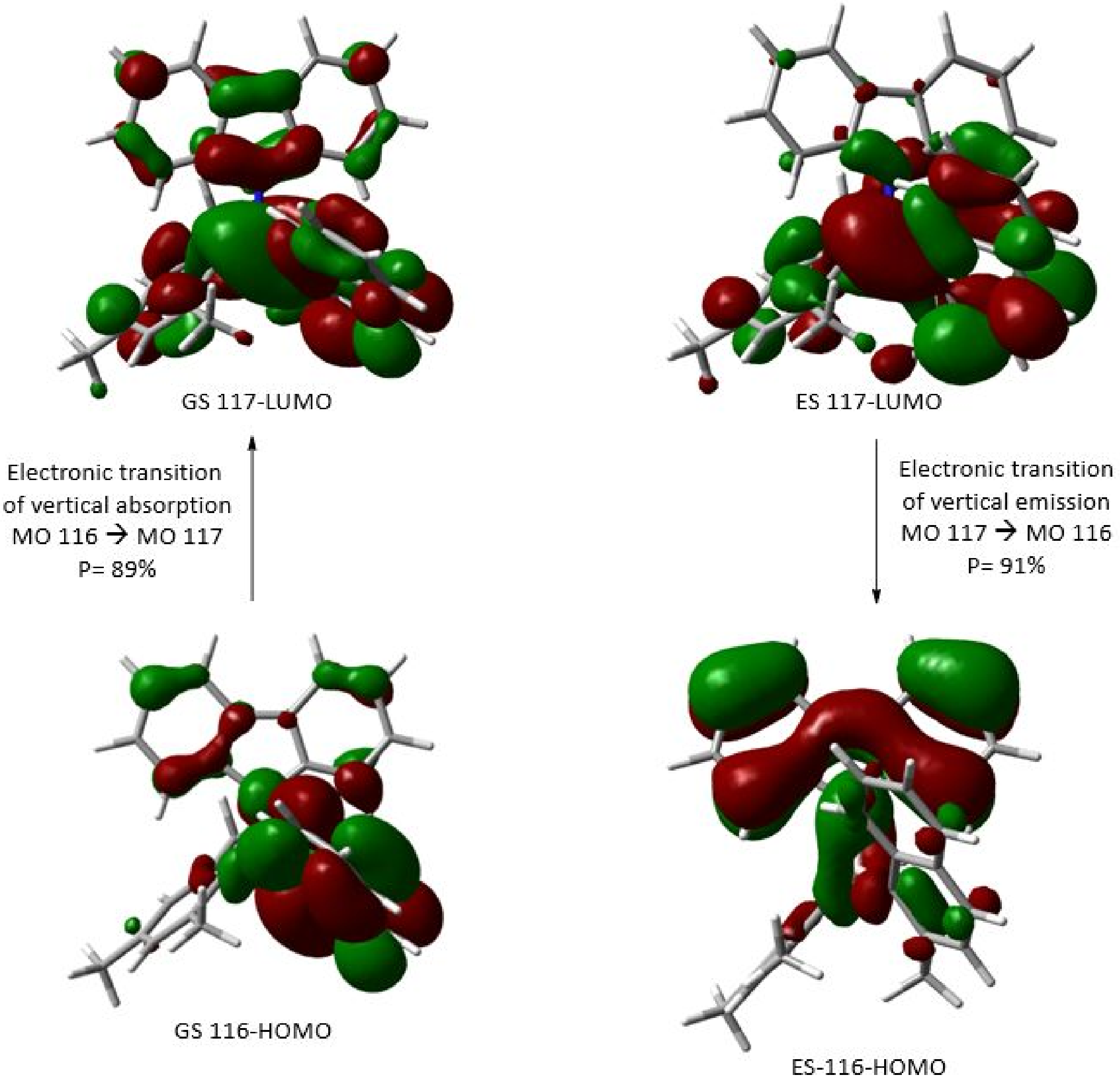

Figure S22. Compound **1b,** GS2 conformation, THF as solvent: representation of the HOMO (116) and LUMO (117) calculated orbitals involved with the highest probability in vertical absorption and emission transitions. On the left are reported the MO related to the GS geometry, on the right the optimized geometry of the excited state.

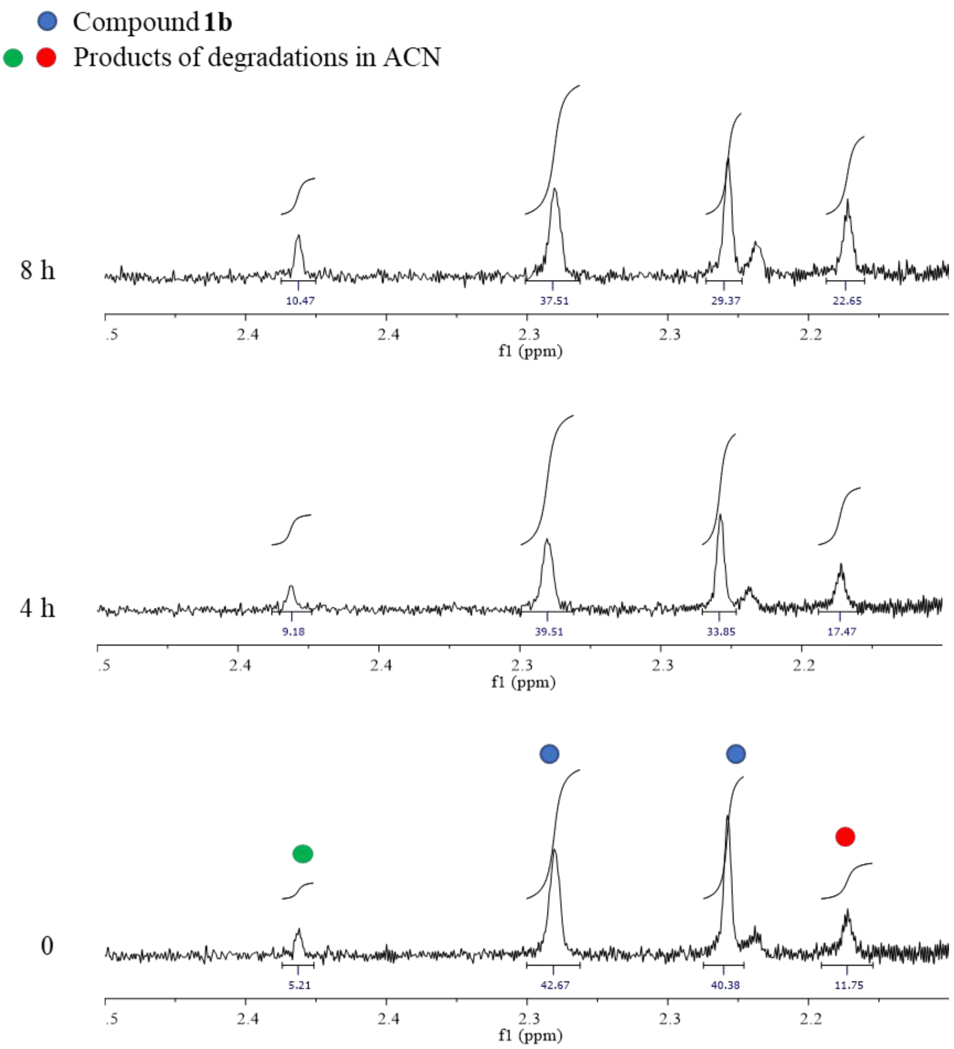

Figure S23. Degradation of Compound **1b** in deuterate ACN. Zoom of aliphatic ^{1}H NMR spectra at 3 different times.

Circularly polarized luminescence measurements

General

Circularly polarized luminescence (CPL) measurements were performed using a homemade spectrofluoropolarimeter.[8] The spectra were run in three different solvent increasing the polarity using hexane, CH_2Cl_2 (DCM) and CH_3CN (ACN) in ~$1\cdot10^{-4}$ M solutions. The samples were excited by a 365 nm radiation with a 90° geometry between excitation and detection. The following parameters were used: slit-width ~10 nm, int time 2 sec, scan speed 2 nm/sec, PMT voltage 500 V, accumulations 8. The spectra were corrected by subtracting the racemate spectrum measured in the same conditions. ECD spectra were measured with a Jasco J-1500 spectropolarimeter on the same solutions employed for CPL using a 0.2 cm cell.

[8] F. Zinna, T. Bruhn, C. A. Guido, J. Ahrens, M. Bröring, L. Di Bari, G. Pescitelli, *Chem. Eur. J.*, **2016**, *22(45)*, 16089-16098.

CPL/ECD Spectra HEXANE

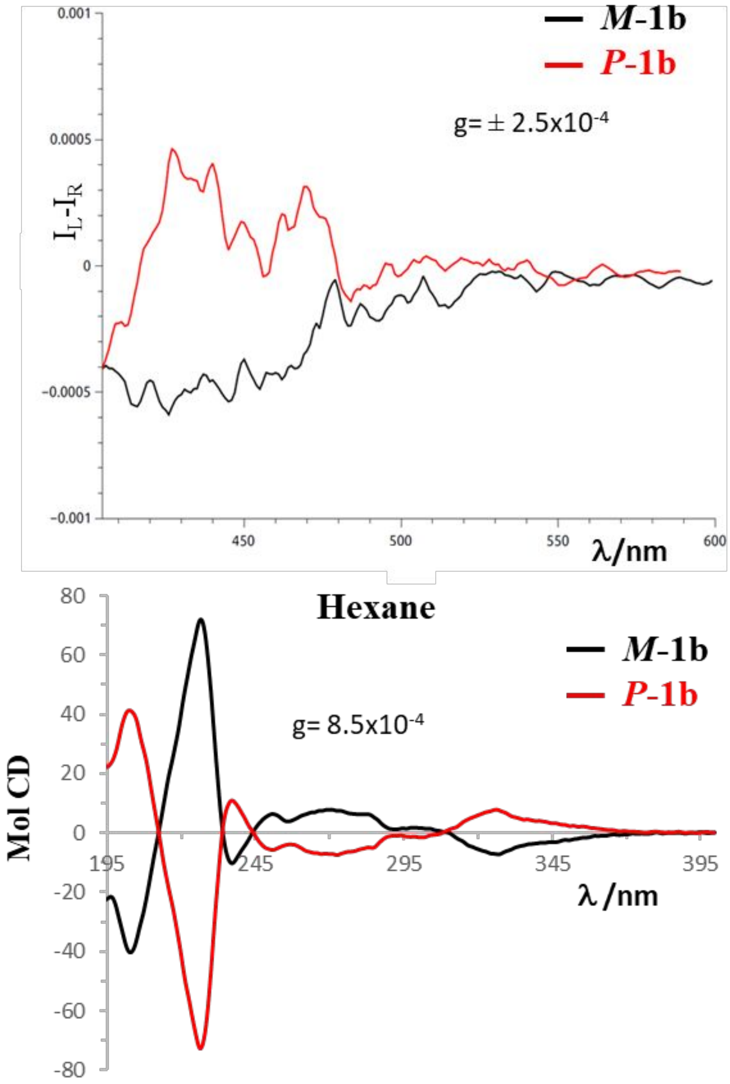

Figure S24. CPL (top) and ECD (bottom) spectra of ***M*-1b** and ***P*-1b** samples in hexane solution.

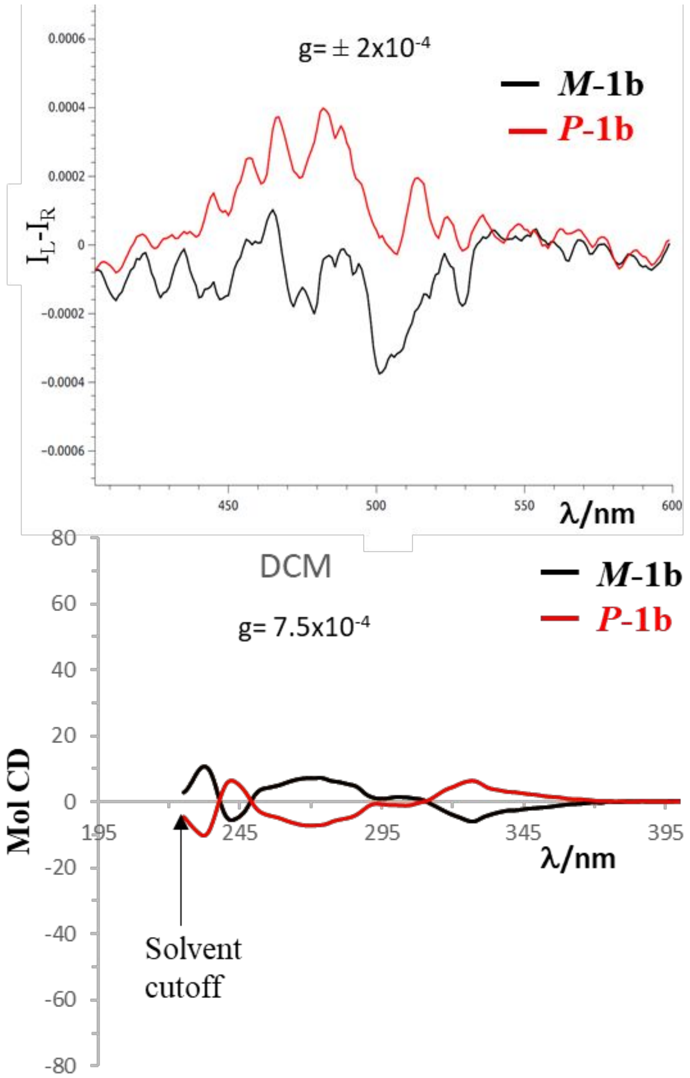

Figure S25. CPL *(top) and ECD (bottom)* spectra *of* **M-*1b*** *and* **P-*1b*** samples in DCM solution.

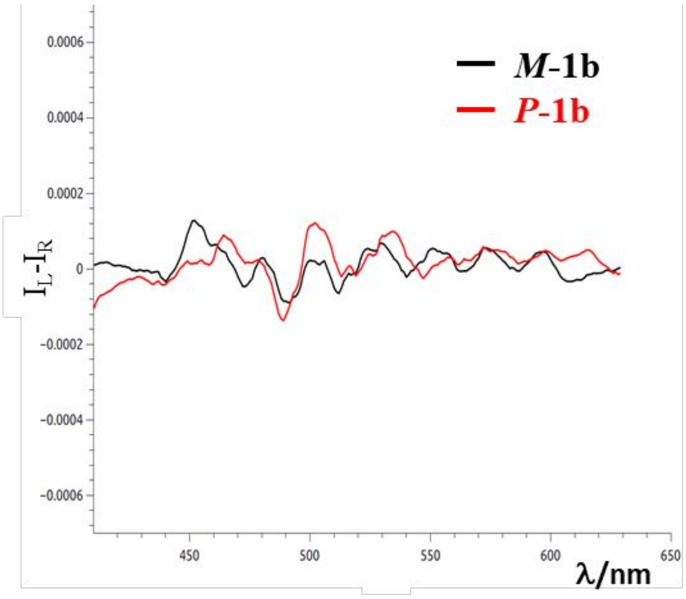

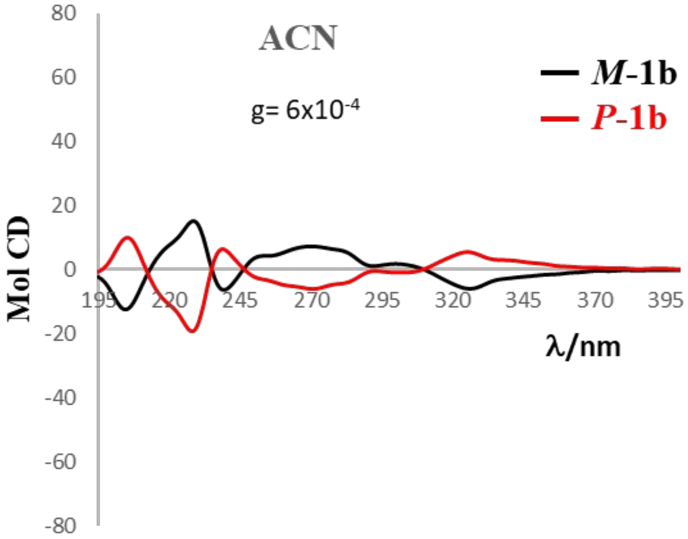

Figure S26. CPL *(top) and ECD (bottom)* spectra *of* **M-*1b*** *and* **P-*1b*** samples in ACN solution.

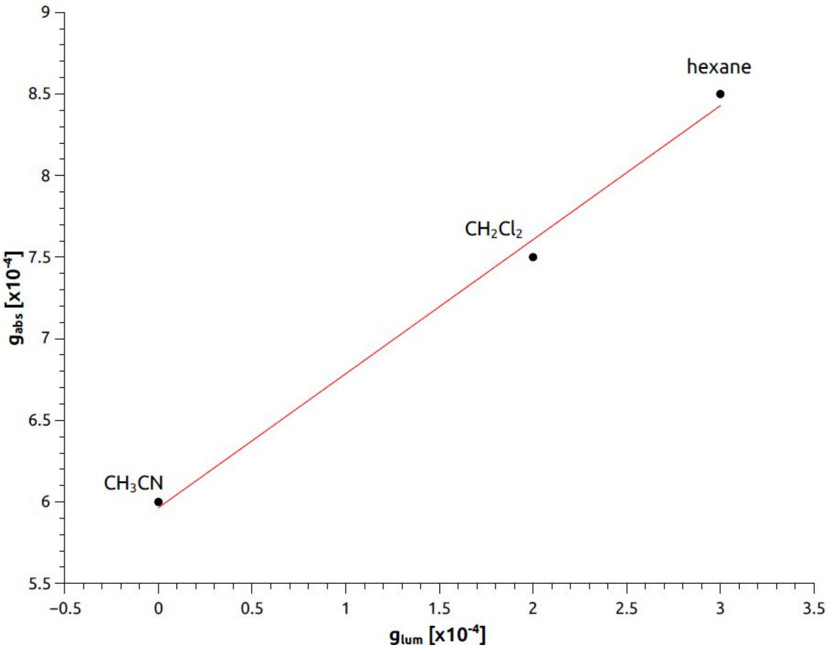

Figure S27. Comparison between g_{abs} and g_{lum} for ***P-1b*** in different solvents. The red line represents a linear fitting (R^2 = 0.99).

Crystal Structure Report for compound 1b

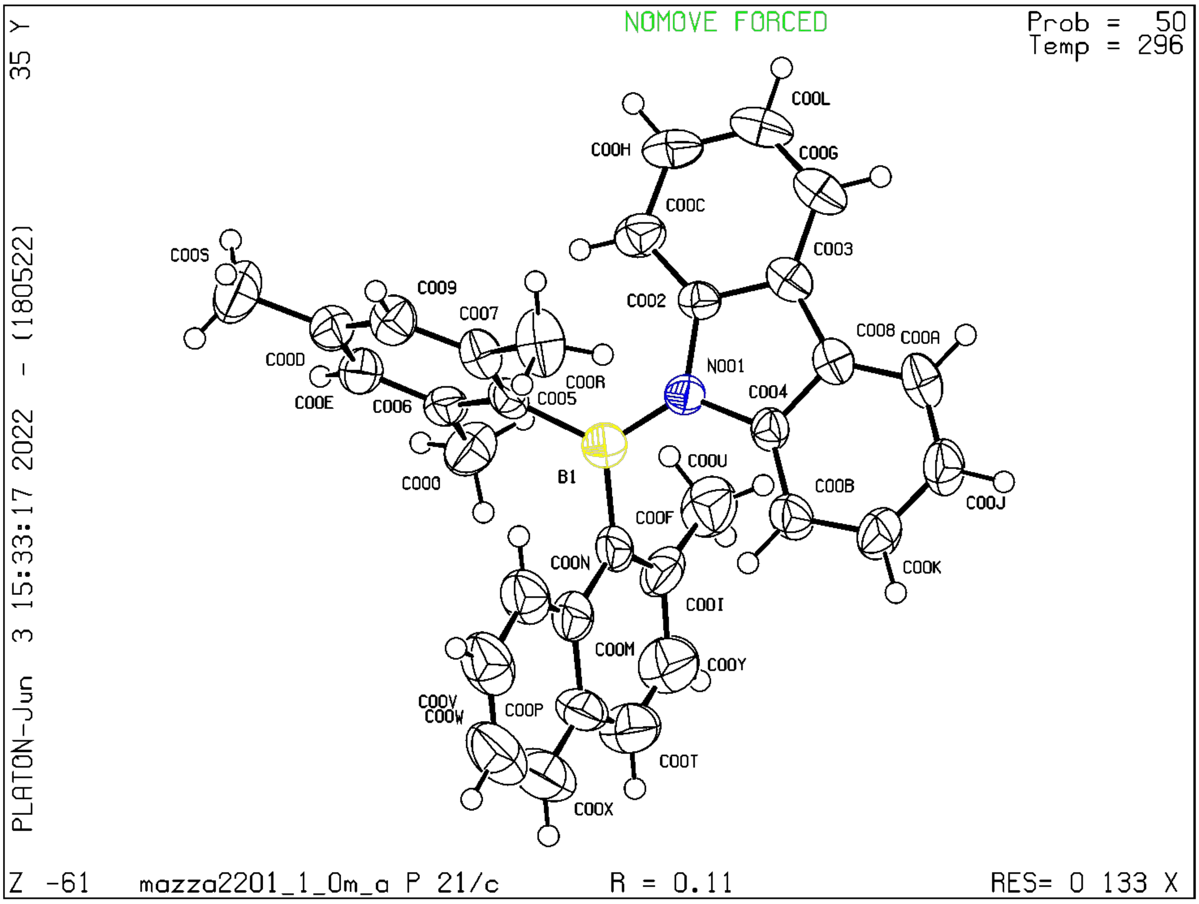

A specimen of **1b**, approximate dimensions 0.150 mm x 0.300 mm x 0.300 mm, was used for the X-ray crystallographic analysis. The X-ray intensity data were measured using Mo-Kα radiation (λ = 0.71073 Å). The integration of the data using a monoclinic unit cell yielded a total of 23028 reflections to a maximum θ angle of 25.00° (0.84 Å resolution), of which 4065 were independent (average redundancy 5.665, completeness = 97.6%, R_{int} = 6.93%, R_{sig} = 5.50%) and 2935 (72.20%) were greater than $2\sigma(F^2)$. The final cell constants of a =16.915(15) Å, b = 7.941(7) Å, c = 17.779(16) Å, β = 98.39(5)°, volume = 2363.(4) Å^3, are based upon the refinement of the XYZ-centroids of reflections above 20 σ(I). The calculated minimum and maximum transmission coefficients (based on crystal size) are 0.9790 and 0.9900.

The structure was solved and refined using the Bruker SHELXTL Software Package, using the space group P 1 21/c 1, with Z = 4 for the formula unit, $C_{32}H_{28}BN$. The final anisotropic full-matrix least-squares refinement on F^2 with 311 variables converged at R1 = 10.87%, for the observed data and wR2 = 25.77% for all data. The goodness-of-fit was 1.162. The largest peak in the final difference electron density synthesis was 0.387 e$^-$/Å^3 and the largest hole was -0.243 e$^-$/Å^3 with an RMS deviation of 0.055 e$^-$/Å^3. On the basis of the final model, the calculated density was 1.230 g/cm^3 and F(000), 928 e$^-$.

Table S3. Sample and crystal data.

**Identification code**	mazza2201	
**Chemical formula**	$C_{32}H_{28}BN$	
**Formula weight**	437.36 g/mol	
**Temperature**	296(2) K	
**Wavelength**	0.71073 Å	
**Crystal size**	0.150 x 0.300 x 0.300 mm	
**Crystal system**	monoclinic	
**Space group**	P 1 21/c 1	
**Unit cell dimensions**	a = 16.915(15) Å	α = 90°
	b = 7.941(7) Å	β = 98.39(5)°
	c = 17.779(16) Å	γ = 90°
**Volume**	2363.(4) $Å^3$	
**Z**	4	
**Density (calculated)**	1.230 g/cm^3	
**Absorption coefficient**	0.070 mm^{-1}	
**F(000)**	928	

Table S4. Data collection and structure refinement.

**Theta range for data collection**	2.43 to 25.00°	
**Index ranges**	-20<=h<=20, -9<=k<=9, -21<=l<=21	
**Reflections collected**	23028	
**Independent reflections**	4065 [R(int) = 0.0693]	
**Max. and min. transmission**	0.9900 and 0.9790	
**Structure solution technique**	direct methods	
**Structure solution program**	SHELXT 2014/5 (Sheldrick, 2014)	
**Refinement method**	Full-matrix least-squares on F^2	
**Refinement program**	SHELXL-2017/1 (Sheldrick, 2017)	
**Function minimized**	$\Sigma w(F_o^2 - F_c^2)^2$	
**Data / restraints / parameters**	4065 / 0 / 311	
**Goodness-of-fit on F^2**	1.162	
**Final R indices**	2935 data; I>2σ(I)	R1 = 0.1087, wR2 = 0.2406
	all data	R1 = 0.1425, wR2 = 0.2577
**Weighting scheme**	$w=1/[\sigma^2(F_o^2)+(0.0884P)^2+3.7712P]$<br>where $P=(F_o^2+2F_c^2)/3$	
**Largest diff. peak and hole**	0.387 and -0.243 $eÅ^{-3}$	
**R.M.S. deviation from mean**	0.055 $eÅ^{-3}$	

Table S5. Atomic coordinates and equivalent isotropic atomic displacement parameters (Å^2).
U(eq) is defined as one third of the trace of the orthogonalized U_{ij} tensor.

	x/a	y/b	z/c	U(eq)
N001	0.3124(2)	0.6960(4)	0.28070(19)	0.0379(9)
C002	0.3788(2)	0.7371(5)	0.3354(2)	0.0350(9)
C003	0.4272(2)	0.8516(5)	0.3050(3)	0.0395(10)
C004	0.3238(2)	0.7889(5)	0.2156(2)	0.0377(10)
C005	0.2542(2)	0.4494(5)	0.3511(2)	0.0391(10)
C006	0.2005(3)	0.4345(6)	0.4036(2)	0.0417(11)
C007	0.3111(3)	0.3241(6)	0.3502(3)	0.0437(11)
C008	0.3926(2)	0.8819(5)	0.2284(2)	0.0387(10)
C009	0.3129(3)	0.1898(6)	0.3996(3)	0.0494(12)
C00A	0.4145(3)	0.9839(6)	0.1723(3)	0.0508(12)
C00B	0.2779(3)	0.7881(6)	0.1451(3)	0.0501(12)
C00C	0.3954(3)	0.6873(6)	0.4097(3)	0.0508(12)
C00D	0.2607(3)	0.1740(6)	0.4505(3)	0.0492(12)
C00E	0.2056(3)	0.3001(6)	0.4512(3)	0.0475(12)
C00F	0.1632(3)	0.6327(6)	0.2395(3)	0.0495(12)
C00G	0.4942(3)	0.9146(6)	0.3494(3)	0.0514(13)
C00H	0.4622(3)	0.7515(7)	0.4518(3)	0.0570(14)
C00I	0.1279(3)	0.7872(7)	0.2444(3)	0.0555(13)
C00J	0.3677(3)	0.9882(6)	0.1043(3)	0.0580(14)
C00K	0.3012(3)	0.8900(7)	0.0911(3)	0.0593(14)
C00L	0.5108(3)	0.8645(7)	0.4224(3)	0.0588(14)
C00M	0.1255(3)	0.5075(7)	0.1911(3)	0.0569(14)
C00N	0.1592(4)	0.3516(7)	0.1832(3)	0.0648(15)
C00O	0.1397(3)	0.5660(7)	0.4111(3)	0.0628(15)
C00P	0.0514(3)	0.5413(9)	0.1449(3)	0.0692(17)
B1	0.2456(3)	0.5939(7)	0.2903(3)	0.0427(12)
C00R	0.3705(3)	0.3237(7)	0.2961(3)	0.0682(16)
C00S	0.2630(4)	0.0281(8)	0.5031(3)	0.0782(18)
C00T	0.0175(4)	0.7014(11)	0.1543(4)	0.087(2)
C00U	0.1626(4)	0.9253(7)	0.2921(4)	0.0753(17)
C00V	0.1230(4)	0.2334(8)	0.1338(3)	0.0798(19)
C00W	0.0514(5)	0.2671(11)	0.0899(4)	0.093(2)
C00X	0.0163(4)	0.4211(12)	0.0967(4)	0.094(2)
C00Y	0.0524(4)	0.8187(10)	0.2005(4)	0.0850(19)

Table S6. Bond lengths (Å).

N001-C004	1.410(5)	N001-C002	1.412(5)
N001-B1	1.422(6)	C002-C00C	1.369(6)

C002-C003	1.385(6)	C003-C00G	1.377(6)
C003-C008	1.422(6)	C004-C008	1.370(6)
C004-C00B	1.374(6)	C005-C007	1.386(6)
C005-C006	1.400(6)	C005-B1	1.568(7)
C006-C00E	1.356(6)	C006-C00O	1.486(7)
C007-C009	1.379(6)	C007-C00R	1.490(7)
C008-C00A	1.376(6)	C009-C00D	1.358(7)
C009-H009	0.93	C00A-C00J	1.346(7)
C00A-H00A	0.93	C00B-C00K	1.358(7)
C00B-H00B	0.93	C00C-C00H	1.360(7)
C00C-H00C	0.93	C00D-C00E	1.369(7)
C00D-C00S	1.486(7)	C00E-H00E	0.93
C00F-C00I	1.372(7)	C00F-C00M	1.406(7)
C00F-B1	1.577(7)	C00G-C00L	1.347(7)
C00G-H00G	0.93	C00H-C00L	1.370(7)
C00H-H00H	0.93	C00I-C00Y	1.418(8)
C00I-C00U	1.456(8)	C00J-C00K	1.361(7)
C00J-H00J	0.93	C00K-H00K	0.93
C00L-H00L	0.93	C00M-C00N	1.379(8)
C00M-C00P	1.420(7)	C00N-C00V	1.368(8)
C00N-H00N	0.93	C00O-H00D	0.96
C00O-H00F	0.96	C00O-H00I	0.96
C00P-C00X	1.360(9)	C00P-C00T	1.415(9)
C00R-H00M	0.96	C00R-H00O	0.96
C00R-H00P	0.96	C00S-H00Q	0.96
C00S-H00R	0.96	C00S-H00S	0.96
C00T-C00Y	1.323(9)	C00T-H00T	0.93
C00U-H00U	0.96	C00U-H00V	0.96
C00U-H00W	0.96	C00V-C00W	1.370(9)
C00V-H3	0.93	C00W-C00X	1.372(10)
C00W-H1	0.93	C00X-H00X	0.93
C00Y-H00Y	0.93		

Table S7. Bond angles (°).

C004-N001-C002	104.7(3)	C004-N001-B1	127.1(4)
C002-N001-B1	128.0(4)	C00C-C002-C003	121.1(4)
C00C-C002-N001	128.9(4)	C003-C002-N001	109.9(4)
C00G-C003-C002	119.9(4)	C00G-C003-C008	132.6(4)
C002-C003-C008	107.5(4)	C008-C004-C00B	120.5(4)
C008-C004-N001	111.1(4)	C00B-C004-N001	128.3(4)
C007-C005-C006	117.7(4)	C007-C005-B1	120.9(4)
C006-C005-B1	121.2(4)	C00E-C006-C005	119.6(4)
C00E-C006-C00O	118.6(4)	C005-C006-C00O	121.7(4)
C009-C007-C005	119.9(4)	C009-C007-C00R	117.1(4)
C005-C007-C00R	122.9(4)	C004-C008-C00A	120.5(4)
C004-C008-C003	106.8(4)	C00A-C008-C003	132.6(4)
C00D-C009-C007	122.7(4)	C00D-C009-H009	118.7
C007-C009-H009	118.7	C00J-C00A-C008	118.8(4)
C00J-C00A-H00A	120.6	C008-C00A-H00A	120.6
C00K-C00B-C004	117.3(4)	C00K-C00B-H00B	121.4
C004-C00B-H00B	121.4	C00H-C00C-C002	117.4(5)
C00H-C00C-H00C	121.3	C002-C00C-H00C	121.3
C009-C00D-C00E	116.5(4)	C009-C00D-C00S	122.1(5)
C00E-C00D-C00S	121.3(5)	C006-C00E-C00D	123.5(4)
C006-C00E-H00E	118.3	C00D-C00E-H00E	118.3
C00I-C00F-C00M	120.6(5)	C00I-C00F-B1	119.6(4)
C00M-C00F-B1	119.8(5)	C00L-C00G-C003	118.9(5)
C00L-C00G-H00G	120.5	C003-C00G-H00G	120.5
C00C-C00H-C00L	122.1(5)	C00C-C00H-H00H	119.0
C00L-C00H-H00H	119.0	C00F-C00I-C00Y	119.2(6)
C00F-C00I-C00U	124.6(5)	C00Y-C00I-C00U	116.2(5)
C00A-C00J-C00K	120.2(5)	C00A-C00J-H00J	119.9
C00K-C00J-H00J	119.9	C00B-C00K-C00J	122.5(5)
C00B-C00K-H00K	118.7	C00J-C00K-H00K	118.7
C00G-C00L-C00H	120.6(5)	C00G-C00L-H00L	119.7
C00H-C00L-H00L	119.7	C00N-C00M-C00F	122.7(5)
C00N-C00M-C00P	117.0(5)	C00F-C00M-C00P	120.3(6)
C00V-C00N-C00M	121.9(6)	C00V-C00N-H00N	119.1
C00M-C00N-H00N	119.1	C006-C00O-H00D	109.5
C006-C00O-H00F	109.5	H00D-C00O-H00F	109.5
C006-C00O-H00I	109.5	H00D-C00O-H00I	109.5
H00F-C00O-H00I	109.5	C00X-C00P-C00T	123.9(6)
C00X-C00P-C00M	120.0(7)	C00T-C00P-C00M	116.1(6)
N001-B1-C005	120.3(4)	N001-B1-C00F	118.1(4)
C005-B1-C00F	121.6(4)	C007-C00R-H00M	109.5
C007-C00R-H00O	109.5	H00M-C00R-H00O	109.5

C007-C00R-H00P	109.5	H00M-C00R-H00P	109.5
H00O-C00R-H00P	109.5	C00D-C00S-H00Q	109.5
C00D-C00S-H00R	109.5	H00Q-C00S-H00R	109.5
C00D-C00S-H00S	109.5	H00Q-C00S-H00S	109.5
H00R-C00S-H00S	109.5	C00Y-C00T-C00P	123.6(6)
C00Y-C00T-H00T	118.2	C00P-C00T-H00T	118.2
C00I-C00U-H00U	109.5	C00I-C00U-H00V	109.5
H00U-C00U-H00V	109.5	C00I-C00U-H00W	109.5
H00U-C00U-H00W	109.5	H00V-C00U-H00W	109.5
C00N-C00V-C00W	120.6(7)	C00N-C00V-H3	119.7
C00W-C00V-H3	119.7	C00V-C00W-C00X	118.8(6)
C00V-C00W-H1	120.6	C00X-C00W-H1	120.6
C00P-C00X-C00W	121.8(7)	C00P-C00X-H00X	119.1
C00W-C00X-H00X	119.1	C00T-C00Y-C00I	120.2(7)
C00T-C00Y-H00Y	119.9	C00I-C00Y-H00Y	119.9

Table S8. Torsion angles (°)

C004-N001-C002-C00C	176.8(4)	B1-N001-C002-C00C	1.1(7)
C004-N001-C002-C003	1.0(4)	B1-N001-C002-C003	-174.7(4)
C00C-C002-C003-C00G	1.3(6)	N001-C002-C003-C00G	177.5(4)
C00C-C002-C003-C008	-177.8(4)	N001-C002-C003-C008	-1.6(5)
C002-N001-C004-C008	0.0(4)	B1-N001-C004-C008	175.8(4)
C002-N001-C004-C00B	176.8(4)	B1-N001-C004-C00B	-7.5(7)
C007-C005-C006-C00E	-0.1(6)	B1-C005-C006-C00E	174.5(4)
C007-C005-C006-C00O	177.4(4)	B1-C005-C006-C00O	-8.0(7)
C006-C005-C007-C009	0.6(6)	B1-C005-C007-C009	-174.0(4)
C006-C005-C007-C00R	178.8(4)	B1-C005-C007-C00R	4.2(7)
C00B-C004-C008-C00A	3.9(7)	N001-C004-C008-C00A	-179.1(4)
C00B-C004-C008-C003	-178.0(4)	N001-C004-C008-C003	-1.0(5)
C00G-C003-C008-C004	-177.4(4)	C002-C003-C008-C004	1.5(5)
C00G-C003-C008-C00A	0.4(8)	C002-C003-C008-C00A	179.3(5)
C005-C007-C009-C00D	-0.3(7)	C00R-C007-C009-C00D	-178.6(5)
C004-C008-C00A-C00J	-1.2(7)	C003-C008-C00A-C00J	-178.7(5)
C008-C004-C00B-C00K	-3.7(7)	N001-C004-C00B-C00K	179.8(5)
C003-C002-C00C-C00H	-1.5(6)	N001-C002-C00C-C00H	-176.9(4)
C007-C009-C00D-C00E	-0.5(7)	C007-C009-C00D-C00S	179.6(5)
C005-C006-C00E-C00D	-0.7(7)	C00O-C006-C00E-C00D	-178.3(5)
C009-C00D-C00E-C006	1.0(7)	C00S-C00D-C00E-C006	-179.1(5)
C002-C003-C00G-C00L	-0.8(6)	C008-C003-C00G-C00L	178.0(5)
C002-C00C-C00H-C00L	1.3(7)	C00M-C00F-C00I-C00Y	0.6(7)
B1-C00F-C00I-C00Y	-177.8(5)	C00M-C00F-C00I-C00U	-179.1(5)
B1-C00F-C00I-C00U	2.5(7)	C008-C00A-C00J-C00K	-1.5(8)

C004-C00B-C00K-C00J	1.0(8)	C00A-C00J-C00K-C00B	1.6(9)
C003-C00G-C00L-C00H	0.6(7)	C00C-C00H-C00L-C00G	-0.9(8)
C00I-C00F-C00M-C00N	178.8(5)	B1-C00F-C00M-C00N	-2.9(7)
C00I-C00F-C00M-C00P	1.9(7)	B1-C00F-C00M-C00P	-179.7(4)
C00F-C00M-C00N-C00V	-177.6(5)	C00P-C00M-C00N-C00V	-0.7(8)
C00N-C00M-C00P-C00X	1.3(8)	C00F-C00M-C00P-C00X	178.3(5)
C00N-C00M-C00P-C00T	179.5(5)	C00F-C00M-C00P-C00T	-3.4(7)
C004-N001-B1-C005	157.1(4)	C002-N001-B1-C005	-28.1(7)
C004-N001-B1-C00F	-24.0(6)	C002-N001-B1-C00F	150.8(4)
C007-C005-B1-N001	-55.8(6)	C006-C005-B1-N001	129.8(5)
C007-C005-B1-C00F	125.3(5)	C006-C005-B1-C00F	-49.1(6)
C00I-C00F-B1-N001	-60.8(6)	C00M-C00F-B1-N001	120.9(5)
C00I-C00F-B1-C005	118.1(5)	C00M-C00F-B1-C005	-60.2(6)
C00X-C00P-C00T-C00Y	-179.1(6)	C00M-C00P-C00T-C00Y	2.7(9)
C00M-C00N-C00V-C00W	0.3(9)	C00N-C00V-C00W-C00X	-0.5(10)
C00T-C00P-C00X-C00W	-179.6(6)	C00M-C00P-C00X-C00W	-1.6(10)
C00V-C00W-C00X-C00P	1.2(10)	C00P-C00T-C00Y-C00I	-0.3(10)
C00F-C00I-C00Y-C00T	-1.4(9)	C00U-C00I-C00Y-C00T	178.4(6)

Table S9. Anisotropic atomic displacement parameters (Å^2).

The anisotropic atomic displacement factor exponent takes the form: $-2\pi^2[h^2 a^{*2} U_{11} + ... + 2 h k a^* b^* U_{12}]$

	U_{11}	U_{22}	U_{33}	U_{23}	U_{13}	U_{12}
N001	0.045(2)	0.0361(19)	0.0314(19)	-0.0019(15)	0.0019(16)	-0.0040(16)
C002	0.036(2)	0.031(2)	0.037(2)	-0.0059(18)	0.0010(18)	0.0021(18)
C003	0.035(2)	0.032(2)	0.053(3)	-0.013(2)	0.009(2)	0.0029(18)
C004	0.046(2)	0.032(2)	0.036(2)	-0.0008(18)	0.007(2)	-0.0047(19)
C005	0.040(2)	0.042(2)	0.035(2)	-0.0061(19)	0.0057(19)	-0.013(2)
C006	0.040(2)	0.049(3)	0.035(2)	0.001(2)	0.0023(19)	-0.003(2)
C007	0.049(3)	0.039(2)	0.047(3)	-0.008(2)	0.017(2)	-0.006(2)
C008	0.041(2)	0.033(2)	0.045(3)	-0.006(2)	0.013(2)	0.0001(19)
C009	0.057(3)	0.037(3)	0.054(3)	-0.004(2)	0.009(2)	0.004(2)
C00A	0.055(3)	0.045(3)	0.057(3)	-0.006(2)	0.025(3)	-0.014(2)
C00B	0.060(3)	0.050(3)	0.039(3)	-0.006(2)	0.004(2)	-0.018(2)
C00C	0.057(3)	0.050(3)	0.043(3)	-0.005(2)	-0.002(2)	0.003(2)
C00D	0.055(3)	0.048(3)	0.043(3)	0.002(2)	0.003(2)	-0.007(2)
C00E	0.046(3)	0.059(3)	0.039(3)	0.005(2)	0.011(2)	-0.004(2)
C00F	0.056(3)	0.056(3)	0.036(2)	0.007(2)	0.007(2)	-0.022(2)
C00G	0.035(2)	0.054(3)	0.066(3)	-0.018(3)	0.009(2)	-0.006(2)
C00H	0.054(3)	0.065(3)	0.046(3)	-0.013(3)	-0.012(2)	0.009(3)
C00I	0.050(3)	0.058(3)	0.059(3)	0.025(3)	0.009(2)	0.013(2)
C00J	0.074(3)	0.054(3)	0.049(3)	0.005(2)	0.020(3)	-0.015(3)

	U_{11}	U_{22}	U_{33}	U_{23}	U_{13}	U_{12}
C00K	0.078(4)	0.066(3)	0.034(3)	0.009(2)	0.009(2)	-0.013(3)
C00L	0.041(3)	0.071(4)	0.061(4)	-0.025(3)	-0.006(2)	-0.002(3)
C00M	0.063(3)	0.065(4)	0.044(3)	0.011(3)	0.010(2)	-0.018(3)
C00N	0.078(4)	0.067(4)	0.050(3)	-0.008(3)	0.012(3)	-0.023(3)
C00O	0.058(3)	0.074(4)	0.058(3)	0.015(3)	0.015(3)	0.013(3)
C00P	0.046(3)	0.102(5)	0.056(3)	0.012(3)	-0.004(3)	-0.027(3)
B1	0.051(3)	0.043(3)	0.034(3)	-0.006(2)	0.008(2)	-0.003(2)
C00R	0.082(4)	0.048(3)	0.083(4)	-0.007(3)	0.040(3)	0.000(3)
C00S	0.089(4)	0.075(4)	0.072(4)	0.031(3)	0.017(3)	0.005(3)
C00T	0.063(4)	0.116(6)	0.078(5)	0.023(4)	-0.006(3)	-0.006(4)
C00U	0.087(4)	0.055(4)	0.083(4)	0.004(3)	0.009(3)	0.010(3)
C00V	0.098(5)	0.081(4)	0.063(4)	-0.016(3)	0.022(4)	-0.034(4)
C00W	0.096(5)	0.112(6)	0.073(5)	-0.023(4)	0.020(4)	-0.052(5)
C00X	0.067(4)	0.134(7)	0.079(5)	-0.006(5)	0.003(4)	-0.037(5)
C00Y	0.076(4)	0.092(5)	0.086(5)	0.014(4)	0.004(4)	0.003(4)

Table S10. Hydrogen atomic coordinates and isotropic atomic displacement parameters (Å^2) for mazza2201.

	x/a	y/b	z/c	U(eq)
H009	0.3513	0.1067	0.3980	0.059
H00A	0.4607	1.0487	0.1813	0.061
H00B	0.2327	0.7205	0.1348	0.06
H00C	0.3624	0.6124	0.4306	0.061
H00E	0.1698	0.2932	0.4860	0.057
H00G	0.5275	0.9905	0.3294	0.062
H00H	0.4753	0.7177	0.5022	0.068
H00J	0.3809	1.0584	0.0661	0.07
H00K	0.2705	0.8928	0.0432	0.071
H00L	0.5555	0.9069	0.4532	0.071
H00N	0.2080	0.3261	0.2123	0.078
H00D	0.1121	0.5389	0.4530	0.094
H00F	0.1021	0.5707	0.3651	0.094
H00I	0.1655	0.6733	0.4198	0.094
H00M	0.3726	0.4334	0.2738	0.102
H00O	0.3550	0.2422	0.2568	0.102
H00P	0.4223	0.2952	0.3228	0.102
H00Q	0.2816	0.0646	0.5541	0.117
H00R	0.2985	-0.0562	0.4885	0.117
H00S	0.2103	-0.0184	0.5007	0.117
H00T	-0.0321	0.7255	0.1264	0.104
H00U	0.1821	0.8836	0.3420	0.113

	x/a	y/b	z/c	U(eq)
H00V	0.1226	1.0096	0.2954	0.113
H00W	0.2060	0.9737	0.2702	0.113
H3	0.1473	0.1293	0.1301	0.096
H1	0.0269	0.1871	0.0559	0.112
H00X	-0.0328	0.4438	0.0677	0.113
H00Y	0.0274	0.9221	0.2042	0.102

Compound 1a GS1

Method: b3lyp/6-311g(d,p) SCF Done: E(RB3LYP) = -1276.95634273 A.U. after 1 cycles
Lowest frequency = 22.5524

Zero-point correction= 0.480493 (Hartree/Particle)
Thermal correction to Energy= 0.508064
Thermal correction to Enthalpy= 0.509008
Thermal correction to Gibbs Free Energy= 0.421371
Sum of electronic and zero-point Energies= -1276.475850
Sum of electronic and thermal Energies= -1276.448279
Sum of electronic and thermal Enthalpies= -1276.447335
Sum of electronic and thermal Free Energies= -1276.534972

Standard orientation:

Center Number	Atomic Number	Atomic Type	Coordinates (Angstroms) X	Y	Z
1	5	0	0.096578	0.003873	0.211186
2	6	0	1.550181	1.573375	2.383269
3	1	0	0.655768	1.128377	2.827998
4	1	0	2.297217	0.779122	2.305599
5	1	0	1.929513	2.321810	3.081928
6	6	0	0.323039	2.161909	-1.208031
7	6	0	0.785367	3.465384	-1.399847
8	1	0	0.601811	3.951829	-2.353730
9	6	0	1.465657	4.161903	-0.402176
10	6	0	1.685898	3.510877	0.811576
11	1	0	2.204587	4.037410	1.607727
12	6	0	1.264501	2.198983	1.034162
13	6	0	0.563627	1.501352	0.021675
14	6	0	-0.429177	1.498114	-2.344291
15	1	0	-1.504408	1.466942	-2.144610
16	1	0	-0.282873	2.049929	-3.275060
17	1	0	-0.106872	0.467992	-2.517626
18	6	0	1.973297	5.563687	-0.636701
19	1	0	1.962818	6.151094	0.284596
20	1	0	3.006057	5.549907	-1.002591
21	1	0	1.369667	6.087517	-1.381420
22	7	0	-1.309092	-0.338612	0.097720
23	6	0	-2.417734	0.509592	0.377581
24	6	0	-1.858998	-1.574538	-0.351904
25	6	0	-3.623522	-0.182810	0.134776
26	6	0	-2.439489	1.809951	0.886187
27	6	0	-3.268080	-1.502087	-0.342149
28	6	0	-4.854921	0.431177	0.375060
29	6	0	-3.675515	2.407927	1.114783
30	1	0	-1.528536	2.350827	1.093854
31	6	0	-4.037668	-2.583373	-0.778804
32	6	0	-4.875401	1.731697	0.860930
33	1	0	-5.779945	-0.103040	0.188781
34	1	0	-3.703766	3.420203	1.501915
35	1	0	-5.120397	-2.525704	-0.767899
36	1	0	-5.821634	2.224597	1.052206
37	6	0	-1.996134	-3.778387	-1.276864
38	1	0	-1.505043	-4.667936	-1.654828

39	6	0	-1.214701	-2.710836	-0.845646
40	1	0	-0.138080	-2.774592	-0.894613
41	6	0	-3.395411	-3.724894	-1.238392
42	1	0	-3.975890	-4.573546	-1.581430
43	6	0	1.119307	-1.134110	0.593585
44	6	0	2.422655	-1.314254	0.011693
45	6	0	2.870293	-0.577428	-1.118493
46	6	0	3.312284	-2.298797	0.557486
47	6	0	1.654373	-2.885252	2.226419
48	6	0	4.112278	-0.790043	-1.667866
49	1	0	2.221764	0.174238	-1.547341
50	6	0	4.588336	-2.494408	-0.032978
51	6	0	2.899187	-3.064407	1.677717
52	1	0	1.341125	-3.474529	3.081013
53	6	0	4.983481	-1.758242	-1.121785
54	1	0	4.426816	-0.209515	-2.527958
55	1	0	5.247483	-3.243941	0.392957
56	1	0	3.584053	-3.798901	2.089245
57	1	0	5.960026	-1.917455	-1.565073
58	6	0	0.771307	-1.935192	1.673278
59	1	0	-0.210393	-1.822916	2.120893

Compound 1a GS2

Method: b3lyp/6-311g(d,p) SCF Done: E(RB3LYP) = -1276.95743337 A.U. after 1 cycles
Lowest frequency = 24.0210

Zero-point correction= 0.480319 (Hartree/Particle)
Thermal correction to Energy= 0.508035
Thermal correction to Enthalpy= 0.508979
Thermal correction to Gibbs Free Energy= 0.420667
Sum of electronic and zero-point Energies= -1276.477115
Sum of electronic and thermal Energies= -1276.449399
Sum of electronic and thermal Enthalpies= -1276.448455
Sum of electronic and thermal Free Energies= -1276.536766

Standard orientation:

Center Number	Atomic Number	Atomic Type	Coordinates (Angstroms) X	Y	Z
1	5	0	-0.275633	-0.380493	-0.199794
2	6	0	-1.439951	-2.061582	2.027863
3	1	0	-0.999666	-1.200545	2.540659
4	1	0	-0.612441	-2.696973	1.701296
5	1	0	-2.021127	-2.619762	2.764453
6	6	0	-2.655898	-0.441026	-1.225338
7	6	0	-3.977384	-0.895162	-1.237592
8	1	0	-4.622951	-0.608008	-2.062873
9	6	0	-4.491698	-1.697069	-0.220388
10	6	0	-3.638408	-2.051542	0.825950
11	1	0	-4.019359	-2.669440	1.634294
12	6	0	-2.306091	-1.639747	0.860798
13	6	0	-1.795416	-0.813434	-0.167152
14	6	0	-2.190994	0.454694	-2.355727
15	1	0	-2.107732	1.495646	-2.028313

16	1	0	-2.898867	0.426326	-3.186596
17	1	0	-1.212526	0.162004	-2.745611
18	6	0	-5.916775	-2.192984	-0.262460
19	1	0	-6.340975	-2.273338	0.741444
20	1	0	-5.972786	-3.187101	-0.719915
21	1	0	-6.554049	-1.526898	-0.848526
22	7	0	0.095020	1.015622	-0.124098
23	6	0	-0.669403	2.066748	0.465090
24	6	0	1.265420	1.631124	-0.659467
25	6	0	0.016191	3.291985	0.325715
26	6	0	-1.879691	2.010038	1.158607
27	6	0	1.238346	3.017159	-0.399443
28	6	0	-0.516128	4.468342	0.857714
29	6	0	-2.398320	3.193055	1.678523
30	1	0	-2.414119	1.081373	1.288440
31	6	0	2.265705	3.845977	-0.855521
32	6	0	-1.729519	4.414071	1.530813
33	1	0	0.013529	5.408226	0.749275
34	1	0	-3.341659	3.160748	2.211793
35	1	0	2.242348	4.910918	-0.652963
36	1	0	-2.158808	5.317094	1.949316
37	6	0	3.315896	1.914932	-1.860675
38	1	0	4.127645	1.491095	-2.440841
39	6	0	2.299416	1.075550	-1.414082
40	1	0	2.323075	0.023989	-1.654950
41	6	0	3.309039	3.286803	-1.581131
42	1	0	4.115602	3.913866	-1.943219
43	6	0	0.796315	-1.522790	-0.399245
44	6	0	1.973973	-1.696635	0.411632
45	6	0	2.283380	-0.848419	1.509139
46	6	0	2.868167	-2.782914	0.136994
47	6	0	1.450395	-3.500386	-1.694098
48	6	0	3.403610	-1.051878	2.279150
49	1	0	1.621753	-0.023810	1.741375
50	6	0	4.018441	-2.964481	0.948841
51	6	0	2.584591	-3.662278	-0.940125
52	1	0	1.230062	-4.183182	-2.507155
53	6	0	4.284204	-2.119329	1.997136
54	1	0	3.614375	-0.387342	3.109490
55	1	0	4.684969	-3.791556	0.726499
56	1	0	3.274995	-4.473642	-1.147210
57	1	0	5.165165	-2.268992	2.611103
58	6	0	0.554091	-2.449152	-1.403255
59	1	0	-0.352451	-2.365609	-1.993221

Compound 1a TS1-2rf-CC-carb
Method: b3lyp/6-311g(d,p) SCF Done: E(RB3LYP) = -1276.94888260 A.U. after 1 cycles
Lowest frequency = -31.4981

Zero-point correction= 0.480862 (Hartree/Particle)
Thermal correction to Energy= 0.507373
Thermal correction to Enthalpy= 0.508317
Thermal correction to Gibbs Free Energy= 0.424247
Sum of electronic and zero-point Energies= -1276.468020
Sum of electronic and thermal Energies= -1276.441510
Sum of electronic and thermal Enthalpies= -1276.440566

Sum of electronic and thermal Free Energies= -1276.524636

Standard orientation:

Center Number	Atomic Number	Atomic Type	Coordinates (Angstroms) X	Y	Z
1	5	0	0.006631	0.215887	0.297266
2	6	0	-0.577588	1.540743	-2.425450
3	1	0	0.436240	1.740678	-2.783700
4	1	0	-0.697093	0.459287	-2.334748
5	1	0	-1.268796	1.878749	-3.201009
6	6	0	-1.082297	2.375470	1.286723
7	6	0	-1.582323	3.672906	1.158589
8	1	0	-1.898580	4.201318	2.053988
9	6	0	-1.705193	4.299669	-0.079354
10	6	0	-1.354248	3.563034	-1.208088
11	1	0	-1.487454	4.005917	-2.191429
12	6	0	-0.858461	2.259730	-1.122469
13	6	0	-0.678856	1.649457	0.141231
14	6	0	-1.047531	1.780179	2.679078
15	1	0	-0.122568	2.038937	3.203010
16	1	0	-1.877489	2.163793	3.277175
17	1	0	-1.134469	0.691188	2.667527
18	6	0	-2.204374	5.719267	-0.193465
19	1	0	-1.372802	6.431120	-0.145961
20	1	0	-2.721980	5.885990	-1.141199
21	1	0	-2.892424	5.967580	0.618241
22	7	0	-0.731852	-1.006946	0.148625
23	6	0	-2.144657	-1.142779	-0.076637
24	6	0	-0.216702	-2.347795	0.201201
25	6	0	-2.483076	-2.507703	-0.167461
26	6	0	-3.138388	-0.168723	-0.187034
27	6	0	-1.264939	-3.268577	0.005922
28	6	0	-3.803502	-2.906720	-0.379714
29	6	0	-4.452122	-0.582296	-0.398562
30	1	0	-2.914440	0.882229	-0.111851
31	6	0	-1.021935	-4.643055	0.005262
32	6	0	-4.789078	-1.935758	-0.497437
33	1	0	-4.053334	-3.959486	-0.448529
34	1	0	-5.227056	0.170756	-0.486123
35	1	0	-1.835774	-5.343308	-0.145659
36	1	0	-5.820564	-2.225058	-0.662488
37	6	0	1.314954	-4.180303	0.390829
38	1	0	2.326413	-4.540396	0.540489
39	6	0	1.087158	-2.806006	0.393201
40	1	0	1.911979	-2.130687	0.544268
41	6	0	0.275743	-5.096409	0.199896
42	1	0	0.485780	-6.159767	0.203106
43	6	0	1.550328	0.221046	0.708958
44	6	0	2.635665	0.275165	-0.232461
45	6	0	2.443047	0.086968	-1.627019
46	6	0	3.980065	0.460932	0.231299
47	6	0	3.197055	0.428998	2.516653
48	6	0	3.491343	0.132740	-2.514894
49	1	0	1.448527	-0.126534	-1.993227
50	6	0	5.040024	0.521848	-0.711083
51	6	0	4.229862	0.547435	1.624354
52	1	0	3.387371	0.465073	3.583765

53	6	0	4.805327	0.369372	-2.054975
54	1	0	3.311152	-0.022860	-3.572738
55	1	0	6.049385	0.678456	-0.344416
56	1	0	5.249313	0.688062	1.968184
57	1	0	5.625862	0.412242	-2.762321
58	6	0	1.870250	0.270307	2.056119
59	1	0	1.089307	0.181052	2.799691

Compound 1a TS2-2rf-CN-mes
Method: b3lyp/6-311g(d,p) SCF Done: E(RB3LYP) = -1276.92582172 A.U. after 1 cycles
Lowest frequency = -33.9649

Zero-point correction= 0.480387 (Hartree/Particle)
Thermal correction to Energy= 0.506856
Thermal correction to Enthalpy= 0.507801
Thermal correction to Gibbs Free Energy= 0.423393
Sum of electronic and zero-point Energies= -1276.445435
Sum of electronic and thermal Energies= -1276.418965
Sum of electronic and thermal Enthalpies= -1276.418021
Sum of electronic and thermal Free Energies= -1276.502429

Standard orientation:

Center Number	Atomic Number	Atomic Type	Coordinates (Angstroms) X	Y	Z
1	5	0	0.420650	-0.180473	-0.400506
2	6	0	3.319425	0.709579	-1.105353
3	1	0	2.732806	0.965556	-1.986816
4	1	0	3.144048	1.495024	-0.368645
5	1	0	4.375071	0.745505	-1.379641
6	6	0	1.521309	-2.492564	0.160032
7	6	0	2.656373	-3.286264	0.320256
8	1	0	2.528931	-4.314783	0.642098
9	6	0	3.943055	-2.816932	0.071971
10	6	0	4.075915	-1.509440	-0.387129
11	1	0	5.067059	-1.135276	-0.622569
12	6	0	2.982405	-0.664706	-0.566958
13	6	0	1.651446	-1.121049	-0.261998
14	6	0	0.210073	-3.200446	0.437222
15	1	0	-0.487742	-3.124804	-0.396447
16	1	0	0.402862	-4.259939	0.614539
17	1	0	-0.296553	-2.807404	1.319917
18	6	0	5.148778	-3.691277	0.295543
19	1	0	5.964903	-3.427632	-0.380693
20	1	0	5.521610	-3.574751	1.319367
21	1	0	4.908836	-4.747248	0.153915
22	7	0	-0.970440	-0.654027	-0.041853
23	6	0	-1.573929	-0.570495	1.217054
24	6	0	-1.980684	-0.940870	-0.962468
25	6	0	-2.973161	-0.776115	1.096507
26	6	0	-0.989166	-0.369604	2.471178
27	6	0	-3.233714	-1.018124	-0.302667
28	6	0	-3.783744	-0.745478	2.234344
29	6	0	-1.816069	-0.347536	3.588836
30	1	0	0.080363	-0.250483	2.584283

31	6	0	-4.385860	-1.327798	-1.030277
32	6	0	-3.202640	-0.525671	3.476486
33	1	0	-4.854119	-0.897884	2.148141
34	1	0	-1.375524	-0.192954	4.567437
35	1	0	-5.348284	-1.387160	-0.533759
36	1	0	-3.820150	-0.500003	4.366745
37	6	0	-3.033800	-1.529168	-3.031167
38	1	0	-2.967180	-1.749808	-4.090655
39	6	0	-1.873514	-1.224159	-2.327925
40	1	0	-0.913185	-1.236844	-2.829580
41	6	0	-4.282769	-1.571406	-2.394130
42	1	0	-5.168990	-1.813186	-2.969227
43	6	0	0.408880	1.328229	-0.912279
44	6	0	0.677204	2.434360	-0.033544
45	6	0	1.139698	2.254835	1.298555
46	6	0	0.508290	3.778698	-0.500967
47	6	0	-0.131195	2.943056	-2.676476
48	6	0	1.395749	3.321680	2.125764
49	1	0	1.313711	1.249846	1.663258
50	6	0	0.769026	4.860525	0.380810
51	6	0	0.097628	4.003097	-1.839195
52	1	0	-0.438790	3.111341	-3.702672
53	6	0	1.199183	4.642585	1.665850
54	1	0	1.750002	3.150568	3.136082
55	1	0	0.625985	5.871904	0.014161
56	1	0	-0.026709	5.023343	-2.186814
57	1	0	1.395525	5.479176	2.326872
58	6	0	0.016653	1.617793	-2.209035
59	1	0	-0.189720	0.816454	-2.906830

Compound 1a TS3-2rf-CN-Np0
Method: b3lyp/6-311g(d,p) SCF Done: E(RB3LYP) = -1276.92690737 A.U. after 1 cycles
Lowest frequency = -42.2349

Zero-point correction= 0.480211 (Hartree/Particle)
Thermal correction to Energy= 0.506851
Thermal correction to Enthalpy= 0.507796
Thermal correction to Gibbs Free Energy= 0.423074
Sum of electronic and zero-point Energies= -1276.446696
Sum of electronic and thermal Energies= -1276.420056
Sum of electronic and thermal Enthalpies= -1276.419112
Sum of electronic and thermal Free Energies= -1276.503834

Standard orientation:

Center Number	Atomic Number	Atomic Type	Coordinates (Angstroms) X	Y	Z
1	5	0	0.236447	-0.551598	-0.000056
2	6	0	1.872190	-0.984283	-2.568387
3	1	0	0.864897	-1.404896	-2.529105
4	1	0	1.799597	0.021571	-2.988837
5	1	0	2.449857	-1.591686	-3.269121
6	6	0	2.539015	-0.974493	1.209626
7	6	0	3.924969	-1.148959	1.187984
8	1	0	4.449296	-1.256559	2.133597

9	6	0	4.647627	-1.212208	0.000315
10	6	0	3.925198	-1.148932	-1.187487
11	1	0	4.449706	-1.256509	-2.133001
12	6	0	2.539245	-0.974456	-1.209390
13	6	0	1.814787	-0.834924	0.000047
14	6	0	1.871695	-0.984363	2.568494
15	1	0	0.864412	-1.404978	2.529006
16	1	0	2.449228	-1.591787	3.269320
17	1	0	1.799013	0.021477	2.988964
18	6	0	6.149623	-1.356409	0.000465
19	1	0	6.499821	-1.893164	-0.884551
20	1	0	6.636282	-0.374799	0.000895
21	1	0	6.499584	-1.893812	0.885184
22	7	0	-0.219653	0.895106	-0.000076
23	6	0	-0.298361	1.729750	-1.125426
24	6	0	-0.298769	1.729575	1.125378
25	6	0	-0.336923	3.088844	-0.720974
26	6	0	-0.443256	1.388909	-2.473615
27	6	0	-0.337184	3.088732	0.721126
28	6	0	-0.438079	4.102539	-1.678188
29	6	0	-0.547264	2.413356	-3.407993
30	1	0	-0.507484	0.357783	-2.790196
31	6	0	-0.438672	4.102275	1.678465
32	6	0	-0.527963	3.761348	-3.020849
33	1	0	-0.462256	5.142866	-1.372526
34	1	0	-0.662704	2.160404	-4.456073
35	1	0	-0.462740	5.142651	1.372959
36	1	0	-0.610427	4.536976	-3.773320
37	6	0	-0.548434	2.412820	3.407969
38	1	0	-0.664215	2.159703	4.455972
39	6	0	-0.444098	1.388519	2.473468
40	1	0	-0.508386	0.357342	2.789871
41	6	0	-0.529018	3.760872	3.021041
42	1	0	-0.611745	4.536382	3.773606
43	6	0	-0.776958	-1.735873	-0.000098
44	6	0	-2.228863	-1.660390	-0.000155
45	6	0	-2.962889	-0.444692	-0.000091
46	6	0	-2.991910	-2.878272	-0.000270
47	6	0	-0.953093	-4.197086	-0.000254
48	6	0	-4.339441	-0.432768	-0.000154
49	1	0	-2.431168	0.492001	-0.000003
50	6	0	-4.410212	-2.832627	-0.000333
51	6	0	-2.323648	-4.128010	-0.000319
52	1	0	-0.448522	-5.156400	-0.000294
53	6	0	-5.077123	-1.633908	-0.000279
54	1	0	-4.861572	0.517459	-0.000102
55	1	0	-4.957869	-3.769335	-0.000423
56	1	0	-2.920919	-5.034514	-0.000406
57	1	0	-6.160782	-1.606776	-0.000325
58	6	0	-0.201285	-3.010276	-0.000146
59	1	0	0.878980	-3.086807	-0.000109

Compound 1a TS4-2rf-CN-Np180
Method: b3lyp/6-311g(d,p) SCF Done: E(RB3LYP) = -1276.92805367 A.U. after 1 cycles
Lowest frequency = -41.3938

Zero-point correction= 0.480199
(Hartree/Particle)
Thermal correction to Energy= 0.506839
Thermal correction to Enthalpy= 0.507783
Thermal correction to Gibbs Free Energy= 0.423007
Sum of electronic and zero-point Energies= -1276.447855
Sum of electronic and thermal Energies= -1276.421215
Sum of electronic and thermal Enthalpies= -1276.420271
Sum of electronic and thermal Free Energies= -1276.505047

Standard orientation:

Center Number	Atomic Number	Atomic Type	Coordinates (Angstroms) X	Y	Z
1	5	0	-0.190911	-0.159018	-0.000044
2	6	0	-0.758622	1.423827	2.563690
3	1	0	-1.094973	0.385842	2.504115
4	1	0	0.236828	1.429319	3.013906
5	1	0	-1.432727	1.942763	3.249260
6	6	0	-0.764923	2.101105	-1.210050
7	6	0	-1.012991	3.475346	-1.188470
8	1	0	-1.150721	3.993033	-2.133866
9	6	0	-1.113008	4.193389	0.000119
10	6	0	-1.013075	3.475229	1.188641
11	1	0	-1.150869	3.992822	2.134078
12	6	0	-0.765021	2.100980	1.210100
13	6	0	-0.584993	1.388166	-0.000003
14	6	0	-0.758410	1.424085	-2.563706
15	1	0	-1.432317	1.943193	-3.249338
16	1	0	0.237114	1.429464	-3.013757
17	1	0	-1.094933	0.386146	-2.504284
18	6	0	-1.335389	5.685760	0.000158
19	1	0	-0.380246	6.222470	-0.000102
20	1	0	-1.890012	6.007540	-0.884612
21	1	0	-1.889543	6.007498	0.885226
22	7	0	1.294773	-0.496602	-0.000024
23	6	0	2.132168	-0.520860	1.124696
24	6	0	2.132237	-0.520758	-1.124695
25	6	0	3.491307	-0.464017	0.720923
26	6	0	1.803137	-0.686531	2.473676
27	6	0	3.491351	-0.463954	-0.720834
28	6	0	4.509596	-0.494709	1.677926
29	6	0	2.832541	-0.718973	3.407983
30	1	0	0.779158	-0.820884	2.790934
31	6	0	4.509698	-0.494569	-1.677777
32	6	0	4.175995	-0.607331	3.020836
33	1	0	5.549075	-0.447337	1.371963
34	1	0	2.588127	-0.850023	4.456322
35	1	0	5.549159	-0.447224	-1.371748
36	1	0	4.955630	-0.635749	3.773166
37	6	0	2.832747	-0.718697	-3.407954
38	1	0	2.588397	-0.849667	-4.456318
39	6	0	1.803287	-0.686328	-2.473707
40	1	0	0.779327	-0.820664	-2.791034
41	6	0	4.176178	-0.607083	-3.020717
42	1	0	4.955859	-0.635441	-3.773002
43	6	0	-1.163814	-1.376915	-0.000083
44	6	0	-2.615933	-1.330798	-0.000075

45	6	0	-3.369894	-0.128272	-0.000072
46	6	0	-3.355872	-2.561703	-0.000077
47	6	0	-1.291384	-3.839799	-0.000127
48	6	0	-4.746932	-0.139743	-0.000060
49	1	0	-2.853115	0.818628	-0.000078
50	6	0	-4.774431	-2.540979	-0.000060
51	6	0	-2.662624	-3.798537	-0.000103
52	1	0	-0.767583	-4.788670	-0.000152
53	6	0	-5.462790	-1.353893	-0.000050
54	1	0	-5.286538	0.800857	-0.000058
55	1	0	-5.305893	-3.486953	-0.000059
56	1	0	-3.241496	-4.716879	-0.000105
57	1	0	-6.546767	-1.346384	-0.000038
58	6	0	-0.561624	-2.638369	-0.000114
59	1	0	0.519129	-2.692818	-0.000122

Compound 1b GS1

Method: b3lyp/6-311g(d,p) SCF Done: E(RB3LYP) = -1316.27847872 A.U. after 1 cycles

Lowest frequency = 25.6218

Zero-point correction= 0.507983 (Hartree/Particle)
Thermal correction to Energy= 0.537216
Thermal correction to Enthalpy= 0.538160
Thermal correction to Gibbs Free Energy= 0.447361
Sum of electronic and zero-point Energies= -1315.770496
Sum of electronic and thermal Energies= -1315.741263
Sum of electronic and thermal Enthalpies= -1315.740318
Sum of electronic and thermal Free Energies= -1315.831117

Standard orientation:

Center Number	Atomic Number	Atomic Type	Coordinates (Angstroms) X	Y	Z
1	5	0	0.117008	0.093074	0.219289
2	6	0	1.530645	1.671654	2.429945
3	1	0	0.638500	1.242700	2.893898
4	1	0	2.262963	0.866565	2.335251
5	1	0	1.934799	2.413129	3.121931
6	6	0	0.294575	2.290745	-1.156969
7	6	0	0.729685	3.607462	-1.321046
8	1	0	0.541553	4.106421	-2.267535
9	6	0	1.388485	4.301327	-0.308231
10	6	0	1.618256	3.631738	0.893060
11	1	0	2.123264	4.153661	1.700838
12	6	0	1.227059	2.306518	1.089132
13	6	0	0.543048	1.608924	0.062455
14	6	0	-0.438688	1.649999	-2.319546
15	1	0	-1.521470	1.671687	-2.163857
16	1	0	-0.229809	2.190973	-3.244863
17	1	0	-0.161757	0.605629	-2.477132
18	6	0	1.866159	5.717784	-0.514311
19	1	0	1.837617	6.287944	0.417379
20	1	0	2.901069	5.732809	-0.873968
21	1	0	1.255586	6.241598	-1.253342
22	7	0	-1.263795	-0.290676	-0.001770

23	6	0	-2.420200	0.513677	0.212525
24	6	0	-1.739729	-1.558712	-0.442285
25	6	0	-3.582514	-0.234898	-0.071998
26	6	0	-2.523197	1.817476	0.702347
27	6	0	-3.149436	-1.547688	-0.500687
28	6	0	-4.849316	0.327766	0.099276
29	6	0	-3.793558	2.363752	0.863261
30	1	0	-1.647765	2.399923	0.946935
31	6	0	-3.850900	-2.672895	-0.939934
32	6	0	-4.949504	1.633034	0.562079
33	1	0	-5.740272	-0.249777	-0.120642
34	1	0	-3.883700	3.378314	1.234592
35	1	0	-4.934287	-2.661910	-0.983264
36	1	0	-5.924214	2.086608	0.700147
37	6	0	-1.739164	-3.795399	-1.294805
38	1	0	-1.193408	-4.674847	-1.617288
39	6	0	-1.024499	-2.681446	-0.863709
40	1	0	0.054835	-2.699080	-0.860462
41	6	0	-3.139341	-3.799865	-1.328921
42	1	0	-3.666278	-4.682575	-1.672453
43	6	0	1.172146	-1.019486	0.626213
44	6	0	2.393170	-1.213233	-0.117462
45	6	0	0.959523	-1.787784	1.773096
46	6	0	2.685114	-0.496827	-1.311416
47	6	0	3.355758	-2.183455	0.312014
48	6	0	1.937714	-2.727752	2.191376
49	6	0	3.842670	-0.714068	-2.020736
50	1	0	1.987026	0.248059	-1.665787
51	6	0	4.542238	-2.386295	-0.439073
52	6	0	3.094261	-2.927330	1.487931
53	1	0	1.753735	-3.299311	3.095620
54	6	0	4.785861	-1.668932	-1.583443
55	1	0	4.033182	-0.145444	-2.924174
56	1	0	5.255082	-3.126833	-0.090699
57	1	0	3.825118	-3.655888	1.824100
58	1	0	5.694791	-1.832479	-2.151335
59	6	0	-0.283398	-1.690190	2.633457
60	1	0	-1.024940	-2.435507	2.325910
61	1	0	-0.037619	-1.889738	3.679484
62	1	0	-0.769603	-0.716874	2.585086

Compound 1b GS2
Method: b3lyp/6-311g(d,p) SCF Done: E(RB3LYP) = -1316.27841809 A.U. after 1 cycles
Lowest frequency = 26.6137

Zero-point correction= 0.507816 (Hartree/Particle)
Thermal correction to Energy= 0.537189
Thermal correction to Enthalpy= 0.538133
Thermal correction to Gibbs Free Energy= 0.446609
Sum of electronic and zero-point Energies= -1315.770602
Sum of electronic and thermal Energies= -1315.741229
Sum of electronic and thermal Enthalpies= -1315.740285
Sum of electronic and thermal Free Energies= -1315.831809

Standard orientation:

Center Number	Atomic Number	Atomic Type	Coordinates (Angstroms) X	Y	Z
1	5	0	-0.262781	-0.338111	-0.229466
2	6	0	-1.366113	-2.266517	1.872109
3	1	0	-0.776729	-1.519914	2.409928
4	1	0	-0.657222	-2.962148	1.416299
5	1	0	-1.949218	-2.819580	2.611052
6	6	0	-2.724476	-0.179378	-1.054494
7	6	0	-4.064701	-0.569727	-1.008217
8	1	0	-4.755653	-0.154684	-1.736617
9	6	0	-4.542229	-1.466133	-0.054374
10	6	0	-3.628939	-1.983294	0.863895
11	1	0	-3.978002	-2.677185	1.623313
12	6	0	-2.275569	-1.642747	0.835023
13	6	0	-1.796996	-0.718535	-0.126869
14	6	0	-2.327734	0.828803	-2.114157
15	1	0	-2.279431	1.839482	-1.697313
16	1	0	-3.062635	0.842277	-2.921856
17	1	0	-1.352860	0.618393	-2.556463
18	6	0	-5.991147	-1.887548	-0.033715
19	1	0	-6.334055	-2.086484	0.984545
20	1	0	-6.140140	-2.805799	-0.612735
21	1	0	-6.636454	-1.119810	-0.466650
22	7	0	0.142147	1.050676	-0.153870
23	6	0	-0.557867	2.112808	0.492109
24	6	0	1.313082	1.637213	-0.720682
25	6	0	0.151265	3.321617	0.329124
26	6	0	-1.719955	2.076222	1.265608
27	6	0	1.331632	3.021951	-0.452874
28	6	0	-0.318510	4.504430	0.904210
29	6	0	-2.175914	3.264893	1.829220
30	1	0	-2.263695	1.157769	1.426242
31	6	0	2.364275	3.826819	-0.938783
32	6	0	-1.489843	4.472039	1.649184
33	1	0	0.228958	5.431562	0.776276
34	1	0	-3.081731	3.247819	2.424628
35	1	0	2.374896	4.890871	-0.730527
36	1	0	-1.869912	5.380154	2.102790
37	6	0	3.338993	1.872535	-1.973769
38	1	0	4.126823	1.428913	-2.571916
39	6	0	2.315516	1.057073	-1.499483
40	1	0	2.313739	0.002862	-1.730044
41	6	0	3.371275	3.244497	-1.696675
42	1	0	4.181961	3.852528	-2.081291
43	6	0	0.832367	-1.473289	-0.418358
44	6	0	1.866044	-1.639483	0.576736
45	6	0	0.796619	-2.357403	-1.498453
46	6	0	1.978435	-0.797689	1.719858
47	6	0	2.823899	-2.697208	0.451556
48	6	0	1.768742	-3.385130	-1.610736
49	6	0	2.961012	-0.985274	2.662370
50	1	0	1.279786	0.017766	1.851110
51	6	0	3.826768	-2.867972	1.441607
52	6	0	2.747989	-3.557224	-0.669596
53	1	0	1.724716	-4.049611	-2.467494
54	6	0	3.898818	-2.032149	2.526876
55	1	0	3.017597	-0.320871	3.517401
56	1	0	4.538754	-3.678279	1.321645

57	1	0	3.475780	-4.355879	-0.771383
58	1	0	4.668937	-2.170405	3.277280
59	6	0	-0.215110	-2.239296	-2.618295
60	1	0	-1.236082	-2.172131	-2.240945
61	1	0	-0.156985	-3.102681	-3.283833
62	1	0	-0.032025	-1.348527	-3.228763

Compound 1b TS1-2rf-CC-carb
Method: b3lyp/6-311g(d,p) SCF Done: E(RB3LYP) = -1316.26419669 A.U. after 1 cycles
Lowest frequency = -44.2319

Zero-point correction= 0.508394 (Hartree/Particle)
Thermal correction to Energy= 0.536434
Thermal correction to Enthalpy= 0.537378
Thermal correction to Gibbs Free Energy= 0.450529
Sum of electronic and zero-point Energies= -1315.755802
Sum of electronic and thermal Energies= -1315.727763
Sum of electronic and thermal Enthalpies= -1315.726818
Sum of electronic and thermal Free Energies= -1315.813668

Standard orientation:

Center Number	Atomic Number	Atomic Type	Coordinates (Angstroms) X	Y	Z
1	5	0	-0.110530	-0.141289	0.260742
2	6	0	-0.961151	-1.486762	-2.414443
3	1	0	-1.813796	-0.832115	-2.605250
4	1	0	-0.054161	-0.881495	-2.470528
5	1	0	-0.915331	-2.212932	-3.229193
6	6	0	-0.593092	-2.533655	1.255393
7	6	0	-1.165758	-3.806039	1.176325
8	1	0	-1.144043	-4.440136	2.058591
9	6	0	-1.732726	-4.294094	0.003811
10	6	0	-1.645708	-3.483982	-1.124888
11	1	0	-2.008400	-3.862531	-2.076550
12	6	0	-1.082028	-2.206302	-1.088244
13	6	0	-0.592169	-1.671703	0.131013
14	6	0	0.078733	-2.182554	2.565903
15	1	0	-0.628106	-1.770187	3.289328
16	1	0	0.510983	-3.080259	3.014221
17	1	0	0.889802	-1.465274	2.438088
18	6	0	-2.392275	-5.649883	-0.049383
19	1	0	-3.466509	-5.569671	0.150955
20	1	0	-2.278757	-6.111529	-1.033361
21	1	0	-1.971362	-6.328970	0.695972
22	7	0	1.258059	0.244719	0.056897
23	6	0	2.367144	-0.612713	-0.260296
24	6	0	1.808776	1.568154	0.159133
25	6	0	3.551295	0.147856	-0.333520
26	6	0	2.406128	-1.989017	-0.488713
27	6	0	3.198397	1.525694	-0.068115
28	6	0	4.774553	-0.456412	-0.627120
29	6	0	3.634573	-2.578100	-0.781539
30	1	0	1.517601	-2.596555	-0.444558
31	6	0	3.967945	2.689240	-0.021727

32	6	0	4.811594	-1.826411	-0.850468
33	1	0	5.680097	0.137358	-0.680982
34	1	0	3.669908	-3.646797	-0.960072
35	1	0	5.036985	2.646674	-0.197206
36	1	0	5.751627	-2.314606	-1.080418
37	6	0	1.961476	3.939063	0.466686
38	1	0	1.478295	4.887812	0.670530
39	6	0	1.181207	2.785623	0.424427
40	1	0	0.118832	2.851184	0.590969
41	6	0	3.342353	3.898864	0.249134
42	1	0	3.921995	4.813900	0.288660
43	6	0	-1.257772	0.921046	0.640702
44	6	0	-2.068557	1.510805	-0.400471
45	6	0	-1.601645	1.208018	1.958965
46	6	0	-1.628058	1.580435	-1.748574
47	6	0	-3.304525	2.161149	-0.085300
48	6	0	-2.830265	1.862087	2.255592
49	6	0	-2.384602	2.177138	-2.731150
50	1	0	-0.650083	1.193268	-2.000295
51	6	0	-4.084574	2.732254	-1.122075
52	6	0	-3.685409	2.277064	1.274830
53	1	0	-3.084219	2.028787	3.298187
54	6	0	-3.642361	2.735755	-2.422789
55	1	0	-2.006633	2.227279	-3.746276
56	1	0	-5.030162	3.196974	-0.861669
57	1	0	-4.629806	2.750028	1.523337
58	1	0	-4.240262	3.190315	-3.204620
59	6	0	-0.689394	0.960756	3.139248
60	1	0	-0.496859	1.913576	3.643702
61	1	0	-1.140693	0.295839	3.881045
62	1	0	0.276632	0.553220	2.851324

Compound 1b TS2-2rf-CN-mes
Method: b3lyp/6-311g(d,p)
SCF Done: E(RB3LYP) = -1316.24403875 A.U. after 1 cycles
Lowest frequency = -37.4028

Zero-point correction= 0.507999
(Hartree/Particle)
Thermal correction to Energy= 0.535980
Thermal correction to Enthalpy= 0.536924
Thermal correction to Gibbs Free Energy= 0.449729
Sum of electronic and zero-point Energies= -1315.736040
Sum of electronic and thermal Energies= -1315.708059
Sum of electronic and thermal Enthalpies= -1315.707115
Sum of electronic and thermal Free Energies= -1315.794310

Standard orientation:

Center Number	Atomic Number	Atomic Type	Coordinates (Angstroms) X	Y	Z
1	5	0	0.542626	0.033231	0.243554
2	6	0	3.028180	-1.723535	0.856203
3	1	0	2.597019	-1.684109	1.856787
4	1	0	2.416178	-2.412882	0.274625
5	1	0	4.026466	-2.156195	0.941919
6	6	0	2.282219	1.762397	-0.689960

7	6	0	3.601104	2.125779	-0.961591
8	1	0	3.788875	3.089015	-1.424745
9	6	0	4.683823	1.303036	-0.666114
10	6	0	4.416895	0.068682	-0.079461
11	1	0	5.246772	-0.591098	0.152514
12	6	0	3.123099	-0.352962	0.218870
13	6	0	1.994444	0.489754	-0.079919
14	6	0	1.246109	2.794594	-1.087087
15	1	0	0.656236	3.140115	-0.237716
16	1	0	1.748054	3.663500	-1.516276
17	1	0	0.546465	2.415797	-1.832842
18	6	0	6.097073	1.742113	-0.946337
19	1	0	6.527149	2.234711	-0.067075
20	1	0	6.738362	0.891886	-1.189266
21	1	0	6.138118	2.454159	-1.773173
22	7	0	-0.647985	0.919113	-0.095461
23	6	0	-1.470597	0.826268	-1.227241
24	6	0	-1.315216	1.743375	0.819805
25	6	0	-2.682028	1.531928	-1.006677
26	6	0	-1.213884	0.246170	-2.473609
27	6	0	-2.582431	2.119892	0.305666
28	6	0	-3.653574	1.595236	-2.009029
29	6	0	-2.191945	0.325920	-3.458454
30	1	0	-0.274795	-0.241818	-2.689041
31	6	0	-3.416478	2.964970	1.042397
32	6	0	-3.409853	0.981739	-3.230139
33	1	0	-4.581647	2.129644	-1.837367
34	1	0	-2.000817	-0.123730	-4.426421
35	1	0	-4.387944	3.249578	0.653019
36	1	0	-4.153356	1.024102	-4.017543
37	6	0	-1.704822	3.117323	2.745837
38	1	0	-1.360217	3.535101	3.685351
39	6	0	-0.860239	2.274512	2.030771
40	1	0	0.133204	2.064811	2.400795
41	6	0	-2.980091	3.450884	2.267920
42	1	0	-3.615716	4.109648	2.848120
43	6	0	0.083667	-1.338571	0.932208
44	6	0	-0.295708	-2.453050	0.098932
45	6	0	-0.103589	-1.458222	2.304732
46	6	0	0.085607	-2.537154	-1.266952
47	6	0	-0.998850	-3.570260	0.651161
48	6	0	-0.794614	-2.580422	2.838803
49	6	0	-0.256214	-3.612777	-2.052796
50	1	0	0.703218	-1.752726	-1.687215
51	6	0	-1.366103	-4.653034	-0.187489
52	6	0	-1.267244	-3.585264	2.042311
53	1	0	-0.949333	-2.626272	3.912600
54	6	0	-1.012254	-4.675351	-1.514593
55	1	0	0.061525	-3.648087	-3.088862
56	1	0	-1.917590	-5.480241	0.247798
57	1	0	-1.811299	-4.421884	2.468116
58	1	0	-1.291163	-5.513803	-2.142663
59	6	0	0.443903	-0.470414	3.311782
60	1	0	-0.348695	0.121660	3.776337
61	1	0	0.958889	-1.009039	4.113835
62	1	0	1.166476	0.216863	2.869144

Compound 1b TS3-2rf-CN-2MeNp0

Method: b3lyp/6-311g(d,p) SCF Done: E(RB3LYP) = -1316.23895524 A.U. after 1 cycles
Lowest frequency = -39.7846

Zero-point correction= 0.507745 (Hartree/Particle)
Thermal correction to Energy= 0.535883
Thermal correction to Enthalpy= 0.536827
Thermal correction to Gibbs Free Energy= 0.449291
Sum of electronic and zero-point Energies= -1315.731211
Sum of electronic and thermal Energies= -1315.703072
Sum of electronic and thermal Enthalpies= -1315.702128
Sum of electronic and thermal Free Energies= -1315.789664

Standard orientation:

Center Number	Atomic Number	Atomic Type	Coordinates (Angstroms) X	Y	Z
1	5	0	-0.107622	0.600664	-0.189394
2	6	0	-2.011563	0.819675	-2.603361
3	1	0	-0.963230	1.111501	-2.703059
4	1	0	-2.103962	-0.210332	-2.954608
5	1	0	-2.589338	1.451438	-3.283382
6	6	0	-2.225502	1.330390	1.189328
7	6	0	-3.605555	1.515957	1.308482
8	1	0	-4.012992	1.765955	2.284557
9	6	0	-4.466404	1.415000	0.219943
10	6	0	-3.890871	1.174939	-1.025128
11	1	0	-4.524806	1.156760	-1.907742
12	6	0	-2.518006	0.979308	-1.184589
13	6	0	-1.653674	1.004416	-0.063993
14	6	0	-1.402175	1.559540	2.441101
15	1	0	-0.361863	1.802423	2.217094
16	1	0	-1.815041	2.394743	3.013394
17	1	0	-1.408283	0.683218	3.093367
18	6	0	-5.958563	1.573051	0.378721
19	1	0	-6.412622	2.004515	-0.516777
20	1	0	-6.437839	0.603247	0.552483
21	1	0	-6.204597	2.215054	1.227958
22	7	0	0.120147	-0.900115	-0.048455
23	6	0	-0.057662	-1.858626	-1.055938
24	6	0	0.158681	-1.598758	1.166781
25	6	0	-0.219561	-3.146205	-0.481331
26	6	0	0.031655	-1.711770	-2.444074
27	6	0	-0.083217	-2.978673	0.944364
28	6	0	-0.382477	-4.265192	-1.302768
29	6	0	-0.126735	-2.839628	-3.242007
30	1	0	0.248040	-0.753993	-2.896217
31	6	0	-0.071944	-3.875148	2.016866
32	6	0	-0.351062	-4.105659	-2.681715
33	1	0	-0.513335	-5.249450	-0.866372
34	1	0	-0.057780	-2.736830	-4.319279
35	1	0	-0.261301	-4.930025	1.850283
36	1	0	-0.471514	-4.965696	-3.330166
37	6	0	0.498826	-2.046073	3.495370
38	1	0	0.757882	-1.699169	4.489595
39	6	0	0.488882	-1.136525	2.444043
40	1	0	0.766166	-0.106251	2.614714

41	6	0	0.204669	-3.402652	3.292465
42	1	0	0.220740	-4.086832	4.132892
43	6	0	1.094285	1.593126	-0.325837
44	6	0	2.495179	1.156072	-0.263642
45	6	0	0.857668	2.991906	-0.353057
46	6	0	2.924277	-0.189508	-0.430839
47	6	0	3.541973	2.116815	-0.059108
48	6	0	1.924773	3.905478	-0.177105
49	6	0	4.250496	-0.552526	-0.349895
50	1	0	2.204176	-0.957097	-0.644475
51	6	0	4.898884	1.715250	0.041433
52	6	0	3.213614	3.488182	0.003860
53	1	0	1.702278	4.966580	-0.177234
54	6	0	5.255958	0.398836	-0.093203
55	1	0	4.519134	-1.593247	-0.492754
56	1	0	5.649982	2.479644	0.211563
57	1	0	4.007160	4.211600	0.163242
58	1	0	6.293834	0.094769	-0.021477
59	6	0	-0.486504	3.655626	-0.568882
60	1	0	-1.111024	3.626863	0.326269
61	1	0	-0.331167	4.704779	-0.828157
62	1	0	-1.061702	3.193574	-1.367263

Compound 1b TS4-2rf-CN-2MeNp180
Method: b3lyp/6-311g(d,p)
SCF Done: E(RB3LYP) = -1316.24091375 A.U. after 1 cycles
Lowest frequency = -41.3991

Zero-point correction= 0.507986
(Hartree/Particle)
Thermal correction to Energy= 0.536042
Thermal correction to Enthalpy= 0.536986
Thermal correction to Gibbs Free Energy= 0.449402
Sum of electronic and zero-point Energies= -1315.732928
Sum of electronic and thermal Energies= -1315.704872
Sum of electronic and thermal Enthalpies= -1315.703927
Sum of electronic and thermal Free Energies= -1315.791512

Standard orientation:

Center Number	Atomic Number	Atomic Type	Coordinates (Angstroms) X	Y	Z
1	5	0	0.253018	-0.180748	0.108583
2	6	0	0.904116	1.246081	-2.542132
3	1	0	1.263584	0.233934	-2.347739
4	1	0	-0.000935	1.174408	-3.149974
5	1	0	1.666245	1.746316	-3.145370
6	6	0	0.480550	2.234261	1.138281
7	6	0	0.508805	3.623359	0.998637
8	1	0	0.499848	4.236126	1.896239
9	6	0	0.575930	4.242688	-0.246719
10	6	0	0.686766	3.417244	-1.362201
11	1	0	0.817898	3.866616	-2.343063
12	6	0	0.664254	2.024052	-1.264781
13	6	0	0.497288	1.399450	-0.005193
14	6	0	0.526087	1.686308	2.549788

15	1	0	1.194366	2.294176	3.165556
16	1	0	-0.457937	1.697654	3.023673
17	1	0	0.905168	0.662316	2.581532
18	6	0	0.551779	5.745499	-0.379562
19	1	0	0.984782	6.231789	0.498030
20	1	0	1.105460	6.078116	-1.260935
21	1	0	-0.475733	6.111792	-0.481918
22	7	0	-1.226059	-0.540809	0.057365
23	6	0	-1.986682	-0.654794	-1.113853
24	6	0	-2.134983	-0.461338	1.119431
25	6	0	-3.369708	-0.569081	-0.808495
26	6	0	-1.568829	-0.928651	-2.420130
27	6	0	-3.465006	-0.445955	0.624876
28	6	0	-4.321735	-0.680473	-1.825488
29	6	0	-2.533769	-1.042158	-3.415402
30	1	0	-0.525040	-1.076647	-2.657944
31	6	0	-4.543903	-0.397652	1.511712
32	6	0	-3.899641	-0.903694	-3.129314
33	1	0	-5.379138	-0.609182	-1.594866
34	1	0	-2.219910	-1.256097	-4.431101
35	1	0	-5.560862	-0.379242	1.135130
36	1	0	-4.627364	-0.995854	-3.927129
37	6	0	-2.985535	-0.470683	3.361308
38	1	0	-2.810680	-0.511293	4.430741
39	6	0	-1.896819	-0.513391	2.496687
40	1	0	-0.895583	-0.610435	2.891895
41	6	0	-4.299995	-0.395725	2.878689
42	1	0	-5.127560	-0.362543	3.577691
43	6	0	1.328933	-1.314242	0.191850
44	6	0	2.766045	-1.014916	0.169326
45	6	0	0.954498	-2.684507	0.207081
46	6	0	3.307822	0.295769	0.265802
47	6	0	3.728743	-2.073488	0.065507
48	6	0	1.942736	-3.695798	0.111472
49	6	0	4.663912	0.536415	0.236087
50	1	0	2.647586	1.138375	0.369279
51	6	0	5.120160	-1.800933	0.025520
52	6	0	3.275491	-3.409230	0.022304
53	1	0	1.620836	-4.730788	0.106568
54	6	0	5.590194	-0.515636	0.105728
55	1	0	5.018797	1.557937	0.317921
56	1	0	5.805427	-2.637720	-0.061577
57	1	0	4.003912	-4.209677	-0.062346
58	1	0	6.654168	-0.310612	0.078420
59	6	0	-0.455706	-3.227949	0.328326
60	1	0	-1.051649	-3.037881	-0.565865
61	1	0	-0.412115	-4.309507	0.467661
62	1	0	-0.997030	-2.807667	1.174167

Compound 1c GS1
Method: b3lyp/6-311g(d,p) SCF Done: E(RB3LYP) = -1430.62089770 A.U. after 1 cycles
Lowest frequency = 24.8358

Zero-point correction= 0.526937 (Hartree/Particle)
Thermal correction to Energy= 0.557192
Thermal correction to Enthalpy= 0.558136

Thermal correction to Gibbs Free Energy= 0.465026
Sum of electronic and zero-point Energies= -1430.093960
Sum of electronic and thermal Energies= -1430.063705
Sum of electronic and thermal Enthalpies= -1430.062761
Sum of electronic and thermal Free Energies= -1430.155871

Standard orientation:

Center Number	Atomic Number	Atomic Type	Coordinates (Angstroms) X	Y	Z
1	6	0	0.431543	-2.071901	-2.438386
2	1	0	0.080057	-1.105519	-2.807479
3	1	0	1.478843	-1.942314	-2.153701
4	1	0	0.397641	-2.775196	-3.272820
5	6	0	-1.490668	-2.340195	0.879999
6	6	0	-1.928209	-3.662562	0.780285
7	1	0	-2.516919	-4.081211	1.591680
8	6	0	-1.641523	-4.455930	-0.328591
9	6	0	-0.880587	-3.890691	-1.351231
10	1	0	-0.650509	-4.486234	-2.230077
11	6	0	-0.396235	-2.583880	-1.278747
12	6	0	-0.704336	-1.774177	-0.156947
13	6	0	-1.895500	-1.566442	2.119878
14	1	0	-2.765342	-0.932729	1.922213
15	1	0	-2.166973	-2.253281	2.924335
16	1	0	-1.106096	-0.912288	2.495040
17	6	0	-2.110701	-5.887945	-0.407135
18	1	0	-2.322468	-6.182309	-1.437898
19	1	0	-1.345008	-6.571654	-0.023894
20	1	0	-3.015205	-6.044924	0.184923
21	7	0	-1.063209	0.793800	0.232340
22	6	0	-2.429462	0.870124	-0.168408
23	6	0	-0.780957	2.016218	0.909892
24	6	0	-2.979456	2.103272	0.242098
25	6	0	-3.189539	-0.024969	-0.924261
26	6	0	-1.936232	2.824825	0.939225
27	6	0	-4.301604	2.429270	-0.068209
28	6	0	-4.506546	0.314672	-1.221672
29	1	0	-2.780487	-0.962422	-1.269612
30	6	0	-1.920280	4.063436	1.584452
31	6	0	-5.065013	1.526112	-0.796011
32	1	0	-4.721586	3.377218	0.249098
33	1	0	-5.107492	-0.377452	-1.800578
34	1	0	-2.809791	4.683152	1.604959
35	1	0	-6.093147	1.761879	-1.045470
36	6	0	0.385443	3.665840	2.192208
37	1	0	1.288983	3.998281	2.690482
38	6	0	0.385294	2.427919	1.556392
39	1	0	1.272198	1.813034	1.569949
40	6	0	-0.751573	4.483173	2.205234
41	1	0	-0.719415	5.441637	2.710374
42	6	0	1.410558	-0.003605	-0.136234
43	6	0	2.359778	-0.659913	0.691681
44	6	0	1.880801	0.889376	-1.139534
45	6	0	1.980770	-1.558424	1.740645
46	6	0	3.773333	-0.409090	0.528961
47	6	0	3.297175	1.116957	-1.308465
48	6	0	1.006288	1.577412	-2.042807

49	6	0	2.907298	-2.160444	2.546348
50	1	0	0.933846	-1.777756	1.891633
51	6	0	4.711309	-1.056711	1.391102
52	6	0	4.200881	0.467793	-0.467264
53	6	0	3.756497	2.001860	-2.332469
54	1	0	-0.062185	1.436275	-1.955512
55	6	0	1.483240	2.416205	-3.012080
56	6	0	4.295488	-1.908489	2.373093
57	1	0	2.581282	-2.839173	3.326529
58	1	0	5.767085	-0.851234	1.247499
59	1	0	5.264208	0.650084	-0.591669
60	6	0	2.879041	2.636492	-3.162786
61	1	0	4.825724	2.155641	-2.435526
62	1	0	0.788601	2.921274	-3.673829
63	1	0	5.015863	-2.393260	3.022227
64	1	0	3.238526	3.305259	-3.936570
65	5	0	-0.145135	-0.300984	-0.012842

Compound 1c TS1-2rf-CC-carb

Method: b3lyp/6-311g(d,p) SCF Done: E(RB3LYP) = -1430.60831448 A.U. after 1 cycles

Lowest frequency = -32.0870

Zero-point correction= 0.527201 (Hartree/Particle)
Thermal correction to Energy= 0.556349
Thermal correction to Enthalpy= 0.557293
Thermal correction to Gibbs Free Energy= 0.467963
Sum of electronic and zero-point Energies= -1430.081113
Sum of electronic and thermal Energies= -1430.051966
Sum of electronic and thermal Enthalpies= -1430.051022
Sum of electronic and thermal Free Energies= -1430.140352

Standard orientation:

Center Number	Atomic Number	Atomic Type	Coordinates (Angstroms) X	Y	Z
1	6	0	-0.592072	-1.878776	-2.567869
2	1	0	-1.151628	-0.942189	-2.524013
3	1	0	0.385796	-1.665086	-3.004267
4	1	0	-1.122198	-2.539370	-3.257631
5	6	0	-0.474883	-2.534972	1.209337
6	6	0	-0.515453	-3.931414	1.187138
7	1	0	-0.579056	-4.462946	2.132606
8	6	0	-0.503616	-4.657974	0.000366
9	6	0	-0.515858	-3.931635	-1.186540
10	1	0	-0.579762	-4.463358	-2.131881
11	6	0	-0.475271	-2.535196	-1.209051
12	6	0	-0.396879	-1.799143	0.000069
13	6	0	-0.591267	-1.878206	2.568015
14	1	0	-1.150485	-0.941440	2.524043
15	1	0	-1.121554	-2.538446	3.257996
16	1	0	0.386768	-1.664800	3.004203
17	6	0	-0.497326	-6.166628	0.000506
18	1	0	-0.996641	-6.568728	-0.884383
19	1	0	0.528068	-6.552757	0.000501
20	1	0	-0.996568	-6.568565	0.885510

21	7	0	-1.276710	0.715646	-0.000107
22	6	0	-2.679004	0.398835	0.000006
23	6	0	-1.203867	2.151246	-0.000228
24	6	0	-3.435250	1.587960	-0.000033
25	6	0	-3.323279	-0.839113	0.000138
26	6	0	-2.503207	2.694881	-0.000181
27	6	0	-4.830299	1.549873	0.000049
28	6	0	-4.716648	-0.861082	0.000220
29	1	0	-2.772872	-1.765194	0.000183
30	6	0	-2.701082	4.076542	-0.000272
31	6	0	-5.469543	0.317124	0.000175
32	1	0	-5.403600	2.470069	0.000015
33	1	0	-5.222013	-1.820144	0.000321
34	1	0	-3.704674	4.486696	-0.000229
35	1	0	-6.552261	0.266219	0.000242
36	6	0	-0.304885	4.371012	-0.000479
37	1	0	0.555166	5.030761	-0.000600
38	6	0	-0.092649	2.994199	-0.000387
39	1	0	0.913016	2.607704	-0.000440
40	6	0	-1.593601	4.914134	-0.000419
41	1	0	-1.725761	5.989941	-0.000492
42	6	0	1.357801	0.276486	-0.000011
43	6	0	2.079488	0.417923	1.213862
44	6	0	2.079510	0.417540	-1.213921
45	6	0	1.427554	0.626576	2.469813
46	6	0	3.524359	0.449119	1.213431
47	6	0	3.524382	0.448736	-1.213461
48	6	0	1.427606	0.625705	-2.469950
49	6	0	2.130217	0.785653	3.632743
50	1	0	0.349468	0.711735	2.486904
51	6	0	4.222693	0.565270	2.454620
52	6	0	4.210198	0.403168	0.000000
53	6	0	4.222739	0.564474	-2.454675
54	1	0	0.349528	0.710850	-2.487060
55	6	0	2.130286	0.784367	-3.632930
56	6	0	3.550284	0.725279	3.632372
57	1	0	1.602609	0.969624	4.561952
58	1	0	5.307585	0.553971	2.435658
59	1	0	5.295831	0.396212	0.000012
60	6	0	3.550354	0.724041	-3.632502
61	1	0	5.307631	0.553199	-2.435688
62	1	0	1.602694	0.967987	-4.562217
63	1	0	4.093351	0.832519	4.564532
64	1	0	4.093439	0.830958	-4.564688
65	5	0	-0.176822	-0.206196	-0.000010

Compound 1c TS2-2rf-CN-mes
Method: b3lyp/6-311g(d,p) SCF Done: E(RB3LYP) = -1430.58677999 A.U. after 1 cycles
Lowest frequency = -35.0641

Zero-point correction= 0.526701 (Hartree/Particle)
Thermal correction to Energy= 0.555858
Thermal correction to Enthalpy= 0.556803
Thermal correction to Gibbs Free Energy= 0.466838
Sum of electronic and zero-point Energies= -1430.060079
Sum of electronic and thermal Energies= -1430.030922

```
Sum of electronic and thermal Enthalpies=          -1430.029977
Sum of electronic and thermal Free Energies=       -1430.119942
```

Standard orientation:

Center Number	Atomic Number	Atomic Type	Coordinates (Angstroms) X	Y	Z
1	6	0	2.164328	2.759027	0.045558
2	1	0	1.543339	2.909078	-0.837913
3	1	0	1.521427	2.894633	0.915463
4	1	0	2.913988	3.551985	0.061285
5	6	0	2.995582	-1.037612	0.037238
6	6	0	4.384908	-0.919096	0.048423
7	1	0	4.982910	-1.824587	0.055740
8	6	0	5.034219	0.312005	0.053289
9	6	0	4.245828	1.459948	0.053636
10	1	0	4.732975	2.429641	0.065142
11	6	0	2.853723	1.410599	0.042378
12	6	0	2.170280	0.143012	0.031652
13	6	0	2.479006	-2.461913	0.035256
14	1	0	1.881198	-2.685498	-0.848948
15	1	0	3.323672	-3.152922	0.046757
16	1	0	1.860673	-2.681007	0.906300
17	6	0	6.537567	0.400850	0.032818
18	1	0	6.903668	0.464409	-0.998043
19	1	0	6.892181	1.289562	0.559586
20	1	0	6.996377	-0.478677	0.489225
21	7	0	-0.107505	-1.267172	-0.000392
22	6	0	-0.618816	-1.913510	-1.135067
23	6	0	-0.650859	-1.920469	1.115188
24	6	0	-1.524206	-2.934184	-0.746507
25	6	0	-0.294214	-1.738489	-2.483786
26	6	0	-1.544724	-2.938639	0.694700
27	6	0	-2.145240	-3.729901	-1.712619
28	6	0	-0.920972	-2.543752	-3.428397
29	1	0	0.436549	-1.009368	-2.801041
30	6	0	-2.192870	-3.740358	1.637766
31	6	0	-1.849259	-3.524917	-3.053527
32	1	0	-2.841323	-4.506781	-1.415494
33	1	0	-0.677097	-2.411637	-4.476605
34	1	0	-2.880169	-4.515288	1.316113
35	1	0	-2.322463	-4.135487	-3.813741
36	6	0	-1.017736	-2.565304	3.395013
37	1	0	-0.803568	-2.439935	4.450514
38	6	0	-0.364539	-1.754131	2.473654
39	1	0	0.356953	-1.027187	2.816148
40	6	0	-1.935029	-3.543910	2.987796
41	1	0	-2.429537	-4.159229	3.730415
42	6	0	-0.418469	1.289455	0.002140
43	6	0	-0.973755	1.777742	1.210684
44	6	0	-0.936991	1.786015	-1.219329
45	6	0	-0.357480	1.547627	2.481135
46	6	0	-2.159960	2.601421	1.195039
47	6	0	-2.123304	2.609612	-1.233869
48	6	0	-0.282800	1.564309	-2.472229
49	6	0	-0.888473	2.036296	3.642743
50	1	0	0.585707	1.015385	2.511725
51	6	0	-2.709107	3.057304	2.432932

52	6	0	-2.730957	2.956652	-0.027130
53	6	0	-2.635034	3.073814	-2.484653
54	1	0	0.660833	1.032038	-2.477969
55	6	0	-0.778683	2.060838	-3.645952
56	6	0	-2.098260	2.782306	3.623083
57	1	0	-0.383472	1.860500	4.585888
58	1	0	-3.616916	3.651011	2.403657
59	1	0	-3.637795	3.553348	-0.038816
60	6	0	-1.988617	2.806801	-3.657702
61	1	0	-3.543427	3.667322	-2.478707
62	1	0	-0.245557	1.891299	-4.574660
63	1	0	-2.519331	3.147049	4.553084
64	1	0	-2.381583	3.177786	-4.597483
65	5	0	0.618358	0.069217	0.013403

Compound 1c TS3-2rf-CN-An
Method: b3lyp/6-311g(d,p)
SCF Done: E(RB3LYP) = -1430.58203331 A.U. after 1 cycles
Lowest frequency = -37.8568

Zero-point correction= 0.526557 (Hartree/Particle)
Thermal correction to Energy= 0.555831
Thermal correction to Enthalpy= 0.556776
Thermal correction to Gibbs Free Energy= 0.466997
Sum of electronic and zero-point Energies= -1430.055476
Sum of electronic and thermal Energies= -1430.026202
Sum of electronic and thermal Enthalpies= -1430.025258
Sum of electronic and thermal Free Energies= -1430.115036

Standard orientation:

Center Number	Atomic Number	Atomic Type	Coordinates (Angstroms) X	Y	Z
1	6	0	-0.021035	-2.291883	-2.526402
2	1	0	-0.450754	-1.305610	-2.718327
3	1	0	1.029744	-2.261675	-2.822342
4	1	0	-0.528371	-2.998928	-3.188118
5	6	0	-0.714502	-2.304799	1.243213
6	6	0	-0.733079	-3.685999	1.456821
7	1	0	-0.988561	-4.053293	2.447275
8	6	0	-0.462118	-4.600725	0.444354
9	6	0	-0.224131	-4.087713	-0.828335
10	1	0	-0.078658	-4.773885	-1.658539
11	6	0	-0.189646	-2.715424	-1.081776
12	6	0	-0.385459	-1.784154	-0.032196
13	6	0	-1.112085	-1.435859	2.418639
14	1	0	-1.466334	-0.452603	2.107508
15	1	0	-1.923984	-1.910937	2.975313
16	1	0	-0.279419	-1.291816	3.111633
17	6	0	-0.442965	-6.086096	0.709159
18	1	0	0.566406	-6.423547	0.969472
19	1	0	-1.100191	-6.352599	1.540413
20	1	0	-0.758833	-6.653370	-0.169914
21	7	0	1.278705	0.226630	-0.090621
22	6	0	1.904112	0.464807	1.141696
23	6	0	2.299528	0.119687	-1.043283

24	6	0	3.313838	0.421897	0.990280
25	6	0	1.336640	0.802676	2.373755
26	6	0	3.568369	0.204530	-0.412002
27	6	0	4.146017	0.646591	2.090783
28	6	0	2.182854	1.026640	3.453644
29	1	0	0.268888	0.921586	2.486600
30	6	0	4.737377	0.153415	-1.176040
31	6	0	3.576705	0.935329	3.323363
32	1	0	5.224160	0.609686	1.978064
33	1	0	1.753065	1.293541	4.412849
34	1	0	5.707776	0.210311	-0.694900
35	1	0	4.209420	1.115094	4.184805
36	6	0	3.385615	0.035359	-3.179935
37	1	0	3.325540	0.003194	-4.262178
38	6	0	2.209355	0.078397	-2.438936
39	1	0	1.253248	0.105744	-2.942454
40	6	0	4.642293	0.053267	-2.557828
41	1	0	5.541107	0.017082	-3.162407
42	6	0	-1.298540	0.855243	-0.439818
43	6	0	-2.688789	0.462970	-0.407333
44	6	0	-1.016326	2.272530	-0.394911
45	6	0	-3.152487	-0.850187	-0.720322
46	6	0	-3.713862	1.422494	-0.071533
47	6	0	-2.058006	3.210828	-0.043118
48	6	0	0.249373	2.852112	-0.715858
49	6	0	-4.480040	-1.189998	-0.657521
50	1	0	-2.446665	-1.598611	-1.035671
51	6	0	-5.083501	1.029105	0.016786
52	6	0	-3.358028	2.750965	0.151985
53	6	0	-1.777155	4.606059	0.077400
54	1	0	1.046227	2.225957	-1.074413
55	6	0	0.476827	4.201561	-0.625582
56	6	0	-5.465100	-0.250661	-0.264425
57	1	0	-4.779891	-2.197756	-0.922894
58	1	0	-5.815592	1.780617	0.292963
59	1	0	-4.128546	3.460372	0.440339
60	6	0	-0.534655	5.096935	-0.198045
61	1	0	-2.585767	5.267488	0.370058
62	1	0	1.451969	4.589581	-0.897895
63	1	0	-6.506697	-0.544779	-0.208433
64	1	0	-0.325196	6.157170	-0.117340
65	5	0	-0.173874	-0.208762	-0.253440
